# Screen time, reward sensitivity, and mood dysregulation among Egyptian adolescents: concurrent associations and a test of construct distinctiveness

**DOI:** 10.3389/fpsyg.2026.2006153

**Published:** 2026-10-28

**Authors:** Masooma Suhail Al-Mutairi, Shanoo Sheikh, Rania Fawzy, Kayaty Ashour

**Affiliations:** 1 College of Education, Kuwait University, Kuwait City, Kuwait; 2 Bachelor of Science in Clinical Psychology Program, Department of Health Sciences, College of Health and Rehabilitation Sciences, Princess Nourah bint Abdulrahman University, Riyadh, Saudi Arabia; 3 Department of Psychology, Faculty of Arts, Beni-Suef University, Beni-Suef, Egypt; 4 Department of Sociology, Faculty of Arts, Beni-Suef University, Beni-Suef, Egypt

**Keywords:** adolescents, common-method variance, discriminant validity, media psychology, mood dysregulation, reward sensitivity, screen time

## Abstract

This cross-sectional study examined concurrent associations among self-reported daily screen time, reward sensitivity, and mood dysregulation, and tested whether the two psychological self-report constructs are empirically distinguishable, in a convenience sample of 640 Egyptian adolescents aged 13–19 years. Participants reported typical daily hours in four activity domains and completed six reward-sensitivity items adapted from Behavioral Activation System content and seven mood-dysregulation items modelled on the Affective Reactivity Index. Summed screen time (M = 9.96 h per day, an upper-bound estimate because activities could overlap) correlated with mood dysregulation [*r* = 0.53, 95% CI (0.47, 0.58)] and reward sensitivity [*r* = 0.63 (0.58, 0.67)]. The two psychological scales, however, correlated *r* = 0.94; confirmatory factor analyses estimated their latent correlation at 0.997, the heterotrait–monotrait ratio was 0.98, and a one-factor model fitted essentially as well as the two-factor model, so discriminant validity was not supported. In covariate-adjusted hierarchical regression with heteroskedasticity-consistent standard errors, screen time was associated with mood dysregulation [*B* = 0.035 (0.024, 0.047), Δ*R*^2^ = 0.017] until reward sensitivity was entered, after which it was not [*B* = −0.004 (−0.010, 0.001)]; with the entry order reversed, screen time added Δ*R*^2^ < 0.001 beyond reward sensitivity. Given the measurement overlap, this attenuation is interpreted as the consequence of entering a near-equivalent self-report dimension, not as evidence that reward sensitivity explains mood dysregulation. Demographic covariates, chiefly parental education and school type, which were strongly confounded, accounted for much of the outcome variance. The findings indicate a positive concurrent link in the full sample between reported screen time and a single reward–affective reactivity self-report dimension and underline the need for psychometrically discriminating, multimethod, and longitudinal designs.

## Introduction

Adolescence is a developmentally sensitive period in which emotional reactivity, reward responsivity, self-evaluative concerns, and regulatory control undergo substantial reorganization. Digital media are now embedded in adolescents’ daily routines, making screen use not merely a lifestyle variable but a salient context in which social feedback, novelty, reward anticipation, self-presentation, and affect regulation can occur. Contemporary reviews therefore caution against treating screen time as a unitary exposure and instead emphasize the psychological processes that accompany particular patterns of use (Orben et al., 2024; Santos et al., 2023; Weinstein, 2023).

Associations between more screen use and poorer emotional functioning have nevertheless been reported across multiple designs. A meta-analysis of cohort studies linked higher screen time with increased depression risk over time, while systematic-review evidence indicates recurrent associations with internalizing symptoms and poorer well-being, although the magnitude and direction of effects vary by platform, activity, duration, and psychosocial context (Li et al., 2022; Santos et al., 2023). More recent work further suggests that social comparison and maladaptive emotion regulation may be important correlates of poorer mental health among social-media users (Castillo-Gualda et al., 2026).

A complementary literature focuses on reward-related and cognitive-control processes. Longitudinal neuroimaging studies have associated greater screen use or habitual social-media checking with developmental differences in neural systems implicated in salience, reward anticipation, and inhibitory control (Chen et al., 2023; Maza et al., 2023). Reviews of problematic internet and smartphone use likewise identify alterations in cognitive-control and reward-processing networks among adolescents and young adults (Méndez et al., 2024; Shanmugasundaram and Tamilarasu, 2023). These findings do not establish that ordinary screen exposure causes neurocognitive change, but they strengthen the rationale for examining individual differences in responsiveness to rewarding digital cues.

Reward sensitivity refers to the degree to which individuals are attentive to, motivated by, or behaviorally drawn toward rewarding stimuli. This construct is especially relevant during adolescence, when reward responsivity may be heightened relative to still-developing regulatory-control capacities. Digital environments can supply frequent and emotionally salient rewards through social approval, novelty, intermittent feedback, and game-related incentives. Empirical work links reward responsiveness to daily affective functioning and reward-related cognitive control in adolescents, while recent accounts of digital engagement emphasize reinforcement processes as one potential route to persistent use (Duttweiler et al., 2024; Jose et al., 2026; Qiu et al., 2025).

Mood dysregulation, the third focal construct, refers to difficulty modulating emotional intensity, duration, and expression in developmentally appropriate ways. It is increasingly treated as a transdiagnostic process relevant to multiple forms of adolescent psychopathology (Paulus et al., 2021; Zhou et al., 2026). In digital contexts, adolescents may use screens for distraction, reassurance, stimulation, avoidance, or mood modification. Longitudinal evidence indicates that problematic technology use and negative emotional processes can be bidirectionally related, underscoring the need to avoid simple one-way causal accounts (Su et al., 2026; Pazdur et al., 2025).

The present study brings these strands together. Its general objective is to estimate the concurrent associations among screen time, reward sensitivity, and mood dysregulation in Egyptian adolescents and to evaluate whether the covariate-adjusted association between screen time and mood dysregulation changes when reward sensitivity is added to the model. Because that question presupposes that reward sensitivity and mood dysregulation are distinct constructs, the study first tests their empirical distinctiveness at the item level. The aim is not to infer a causal pathway or a mechanism from concurrent data: reward sensitivity is examined as a theoretically relevant correlate and candidate mechanism, not as an observed one, and the regression is treated as an analysis of conditional associations.

### Research problem

The literature on adolescent digital behavior has moved beyond the binary question of whether screen time is simply beneficial or harmful. Evidence increasingly suggests that the psychological meaning of screen use depends on what adolescents do online, how they respond to rewarding and socially salient cues, and how effectively they regulate affect (Orben et al., 2024; Santos et al., 2023). Yet many studies still isolate exposure duration from the motivational and emotional processes that may accompany it.

A second problem is inferential. Cross-sectional associations are often described using causal or mediational language even when temporal ordering has not been established, and jointly analyzed self-report constructs are rarely tested for discriminant validity before their associations are interpreted. The present study therefore focuses on concurrent covariation and hierarchical statistical adjustment. Specifically, it asks whether the reward-sensitivity and mood-dysregulation scales can be empirically distinguished, whether screen time is associated with mood dysregulation and reward sensitivity, whether reward sensitivity is associated with mood dysregulation, and whether the screen-time coefficient attenuates when reward sensitivity is introduced after demographic and contextual covariates. Because the two psychological scales are extremely highly correlated in the present data, construct distinctiveness is tested directly rather than treated only as an interpretive limitation.

### Research questions

1. Are reward sensitivity and mood dysregulation empirically distinguishable at the item level in this sample?
2. What is the association between total daily screen time and mood dysregulation among adolescents?
3. What is the association between total daily screen time and reward sensitivity?
4. To what extent is reward sensitivity associated with mood dysregulation?
5. After adjustment for demographic and contextual characteristics, how does the conditional association between screen time and mood dysregulation change when reward sensitivity is added to the model, and how much variance does screen time share with mood dysregulation beyond reward sensitivity when the entry order is reversed?
6. Do screen time, reward sensitivity, and mood dysregulation differ by gender, school/institution type, and educational level, and are their associations similar in early/middle and late adolescence?

#### Hypotheses

Based on the reviewed literature, we tested five primary hypotheses:

*H1:* Screen time will show a positive concurrent association with mood dysregulation.

*H2:* Screen time will show a positive concurrent association with reward sensitivity.

*H3:* Reward sensitivity will be positively associated with mood dysregulation.

*H4:* The covariate-adjusted association between screen time and mood dysregulation will be attenuated upon accounting for reward sensitivity.

*H5:* Reward sensitivity and mood dysregulation will represent empirically distinct constructs, establishing a structural precondition for interpreting H4.

In addition, group differences and age-band comparisons were evaluated descriptively without directional hypotheses. Hypotheses H1–H5 were formulated prior to data analysis, and the study was not preregistered.

## Literature review

### Screen time and adolescent mental health

Across cross-sectional, longitudinal, and review-based studies, screen use is associated with adolescent mental-health outcomes in ways that are statistically detectable but heterogeneous. Li et al. (2022) reported a prospective association between screen time and depression risk in a meta-analysis of cohort studies, while Santos et al. (2023) concluded that associations with mental health vary according to activity type, duration, and psychosocial context. This heterogeneity is important: a total-hours measure can capture exposure burden, but it cannot distinguish socially supportive, educational, recreational, passive, or compulsive use. Duration is also a crude and error-prone metric: self-reported and device-logged use correlate only moderately (Parry et al., 2021), and associations between total technology use and adolescent well-being are typically small (Orben and Przybylski, 2019). Self-reported hours are therefore better treated as a relative index of use than as evidence that any particular amount is intrinsically problematic.

Mechanism-focused accounts therefore emphasize social comparison, sensitivity to exclusion and approval, self-concept formation, and emotion-regulation style rather than duration alone. Orben et al. (2024) argue that developmental vulnerabilities can interact with the affordances of social media, while Castillo-Gualda et al. (2026) show that maladaptive emotion regulation and social comparison are associated with poorer mental health among social-media users. These perspectives motivate analysis of individual susceptibility alongside exposure quantity.

### Screen time, cognitive control, and reward-related processing

A major theoretical concern is the balance between reward drive and cognitive control. Chen et al. (2023) linked greater daily screen use in preadolescence with higher reward orientation and longitudinal differences in inhibitory-control circuitry. Maza et al. (2023) similarly found that habitual social-media checking was associated with developmental change in neural systems involved in salience, reward, and control. Reviews of fMRI studies of problematic internet and smartphone use further identify cognitive-control and reward-processing differences in adolescents and young adults (Méndez et al., 2024). These findings provide a developmental rationale for considering reward sensitivity, while leaving open questions about directionality and generalization to ordinary screen use.

Digital platforms can also provide reinforcement architectures characterized by rapid feedback, novelty, social affirmation, and intermittent rewards. Jose et al. (2026) describe an increasingly algorithmically mediated attention ecology, and Giansanti (2025) reviews behavioral reinforcement and emotional-regulation needs in youth smartphone addiction. Such accounts shift attention from screen quantity toward the processes that can make some forms of use particularly compelling.

### Reward sensitivity as an adolescent vulnerability factor

Reward sensitivity is particularly salient in adolescence because developmental models commonly identify heightened responsiveness to social and novelty-based rewards during this period. Duttweiler et al. (2024) associated neural reward responsiveness with daily positive-affect functioning in adolescent girls, whereas Qiu et al. (2025) identified behavioral and neural dysfunctions in reward-related cognitive control among adolescents with major depressive disorder. These studies support a close connection between reward processing and affective functioning, although they do not imply that reward sensitivity is itself pathological.

In screen-based contexts, adolescents high in reward sensitivity may be especially responsive to notifications, approval cues, novelty, competitive incentives, and variable feedback schedules. Prospective work has identified psychological predictors of adolescent screen use and problematic screen use (Grund and Luciana, 2025), and longitudinal reviews identify multiple risk factors for problematic social-media use (Pazdur et al., 2025). The current study therefore treats reward sensitivity as a theoretically relevant correlate and candidate mechanism whose statistical contribution should be evaluated alongside screen time rather than assumed to mediate it.

### Mood dysregulation in adolescence

Mood dysregulation involves difficulty modulating emotional responses in intensity, duration, and behavioral expression. Paulus et al. (2021) describe emotional dysregulation as a transdiagnostic construct across child and adolescent psychiatric disorders, while Lin et al. (2020) show that relationships between screen time and mood symptoms can be multicomponent rather than unitary. This makes mood dysregulation a useful outcome for examining digital behavior without reducing emotional functioning to a single diagnosis.

The relationship is also plausibly reciprocal. Su et al. (2026) reported bidirectional associations between adolescents’ negative emotional processes and problematic technology use. Zhou et al. (2026) linked emotion regulation and impulsivity to risk for self-injurious behavior, and Tu et al. (2025) demonstrated that cognitive reappraisal can influence craving and inhibitory control in adolescents with gaming disorder. These findings reinforce the importance of cautious, noncausal interpretation in cross-sectional studies.

### Integrated gap

Four gaps motivate the present analysis. First, much screen-time research does not model reward sensitivity directly. Second, reward-processing studies often use clinical or neuroimaging samples rather than everyday screen use. Third, studies of mood dysregulation and technology use frequently focus on problematic use or addiction rather than total daily screen time. Fourth, studies that combine self-reported motivational and affective constructs rarely test whether the scales are empirically distinguishable before interpreting their joint associations. The present study examines the three variables in one adolescent sample while explicitly separating hypothesized mechanisms from concurrent statistical evidence.

## Theoretical framework

The study is informed by an integrated developmental framework combining adolescent vulnerability, reward-sensitivity accounts, emotion-regulation theory, and interactional models of problematic media use. Adolescence is conceptualized as a period in which socioemotional and motivational systems may become highly responsive before regulatory-control capacities are fully mature. In that developmental context, screen-based environments can supply frequent, emotionally salient, and reward-laden cues (Orben et al., 2024; Chen et al., 2023).

Figure 1 distinguishes what the present study tested from what it did not. Solid lines denote the concurrent bivariate associations estimated in this study; dashed arrows denote temporal, reciprocal, or reinforcement processes proposed in prior longitudinal and theoretical work, which cross-sectional data cannot test. The figure also marks the measurement overlap between the reward-sensitivity and mood-dysregulation scales documented in the Results. Conceptually, screen-based environments are treated as a behavioral context, reward sensitivity as a candidate motivational susceptibility, and mood dysregulation as an affect-regulation outcome of interest. The empirical analysis tests concurrent associations, construct distinctiveness, and the statistical attenuation of the screen-time coefficient after reward sensitivity is added; it does not test temporal mediation, mechanisms, or causal feedback loops.

**Figure 1:**
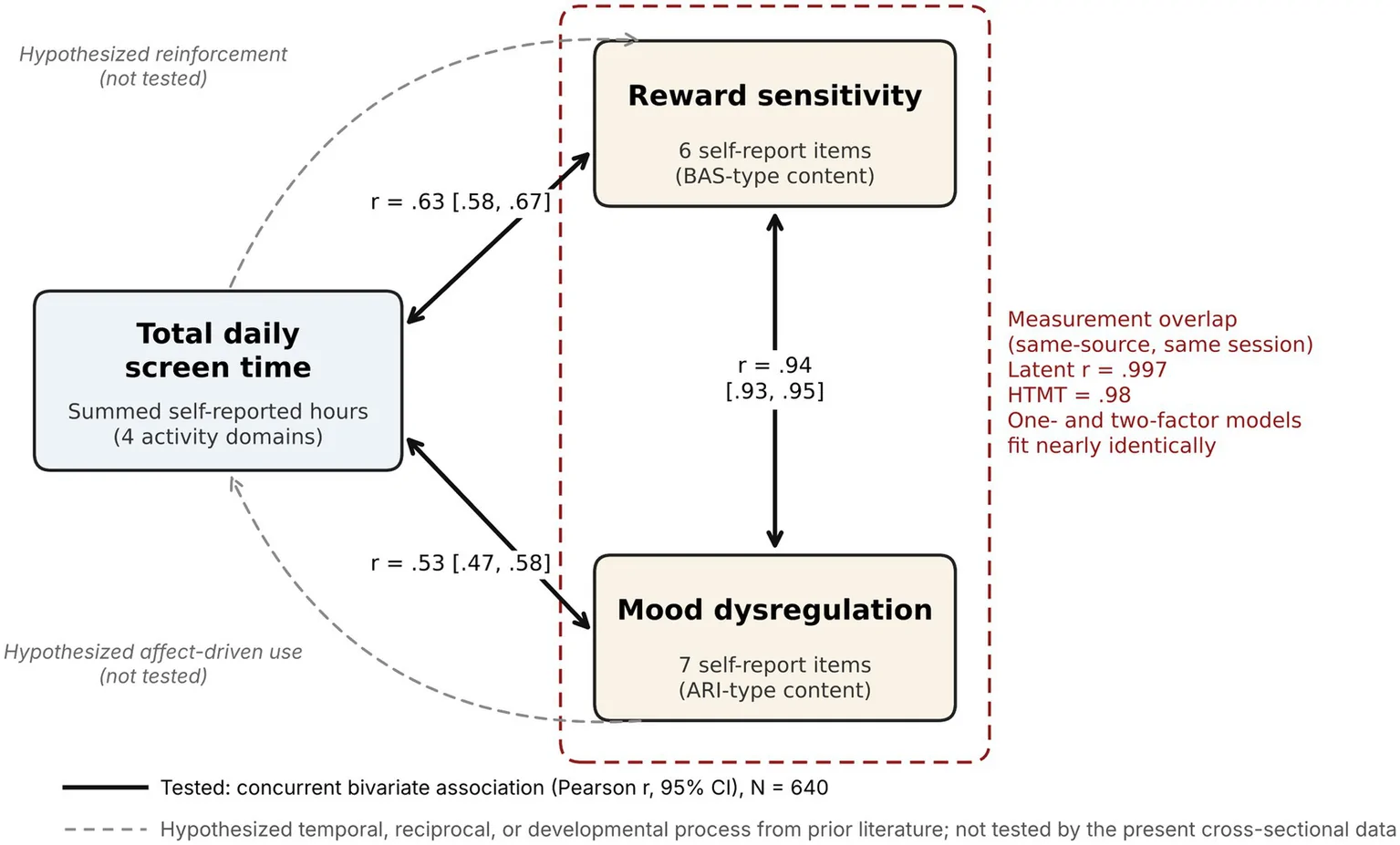
Conceptual framework distinguishing tested concurrent associations from hypothesized (untested) processes. Solid double-headed lines are concurrent Pearson correlations with 95% confidence intervals (*N* = 640). Dashed arrows are hypothesized processes from prior literature that the present cross-sectional data do not test. The dashed red outline marks the measurement overlap between the reward-sensitivity and mood-dysregulation scales (latent correlation and heterotrait–monotrait ratio from the measurement-model analyses; see Table 3). BAS, Behavioral Activation System; ARI, Affective Reactivity Index.

Within this framework, reward sensitivity captures individual differences in responsiveness to rewarding stimuli such as social approval, novelty, game-related incentives, and intermittent digital feedback. Mood dysregulation captures difficulty modulating negative affect and returning to emotional baseline. Interactional media-use models further suggest that dispositional vulnerabilities, environmental affordances, and reinforcing patterns of use may operate together rather than in isolation (Domoff et al., 2020). Consistent with the general objective stated in the Introduction, the study therefore estimates the concurrent associations among screen time, reward sensitivity, and mood dysregulation and evaluates whether the covariate-adjusted screen-time–mood-dysregulation association changes when reward sensitivity is taken into account, after first testing whether reward sensitivity and mood dysregulation are empirically distinct.

## Method

### Research design

A cross-sectional correlational design was used to examine concurrent relationships among screen time, reward sensitivity, and mood dysregulation in Egyptian adolescents. No participant condition was manipulated, and the design does not establish temporal ordering or causality. All descriptive, psychometric, bivariate, group-comparison, and regression statistics were computed from the final analytic dataset (Supplementary Data Sheet 1).

### Participants and sampling procedure

The final analytic sample comprised 640 Egyptian adolescents and emerging adults aged 13–19 years (M = 16.13, SD = 1.97). Participants were recruited through a multistage, non-probability convenience sampling strategy designed to capture variation across educational and sociodemographic contexts. Recruitment was conducted in three major Egyptian governorates—Cairo, Giza, and Alexandria—and encompassed eight public and private educational districts. Eighteen educational institutions were formally invited to participate, including 10 public schools, five private/international schools, and three university faculties. Fourteen institutions agreed to participate (eight public schools, four private/international schools, and two university faculties), corresponding to an institutional participation rate of 77.8%. Eligible institutions were required to be formally accredited or authorized to operate, enroll both male and female students, and provide administrative approval for the distribution of study materials.

Participant recruitment was conducted on site during the 2023–2024 academic year using classroom- and lecture-hall-based procedures. Study packages containing participant assent forms, parental informed-consent forms for participants younger than 18 years, and a paper-and-pencil questionnaire were distributed to 850 eligible students. A total of 712 consent/assent packages and questionnaires were returned, corresponding to an initial non-response or refusal rate of 16.2%. Before analysis, 72 returned questionnaires were excluded because of substantial missing data (>15% of responses missing; *n* = 48) or invalid response patterns (*n* = 24; 18 failed attention checks, four straightlining, two multiple responses) (*n* = 24). The resulting analytic sample therefore comprised *N* = 640 participants, representing a net completion rate of 75.3% relative to the 850 students initially invited to participate.

Because neither institutions nor individual participants were selected probabilistically, the sample should not be regarded as nationally representative of Egyptian adolescents and emerging adults. Accordingly, the term *diverse* refers only to the observed variation within the sample in educational setting, parental educational attainment, place of residence, and digital access. Gender was assessed using a binary self-report item with the response categories male and female; sex assigned at birth and gender identity were not assessed separately. The final sample included 322 males (50.3%) and 318 females (49.7%).

With respect to educational level, 160 participants (25.0%) were enrolled in middle school, 288 (45.0%) in high school, and 192 (30.0%) in the first or second year of undergraduate study. Age and educational level were fully nested in the present sample: all middle-school participants were aged 13–14 years, all high-school participants were aged 15–17 years, and all university participants were aged 18–19 years. Overall, 429 participants (67.0%) attended public institutions, whereas 211 (33.0%) attended private or international institutions. The highest level of parental education was basic or secondary education for 241 participants (37.7%), university education for 293 (45.8%), and postgraduate education for 106 (16.6%). Regarding place of residence, 423 participants (66.1%) were classified as residing in urban areas and 217 (33.9%) in semi-urban or rural areas.

The sample profile is presented in Table 1.

**Table 1:** Sample characteristics (*N* = 640).

Characteristic	*n*	%
Gender (binary self-report)		
Male	322	50.3
Female	318	49.7
Educational level (age range)		
Middle school (13–14 years)	160	25.0
High school (15–17 years)	288	45.0
University, early undergraduate years (18–19 years)	192	30.0
Age band		
Early/middle adolescence (13–16 years)	352	55.0
Late adolescence (17–19 years)	288	45.0
School/institution type		
Public	429	67.0
Private/international	211	33.0
Parental education		
Basic/secondary	241	37.7
University degree	293	45.8
Postgraduate degree	106	16.6
Residence		
Urban	423	66.1
Semi-urban/rural	217	33.9

Age: M = 16.13, SD = 1.97, range = 13–19 years. Age was fully nested within educational level. Parental education by school type: public, 55.2% basic/secondary, 41.7% university, 3.0% postgraduate; private/international, 1.9%, 54.0%, and 44.1%, respectively.

### Inclusion and exclusion criteria

Participants were eligible for inclusion if they were Egyptian nationals enrolled in a school or university, were aged 13–19 years, were able to complete the study measures in Arabic, and provided informed assent or consent. For participants younger than 18 years, written informed consent from a parent or legal guardian was additionally required. Operational exclusion criteria were specified prior to data collection. Substantial item nonresponse was defined as missing more than 15% of the total survey items (approximately >10 items across the complete survey battery). Invalid response patterns were defined as any of the following: (a) failure to respond correctly to embedded attention-check items (e.g., “Select ‘Strongly Disagree’ for this item”); (b) straightlining, operationalized as providing an identical response to every item within a multi-item subscale containing reverse-coded items; or (c) marking multiple responses on paper-and-pencil items for which only one response option was permitted.

A total of 850 eligible students were initially approached and provided with study packages. Of these, 712 returned the required consent or assent documentation together with completed paper-and-pencil questionnaires. Application of the pre-established exclusion criteria resulted in the removal of 72 respondents. Specifically, 48 questionnaires were excluded because more than 15% of survey responses were missing, 18 because participants failed one or more embedded attention checks, four because of straightlining across subscales containing reverse-coded items, and two because multiple responses had been marked on single-response items. Apparent out-of-range entries were verified against the original paper questionnaires. Where the intended response could be established unambiguously, the value was corrected during data entry; where it remained ambiguous, the response was treated according to the predefined exclusion rules. Consequently, no unresolved out-of-range values remained in the analytic dataset.

The final analytic dataset comprised 640 complete cases (*N* = 640), with no missing or unresolved out-of-range values for any variable included in the primary analyses. To assess the robustness of the findings to potentially careless responding beyond the predefined exclusion criteria, additional *post hoc* screening indices, including intra-individual response variability and longstring indices, were calculated and examined in sensitivity analyses, as described in the Data Analysis section.

### Measures

The instrument package comprised four sections: demographic information, daily screen time, reward sensitivity, and mood dysregulation. The complete item set, response formats, and scoring instructions are provided in Supplementary Data Sheet 1 (Section A). Throughout, “screen time” denotes the self-reported hours measure described below, whereas “screen use” refers to the behavior more generally.

#### Daily screen time

This author-constructed four-item assessment asked participants to “estimate the average number of hours you spend using a screen (smartphone, tablet, laptop, or TV) on a typical day” for each of four categories: social media (e.g., Instagram, TikTok, Facebook), gaming (online, console, or mobile), streaming/video (e.g., YouTube, Netflix, TV shows), and browsing/communication (e.g., web surfing, WhatsApp). The reference period was therefore a typical day; weekdays and weekend days were not distinguished. Responses were open numeric estimates in hours, so zero use could be reported; in practice, no participant reported zero hours in any category (minimum for gaming = 0.1 h; 15 participants reported less than 0.5 h of gaming and 23 less than 0.5 h of browsing/communication), so no special handling of zero values was required. Participants were not asked to apportion simultaneous activities (e.g., messaging while streaming video), so overlapping time could be counted in more than one category. Total daily screen time was calculated as the arithmetic sum of the four categories and is treated as a formative exposure index rather than a reflective scale (Bollen and Lennox, 1991); internal-consistency coefficients are therefore not reported for it. Because of possible double-counting, the total should be read as an upper-bound estimate of time spent across activities rather than as clock time.

#### Reward sensitivity

Reward sensitivity was assessed using six items measuring affective responsiveness to rewarding outcomes and approach toward novel or potentially rewarding experiences (e.g., “When I get something I want, I feel excited and energized” and “I am always looking for new and exciting things to experience”). The six items were adapted by the authors from two subscales of the original 20-item Behavioral Inhibition System/Behavioral Activation System (BIS/BAS) scales developed by Carver and White (1994): Reward Responsiveness (three adapted items) and Fun Seeking (three adapted items). The study did not administer the original subscales in their published form. Rather, the selected items were paraphrased to simplify sentence structure, enhance developmental comprehensibility across the 13–19-year age range, and facilitate culturally appropriate translation into Modern Standard Arabic.

This brief six-item operationalization was adopted for two main reasons. First, reducing the number of items minimized participant burden within a broader multi-construct survey battery administered in classroom and lecture-hall settings. Second, item selection was theoretically guided to represent two aspects of reward sensitivity considered particularly relevant to digital behavior: affective responsiveness to anticipated or obtained rewards and behavioral orientation toward novel and potentially rewarding experiences. The Drive subscale was not included because it primarily assesses persistent pursuit of desired goals and was therefore less closely aligned with the reward-reactivity construct examined in the present study. Because the resulting six-item measure constituted an adapted rather than a previously validated short form, it was treated as a study-specific operationalization of reward sensitivity rather than as the original BIS/BAS instrument.

The adapted items underwent a translation and back-translation procedure. Bilingual researchers translated the items into Modern Standard Arabic, after which the wording was reviewed for semantic clarity, developmental appropriateness, and cultural relevance. The translated items were also reviewed by a panel with expertise in developmental psychology to assess content relevance and conceptual equivalence with the intended reward-sensitivity domains.

All six items were rated on a 4-point Likert-type scale ranging from 1 (strongly disagree) to 4 (strongly agree). A composite reward-sensitivity score was calculated as the mean of the six item responses, yielding a possible score range of 1–4, with higher scores indicating greater reward sensitivity. Because this adapted six-item operationalization had not previously been validated for Arabic-speaking participants in the age range examined, its dimensional structure, internal consistency, and discriminant validity were evaluated empirically in the present sample and are reported in the Results section.

#### Mood dysregulation

Mood dysregulation was assessed using seven items addressing irritability, heightened negative affective reactivity, difficulty regulating emotions when distressed, and prolonged recovery from negative emotional states (e.g., “I am easily annoyed by others” and “When I am angry or upset, it takes me a long time to calm down”). Participants rated each item with reference to the preceding 6 months using a 3-point response scale ranging from 1 (not true) to 3 (certainly true). Four items (Items 1, 2, 4, and 5) were adapted from the self-report Affective Reactivity Index (ARI; Stringaris et al., 2012) to represent irritability and heightened reactivity to frustration or anger. Three additional items (Items 3, 6, and 7) were adapted from content represented in the Difficulties in Emotion Regulation Scale (DERS; Gratz and Roemer, 2004) to capture difficulties regulating emotional responses, maintaining behavioral control when distressed, and managing intense negative emotions. The original ARI and DERS were therefore not administered in their complete or published forms; rather, selected content from the two measures was adapted to construct a brief, study-specific measure of mood dysregulation.

The seven items were combined to provide a broader operationalization of mood dysregulation than would be captured by irritability alone. Whereas the ARI primarily assesses irritability and reactivity to anger or frustration, the selected DERS-derived content was included to represent difficulties modulating emotional responses once negative affect had been activated. The resulting measure was thus intended to capture two related components of mood dysregulation: heightened affective reactivity and impaired regulation of negative emotional states. This conceptual integration was considered particularly relevant to the present study because difficulties in both emotional reactivity and regulation may contribute to maladaptive behavioral responses during adolescence.

A composite mood-dysregulation score was calculated as the mean of the seven item responses, yielding a possible range of 1–3, with higher scores indicating greater mood dysregulation. Because this seven-item measure was an author-adapted composite drawing on selected content from two established instruments rather than a previously validated scale, it was treated as a study-specific operationalization of mood dysregulation. Its dimensional structure, internal consistency, and discriminant validity were therefore evaluated empirically in the present sample and are reported in the Results section.

### Instrument adaptation and pilot study

The reward-sensitivity and mood-dysregulation items were adapted for administration in Modern Standard Arabic using a structured cross-cultural adaptation procedure informed by Beaton et al. (2000). Two bilingual researchers independently translated the English items into Arabic. A third independent bilingual researcher then reviewed and synthesized the two forward translations into a single reconciled Arabic version, resolving minor differences in wording through discussion and comparison of conceptual meaning. Subsequently, two native English speakers who were fluent in Arabic and blinded to the original English wording independently back-translated the reconciled Arabic version into English. The research team compared the back-translated versions with the source items to identify potential discrepancies and to evaluate semantic, conceptual, and idiomatic equivalence.

The resulting Arabic items were then evaluated for content and face validity within the Egyptian context by a five-member expert panel comprising three developmental or clinical psychologists, one psychometrician, and one professor of educational sociology. Experts independently rated each item for clarity, developmental appropriateness for participants aged 13–19 years, and conceptual equivalence with the intended construct using a 4-point ordinal rating scale ranging from 1 (not relevant/clear) to 4 (highly relevant/clear). Quantitative evidence of content validity was examined using the Item-Level Content Validity Index (I-CVI) and a scale-level Content Validity Index. Across the 13 items constituting the two adapted measures, I-CVI values ranged from 0.80 to 1.00, indicating generally strong expert agreement regarding item relevance and clarity. Minor wording adjustments recommended by the panel were incorporated before pilot administration.

The revised Arabic items were subsequently pilot-tested with 30 Egyptian adolescents (M age = 15.8 years; 50% female) recruited from a secondary school that did not participate in the main study. The pilot was designed to evaluate item comprehensibility, readability, response-option formatting, and the practical burden of questionnaire administration rather than to establish psychometric properties. Participant feedback resulted in minor wording refinements to two reward-sensitivity items to improve sentence flow and comprehensibility. Completion of the full questionnaire battery required approximately 15–20 min. Given the limited pilot sample size (*n* = 30), factor analyses and formal reliability estimates were not conducted at this stage. Because the reward-sensitivity and mood-dysregulation measures were author-adapted brief measures without prior validation in this specific population, their dimensional structure, internal consistency, and discriminant validity were evaluated using the main analytic sample (*N* = 640), as reported in the Results section.

### Data collection and ethical considerations

Data were collected during the 2023–2024 academic year. The Social and Human Sciences Research Ethics Committee (ARTBSU-SHS-REC), Faculty of Arts, Beni-Suef University, subsequently granted retrospective ethics approval on 25 February 2026 (Approval No. ARTBSU-SHS-REC/SOC-11/1/2026/RANYA F; stated expiry 12 January 2027). The approval did not precede recruitment or data collection. According to the study records, prospective committee review was unavailable during collection because the university’s preceding research ethics committee had ceased operations and a successor committee was being established. Following that institutional transition, the protocol, consent and assent procedures, survey materials, and data-handling documentation were submitted for retrospective review. Administrative permission from the participating educational institutions was obtained before survey administration. Retrospective committee approval should not be construed as prospective ethical clearance. The study procedures were reported to follow relevant institutional requirements and the principles of the Declaration of Helsinki. Paper-and-pencil questionnaires were administered in classroom settings to middle- and high-school participants and in lecture halls to university participants. Trained research assistants provided standardized procedural instructions and supervised the sessions.

Participation was voluntary. Before enrollment, participants received information describing the purpose and procedures of the study, the voluntary nature of participation, confidentiality protections, and their right to decline participation or withdraw without penalty or adverse consequences for their academic standing. Consent procedures were determined according to participant age. For participants younger than 18 years, written informed consent was obtained from a parent or legal guardian in addition to written assent from the participant. Participants aged 18–19 years provided their own written informed consent and were not required to obtain parental or guardian consent, consistent with the applicable institutional requirements governing participation by legal adults.

Questionnaire responses were collected anonymously using de-identified paper survey packages labeled only with pre-assigned numeric identification codes. No directly identifying information, including names or national identification numbers, was collected or linked to the analytic dataset. Because the questionnaire included items addressing mood and emotional distress, all participants received a debriefing sheet after survey completion containing contact information for appropriate university counseling services and community-based mental health resources.

### Data analysis

#### Sample size and power

No formal *a priori* statistical power analysis was conducted before data collection. The target sample size was instead determined on the basis of recruitment feasibility and commonly used practical considerations for factor-analytic and multivariable modeling. Recruitment planning sought to obtain a sample substantially larger than the minimum required for stable estimation of the adapted measurement models, using an approximate target of at least 20 participants per item for scale evaluation and an overall target of at least 500 participants to support multivariable analyses and planned subgroup comparisons. Recruitment was intentionally extended beyond this target to accommodate anticipated non-response, incomplete questionnaires, and exclusions resulting from the predefined response-quality criteria.

To characterize the magnitude of effects that could be detected with the achieved sample, a sensitivity power analysis was conducted using G*Power version 3.1. With a two-sided significance level of *α* = 0.05 and statistical power of 1 − *β* = 0.80, the final analytic sample of *N* = 640 provided sensitivity to detect a Pearson correlation of approximately *r* = 0.11. For independent two-group comparisons, the minimum detectable standardized mean difference was approximately d = 0.22 for comparisons by gender (male: *n* = 322; female: *n* = 318) and *d* = 0.24 for comparisons by institution type (public: *n* = 429; private/international: *n* = 211). For hierarchical multiple regression, assuming nine covariate or control predictors and testing the incremental contribution of one additional predictor, the sample provided sensitivity to detect an incremental effect of approximately *f*^2^ = 0.012. According to Cohen's (1988) conventional benchmarks, these values fall within the small-effect range. The sensitivity analysis is reported to characterize the inferential resolution of the achieved sample and was not used as a substitute for the absence of an *a priori* power calculation.

#### Data screening

All records were checked for missing and out-of-range values and for duplicate records. In addition, three *post hoc* careless-responding indicators were computed (Meade and Craig, 2012): (a) identical responses to all items of both the reward-sensitivity and mood-dysregulation scales (long-string responding), (b) multivariate outliers on the 13 items (Mahalanobis distance, *p* < 0.001), and (c) duplicate records. These indicators were used to define sensitivity subsamples, not to exclude cases from the primary analyses.

#### Measurement models and discriminant validity

To test whether reward sensitivity and mood dysregulation are empirically distinguishable, a two-factor confirmatory factor analysis (CFA) model, in which the six reward-sensitivity and seven mood-dysregulation items loaded on separate correlated factors, was compared with a one-factor model in which all 13 items loaded on a single factor. Because the items had three or four response categories, the primary models treated them as ordered categorical and used the WLSMV estimator; as a robustness check, the models were re-estimated treating items as continuous with robust maximum likelihood (MLR; Rhemtulla et al., 2012). Fit was evaluated with the CFI, TLI, RMSEA, and SRMR (Hu and Bentler, 1999), and nested models were compared with scaled *χ*^2^ difference tests and, for MLR, with AIC and BIC. Discriminant validity was assessed with the estimated latent correlation and its 95% CI, the heterotrait–monotrait ratio (HTMT; values above 0.85–0.90 indicate inadequate discrimination; Henseler et al., 2015), and the Fornell–Larcker criterion (Fornell and Larcker, 1981; Rönkkö and Cho, 2022). Exploratory two-factor solutions with oblique rotation (an ESEM-type check of cross-loadings; Asparouhov and Muthén, 2009) and parallel analysis (Horn, 1965) were used to examine dimensionality. Reliability was indexed by Cronbach’s *α* and McDonald’s *ω* total (McNeish, 2018). Item wording was inspected for semantic redundancy across scales, and the proportion of item variance captured by a single unrotated component (Harman’s single-factor test) was computed as a coarse indicator of common-method variance, recognizing its limited sensitivity (Podsakoff et al., 2012).

#### Descriptive, bivariate, and group analyses

Pearson correlations were computed with Fisher-*z* 95% CIs, and effect sizes were described using Cohen’s (1988) conventional benchmarks (*r* = 0.10, 0.30, and 0.50 for small, medium, and large associations; *d* = 0.20, 0.50, and 0.80). Gender and school-type comparisons used Welch’s *t* test, with Cohen’s *d* (pooled SD) and its 95% CI as the effect-size index. Educational-level differences were tested with one-way ANOVA (with Welch’s *F* as a check) and *η*^2^ with a 95% CI derived from the noncentral *F* distribution; significant omnibus tests were followed by Games–Howell *post hoc* comparisons (Games and Howell, 1976). Correlations were also computed separately in early/middle (13–16 years) and late (17–19 years) adolescence and compared with Fisher’s *z* test.

#### Hierarchical regression

The hierarchical regression was framed strictly as an analysis of conditional associations, not as an explanatory decomposition of variance. Step 1 entered age, gender, educational level, school/institution type, parental education, and residence. These covariates were entered first because they are temporally prior, exogenous characteristics that plausibly relate to both screen use and emotional functioning and therefore need to be held constant before the focal associations are estimated. Step 2 added total screen time, establishing its adjusted association with mood dysregulation before reward sensitivity was entered. Step 3 added reward sensitivity, allowing the screen-time coefficient to be compared before and after reward sensitivity was included. Because Δ*R*^2^ depends on entry order, Steps 2 and 3 were also reversed to estimate the unique increment of screen time beyond reward sensitivity. Unstandardized coefficients (*B*) are reported with heteroskedasticity-consistent (HC3) standard errors and 95% CIs (Long and Ervin, 2000), and standardized coefficients (*β*) are reported for continuous predictors. Percentile bootstrap 95% CIs (2,000 resamples) were computed for *R*^2^ and Δ*R*^2^.

#### Regression diagnostics and sensitivity analyses

Regression assumptions and model diagnostics were evaluated before interpretation of the primary regression results. Residual distributional characteristics were examined using skewness, kurtosis, and the Shapiro–Wilk test. Homoskedasticity was assessed using the studentized Breusch–Pagan test, whereas linearity and potential functional-form misspecification were examined using the Ramsey RESET test and models incorporating quadratic polynomial terms. Residual independence was evaluated using the Durbin–Watson statistic. Potentially influential observations were identified using Cook’s distance, leverage values, and studentized residuals, and multicollinearity was evaluated using variance inflation factors (VIFs). Because age and educational tier were fully nested in the present sample—for example, all participants aged 18–19 years were early-undergraduate students—the preliminary model that simultaneously included both variables exhibited substantial multicollinearity, with the VIF for age reaching 15.51. Accordingly, a sensitivity specification retained educational tier and omitted age; this reduced all VIFs to 4.53 or below (see Results).

A series of sensitivity analyses was conducted to evaluate the robustness of the primary findings to alternative data-screening and model-specification decisions. The regression models were re-estimated under five additional specifications: (a) after excluding potentially influential observations exceeding the conventional Cook’s distance criterion of *D* > 4/*N*; (b) after excluding cases identified by the *post hoc* careless-responding screens based on intra-individual response variability and longstring indices; (c) using a parsimonious covariate specification containing only gender and age; (d) separately within two age bands (13–16 and 17–19 years); and (e) replacing the aggregate screen-time measure with its four domain-specific component measures. Consistency in the direction, magnitude, and inferential pattern of the focal associations across these alternative specifications was used to evaluate the robustness of the primary results.

Potential dependence arising from the recruitment of participants within the same educational institutions was also considered. To maximize participant anonymity, institution- and classroom-level identifiers were not recorded on the survey instruments or retained in the analytic dataset. Consequently, individual observations could not be linked to specific institutions or classrooms in the final dataset (*N* = 640), precluding estimation of multilevel models and the calculation of institution- or classroom-clustered robust standard errors. Institution type (public vs. private/international) and residence were therefore included as fixed-effect covariates in the primary adjusted models to account for measured contextual variation across broad educational settings. These covariates, however, cannot fully account for residual within-institution dependence. The inability to model institution- or classroom-level clustering directly was therefore considered when interpreting the precision of the regression estimates and is acknowledged as a limitation of the study.

Statistical analyses were conducted in R version 4.3.3 (R Core Team, 2024). Measurement and structural models were estimated using the lavaan package (Version 0.6–17; Rosseel, 2012) together with semTools (Version 0.5–6). Additional psychometric analyses and regression diagnostics were conducted using psych (Version 2.4.1), car (Version 3.1–2), lmtest (Version 0.9–40), and sandwich (Version 3.1–0). All inferential tests were two-sided, with statistical significance evaluated at *α* = 0.05. The complete analysis script and the data necessary to reproduce the reported analyses are provided in the Supplementary material.

## Results

### Data screening and sample structure

No item values were missing or out of range. Four analytic profiles were identical to another record across all retained variables. This finding alone does not establish repeat submissions; the records were retained in the primary analysis because their provenance could not be verified from the available de-identified records. Long-string responding was common: 268 participants (41.9%) gave identical responses to all six reward-sensitivity items, 243 (38.0%) to all seven mood-dysregulation items, and 84 (13.1%) to all 13 items of both scales; 36 cases (5.6%) were multivariate outliers. Excluding the 122 cases flagged by at least one of the three screens left a sensitivity subsample of *n* = 518 (Table 2). Age and educational level were fully nested (Table 1). School type was strongly associated with parental education, *χ*^2^(2) = 255.44, *p* < 0.001, Cramér’s *V* = 0.63: 44.1% of private/international-school participants but only 3.0% of public-school participants had a parent with a postgraduate degree. Private/international-school participants were also more often urban (93.8% vs. 52.4%).

**Table 2:** Sensitivity analyses for the focal coefficients.

Specification	*n*	Screen time, Step 2: *B* [95% CI], *p*	Screen time, Step 3: *B* [95% CI], *p*	Reward sensitivity, Step 3: *B* [95% CI]	*R*^2^ (Step 3)
Primary model	640	0.035 [0.024, 0.047], <0.001	−0.004 [−0.010, 0.001], 0.135	0.850 [0.816, 0.884]	0.935
Age omitted	640	0.036 [0.024, 0.047], <0.001	−0.004 [−0.010, 0.002], 0.153	0.853 [0.819, 0.888]	0.933
Cook’s *D* > 4/*N* excluded	628	0.033 [0.022, 0.045], <0.001	−0.004 [−0.009, 0.001], 0.158	0.852 [0.821, 0.882]	0.943
Careless-responding screens excluded	518	0.034 [0.021, 0.047], <0.001	−0.002 [−0.009, 0.004], 0.436	0.828 [0.793, 0.864]	0.917
No long-string responding on either scale	213	0.015 [−0.002, 0.032], 0.078	−0.004 [−0.014, 0.006], 0.479	0.755 [0.612, 0.899]	0.925
Minimal covariates (gender, age)	640	0.095 [0.086, 0.104], <0.001	−0.008 [−0.013, −0.003], < 0.001	0.821 [0.799, 0.843]	0.921
Ages 13–16	352	0.042 [0.026, 0.057], <0.001	0.001 [−0.007, 0.009], 0.766	0.845 [0.804, 0.886]	0.938
Ages 17–19	288	0.029 [0.012, 0.047], 0.001	−0.011 [−0.020, −0.002], 0.019	0.849 [0.791, 0.907]	0.939

All estimates use HC3 standard errors. Step 2 adds total screen time to the covariates; Step 3 adds reward sensitivity. Age-band models omit the educational-level indicators, which are nested within age. Careless-responding screens: identical responses to all items of both psychological scales, multivariate outliers (Mahalanobis *p* < 0.001), or duplicate records.

### Measurement model and discriminant validity

The reward-sensitivity and mood-dysregulation scales could not be empirically distinguished (Table 3). With ordinal (WLSMV) estimation, the two-factor and one-factor models showed virtually identical fit. The scaled *χ*^2^ difference test favored the two-factor model, Δ*χ*^2^(1) = 19.19, *p* < 0.001, but this test is highly powered with *N* = 640, and the estimated latent correlation between the two factors was 0.997, 95% CI [0.996, 0.999]. The ordinal solution was also improper: several standardized loadings were at or above 1.0, and the polychoric correlation matrix was not positive definite because of sparse response categories. When items were treated as continuous (MLR), both models fitted poorly (CFI = 0.748, RMSEA ≈ 0.31), the one-factor model was not significantly worse, Δ*χ*^2^(1) = 1.34, *p* = 0.247, the information criteria barely differed (ΔAIC = 6.0 and ΔBIC = 1.5 in favor of the two-factor model), and the latent correlation was 0.998 [0.994, 1.002], with a CI that included 1.0. The HTMT ratio was 0.98, well above the 0.85–0.90 thresholds, and the squared latent correlation (0.99) exceeded the average variance extracted of both factors (0.96), so the Fornell–Larcker criterion was not met. Parallel analysis retained a single component (first eigenvalue = 11.61; second = 0.45), one unrotated component accounted for 80.3% of the variance in the 13 items, and exploratory two-factor solutions did not converge to an admissible solution (ultra-Heywood cases).

**Table 3:** Measurement model comparison and discriminant-validity indices for the reward-sensitivity and mood-dysregulation items.

Estimator/model	*χ*^2^ (df)	CFI	TLI	RMSEA [90% CI]	SRMR	Latent *r* [95% CI]
WLSMV (ordinal items)						
Two-factor	577.58 (64)	1.000	1.000	0.112 [0.104, 0.121]	0.061	0.997 [0.996, 0.999]
One-factor	587.12 (65)	1.000	1.000	0.112 [0.104, 0.121]	0.061	—
MLR (continuous items)						
Two-factor	2030.12 (64)	0.748	0.693	0.316 [0.304, 0.328]	0.058	0.998 [0.994, 1.002]
One-factor	1977.08 (65)	0.748	0.698	0.314 [0.302, 0.325]	0.058	—

*N* = 640. *χ*^2^ values are scaled test statistics and are not directly comparable across nested models; nested comparisons used scaled difference tests: WLSMV, Δ*χ*^2^(1) = 19.19, *p* < 0.001; MLR, Δ*χ*^2^(1) = 1.34, *p* = 0.247 (MLR two-factor vs. one-factor: AIC = 6364.0 vs. 6370.0; BIC = 6484.5 vs. 6486.0). The WLSMV two-factor solution contained standardized loadings ≥1.0 (range 0.915–1.002). Heterotrait–monotrait ratio (HTMT) = 0.98. Average variance extracted: reward sensitivity = 0.96, mood dysregulation = 0.96; squared latent correlation = 0.99. Reliability: reward sensitivity *α* = 0.96, *ω* = 0.96; mood dysregulation *α* = 0.96, *ω* = 0.96; all 13 items *α* = 0.98, *ω* = 0.98. Parallel analysis retained one component.

Item-level inspection indicated that the overlap was not attributable to semantically duplicated items: the reward-sensitivity items describe excitement and approach toward rewards, whereas the mood-dysregulation items describe irritability and difficulty calming down. Nevertheless, cross-scale item correlations (*r* = 0.59–0.99, M = 0.78) were as high as within-scale correlations (reward sensitivity: 0.59–0.95, M = 0.81; mood dysregulation: 0.51–0.98, M = 0.77). For example, reward-sensitivity item 1 (“When I get something I want, I feel excited and energized”) correlated r = 0.99 with mood-dysregulation item 6 (“I find it difficult to control my reactions when I am frustrated”). Near-identity between semantically distinct items is more consistent with a shared response process, such as a general intensity or acquiescent response style, common-method variance, or a data-processing artefact, than with substantive construct overlap, but the present data cannot discriminate among these explanations. Internal consistency was high for each scale (reward sensitivity: *α* = 0.96, *ω* = 0.96; mood dysregulation: *α* = 0.96, *ω* = 0.96) and equally high when all 13 items were combined (*α* = 0.98, *ω* = 0.98), which illustrates that high reliability does not establish that two scales measure distinct constructs.

Accordingly, H5 was not supported: the two-factor structure required to treat reward sensitivity as a predictor distinct from the outcome could not be established. The regression analyses below are retained because they address the study’s original research question, but they are interpreted as quantifying the consequences of this overlap rather than as evidence that reward sensitivity explains or statistically accounts for a separate construct of mood dysregulation.

### Descriptive statistics and internal consistency

Reward sensitivity and mood dysregulation both had high internal consistency (*α* = 0.96, *ω* = 0.96 for each; Table 4). Participants reported a mean summed screen time of 9.96 h per day (SD = 3.76; range 2.5–20.1), a mean reward sensitivity score of 3.05 (SD = 0.74), and a mean mood dysregulation score of 2.07 (SD = 0.62). Because activity categories could overlap, the screen-time total should be read as an upper-bound estimate: 44 participants (6.9%) reported totals above 16 h per day and 17 (2.7%) above 18 h, values that are implausible as clock time. Screen time is therefore interpreted in relative terms (more versus less reported use) rather than against any normative threshold. The domain estimates were very strongly intercorrelated (social media–streaming *r* = 0.96, streaming–browsing *r* = 0.97, social media–browsing *r* = 0.87, gaming with the other domains *r* = 0.54), suggesting that respondents may have scaled their estimates across categories rather than reporting independent activities.

**Table 4:** Descriptive statistics and internal-consistency reliability coefficients for the main study variables.

Variable	M	SD	Min	Max	Skewness	Kurtosis	*α*	*ω*
Age	16.13	1.97	13.00	19.00	−0.08	−1.19	—	—
Social media screen time	3.87	1.28	1.10	7.40	0.34	−0.58	—	—
Gaming screen time	2.58	1.46	0.10	6.60	0.80	−0.03	—	—
Streaming/video screen time	2.18	0.86	0.50	4.80	0.49	−0.29	—	—
Browsing/communication screen time	1.33	0.68	0.30	3.60	0.80	0.08	—	—
Total daily screen time	9.96	3.76	2.50	20.10	0.42	−0.48	—	—
Reward sensitivity	3.05	0.74	1.67	4.00	−0.05	−1.52	0.96	0.96
Mood dysregulation	2.07	0.62	1.00	3.00	−0.03	−1.13	0.96	0.96

*N* = 640. Screen-time values are self-reported hours on a typical day; no participant reported zero hours in any category. Internal-consistency coefficients are not reported for screen time, which is a formative exposure index formed by summing heterogeneous activity categories. *α* = Cronbach’s alpha; *ω* = McDonald’s omega total. Kurtosis is excess kurtosis.

### Bivariate associations

Total daily screen time correlated *r* = 0.53, 95% CI [0.47, 0.58], with mood dysregulation and *r* = 0.63 [0.58, 0.67] with reward sensitivity (both *p* < 0.001; Table 5). By Cohen’s (1988) conventional benchmarks, both associations are large (*r* ≥ 0.50). Reward sensitivity and mood dysregulation correlated *r* = 0.94 [0.93, 0.95], *p* < 0.001, corresponding to approximately 89% shared variance at the scale-score level; together with the measurement-model results, this indicates that the two scale scores are not empirically separable in this dataset. Age correlated weakly with screen time [*r* = 0.13 (0.05, 0.20)] and reward sensitivity [*r* = 0.15 (0.08, 0.23)] and was not associated with mood dysregulation [*r* = 0.05 (−0.03, 0.12)]. At the domain level, correlations with mood dysregulation varied considerably (social media *r* = 0.72, streaming/video *r* = 0.57, browsing/communication *r* = 0.40, gaming *r* = 0.22; with reward sensitivity, *r* = 0.79, 0.63, 0.45, and 0.34, respectively), so the total-hours index aggregates components with quite different associations.

**Table 5:** Pearson correlations among the main variables with 95% confidence intervals.

Variable	1	2	3	4
1. Age	—			
2. Total screen time	0.13^**^ [0.05, 0.20]	—		
3. Reward sensitivity	0.15^***^ [0.08, 0.23]	0.63^***^ [0.58, 0.67]	—	
4. Mood dysregulation	0.05 [−0.03, 0.12]	0.53^***^ [0.47, 0.58]	0.94^***^ [0.93, 0.95]	—

*N* = 640. Values in brackets are 95% confidence intervals (Fisher *z*). ^**^p < 0.01. ^***^p < 0.001.

The associations of screen time with mood dysregulation and with reward sensitivity were stronger in late adolescence (17–19 years, *n* = 288; *r* = 0.63 and 0.74) than in early/middle adolescence (13–16 years, *n* = 352; *r* = 0.39 and 0.47), Fisher’s *z* = −4.15 and −5.63, both *p* < 0.001, whereas the reward sensitivity–mood dysregulation correlation was similar in the two bands (0.94 and 0.95). Mean screen time (10.74 vs. 9.33 h), reward sensitivity (3.29 vs. 2.85), and mood dysregulation (2.22 vs. 1.95) were also higher in the older band.

### Group differences

*Gender.* Males reported more screen time than females (10.42 vs. 9.50 h), *t*(638.0) = 3.14, *p* = 0.002, *d* = 0.25 [0.09, 0.40], a small effect. Females scored higher on reward sensitivity, *t*(636.4) = −4.05, *p* < 0.001, *d* = −0.32 [−0.48, −0.16] (small), and on mood dysregulation, *t*(627.3) = −7.62, *p* < 0.001, *d* = −0.60 [−0.76, −0.44] (medium). *School/institution type.* Participants in private/international institutions scored higher than those in public institutions on all three variables, with large effects: screen time, *d* = −1.36 [−1.54, −1.18]; reward sensitivity, *d* = −2.22 [−2.43, −2.02]; and mood dysregulation, *d* = −1.83 [−2.02, −1.63] (all *p* < 0.001). These differences were largely confounded with parental education: within the basic/secondary and postgraduate parental-education strata, public- and private-school participants did not differ in mood dysregulation (1.58 vs. 1.68, *p* = 0.63, private *n* = 4; 2.92 vs. 2.92, *p* = 0.97, public *n* = 13), whereas a difference remained within the university stratum (1.98 vs. 2.47, *p* < 0.001). These contrasts should be interpreted descriptively because school type may proxy multiple unmeasured social and economic differences. *Educational level.* Screen time differed by educational level, *F*(2, 637) = 68.22, *p* < 0.001, *η*^2^ = 0.18 [0.13, 0.23]; Welch’s *F*(2, 384.3) = 65.83, *p* < 0.001. Games–Howell comparisons showed that high-school students reported more screen time than middle-school students (mean difference = 3.30 h [2.49, 4.11], *p* < 0.001, *d* = 0.92) and university students [3.07 h (2.34, 3.79), *p* < 0.001, *d* = 0.89], whereas middle-school and university students did not differ [−0.23 h (−1.04, 0.57), *p* = 0.78, *d* = −0.07]. Educational level was not significantly associated with reward sensitivity, *F*(2, 637) = 0.14, *p* = 0.873, *η*^2^ < 0.001, or mood dysregulation, *F*(2, 637) = 2.63, *p* = 0.073, *η*^2^ = 0.01 [0.00, 0.03]. In sum, the gender differences were statistically significant with small-to-medium effects; the school-type differences were large but substantially confounded with parental education; and educational level differentiated only screen time, a difference driven by the high-school group (see Table 6).

**Table 6:** Group differences by gender, school type, and educational level.

Comparison	Outcome	Group means	Test	*p*	Effect size [95% CI]
Gender	Total screen time	Male = 10.42; Female = 9.50	*t*(638.0) = 3.14	0.002	*d* = 0.25 [0.09, 0.40]
Gender	Reward sensitivity	Male = 2.93; Female = 3.16	*t*(636.4) = −4.05	<0.001	*d* = −0.32 [−0.48, −0.16]
Gender	Mood dysregulation	Male = 1.89; Female = 2.25	*t*(627.3) = −7.62	<0.001	*d* = −0.60 [−0.76, −0.44]
School type	Total screen time	Public = 8.54; Private = 12.86	*t*(371.9) = −15.47	<0.001	*d* = −1.36 [−1.54, −1.18]
School type	Reward sensitivity	Public = 2.67; Private = 3.81	*t*(632.9) = −31.97	<0.001	*d* = −2.22 [−2.43, −2.02]
School type	Mood dysregulation	Public = 1.79; Private = 2.65	*t*(518.3) = −23.56	<0.001	*d* = −1.83 [−2.02, −1.63]
Education level	Total screen time	Middle = 8.41; High = 11.71; University = 8.64	*F*(2, 637) = 68.22	<0.001	*η*^2^ = 0.18 [0.13, 0.23]
*Post hoc*	High − Middle	Δ = 3.30 [2.49, 4.11]	Games–Howell	<0.001	*d* = 0.92
*Post hoc*	High − University	Δ = 3.07 [2.34, 3.79]	Games–Howell	<0.001	*d* = 0.89
*Post hoc*	Middle − University	Δ = −0.23 [−1.04, 0.57]	Games–Howell	0.776	*d* = −0.07
Education level	Reward sensitivity	Middle = 3.04; High = 3.06; University = 3.03	*F*(2, 637) = 0.14	0.873	*η*^2^ < 0.001 [0.00, 0.01]
Education level	Mood dysregulation	Middle = 2.17; High = 2.05; University = 2.03	*F*(2, 637) = 2.63	0.073	*η*^2^ = 0.01 [0.00, 0.03]

*t* tests are Welch tests (degrees of freedom adjusted for unequal variances). *d* uses the pooled SD; negative values indicate higher scores for females or for private/international institutions. *η*^2^ confidence intervals are based on the noncentral *F* distribution. *Post hoc* differences are in hours with 95% simultaneous CIs.

### Hierarchical multiple regression

Step 1 (demographic and contextual covariates) explained 69.5% of the variance in mood dysregulation, *R*^2^ = 0.695, bootstrap 95% CI [0.665, 0.731], adjusted *R*^2^ = 0.692, *F*(8, 631) = 180.09, *p* < 0.001 (Table 7). This very high proportion reflects mainly parental education [postgraduate vs. basic/secondary: *B* = 0.950 (0.838, 1.063); university vs. basic/secondary: *B* = 0.505 (0.420, 0.590)] and school type, two strongly associated variables. Covariates also explained large proportions of the variance in reward sensitivity (*R*^2^ = 0.758) and screen time (*R*^2^ = 0.615). Such magnitudes are unusual for demographic variables and may reflect genuine contextual inequalities, the non-probability selection of institutions, unmodelled clustering within institutions, or response patterns that differ by recruitment site; the present data cannot distinguish among these explanations, and the covariate coefficients are therefore not interpreted substantively. Adding total screen time at Step 2 increased explained variance to *R*^2^ = 0.713, Δ*R*^2^ = 0.017 [0.007, 0.030], Δ*F*(1, 630) = 38.30, *p* < 0.001. At this step, screen time was positively associated with mood dysregulation, *B* = 0.035, HC3 SE = 0.006, 95% CI [0.024, 0.047], *β* = 0.21, *p* < 0.001.

**Table 7:** Three-step hierarchical regression of mood dysregulation on covariates, screen time, and reward sensitivity.

Step	Predictor	*B*	SE (HC3)	95% CI (HC3)	*β*	*p*
1	Intercept	3.095	0.450	[2.210, 3.979]		<0.001
1	Male	−0.160	0.038	[−0.235, −0.085]		<0.001
1	Middle school	0.028	0.081	[−0.130, 0.187]		0.726
1	University	0.180	0.075	[0.033, 0.327]		0.017
1	Public school	−0.387	0.043	[−0.471, −0.303]		<0.001
1	Parent education: postgraduate	0.950	0.057	[0.838, 1.063]		<0.001
1	Parent education: university	0.505	0.043	[0.420, 0.590]		<0.001
1	Urban residence	0.206	0.045	[0.117, 0.295]		<0.001
1	Age	−0.079	0.029	[−0.136, −0.022]	−0.25	0.007
2	Intercept	2.680	0.457	[1.782, 3.578]		<0.001
2	Male	−0.226	0.040	[−0.304, −0.148]		<0.001
2	Middle school	0.142	0.085	[−0.024, 0.308]		0.093
2	University	0.276	0.074	[0.131, 0.421]		<0.001
2	Public school	−0.320	0.041	[−0.401, −0.240]		<0.001
2	Parent education: postgraduate	0.779	0.064	[0.652, 0.905]		<0.001
2	Parent education: university	0.429	0.044	[0.342, 0.516]		<0.001
2	Urban residence	0.175	0.044	[0.089, 0.261]		<0.001
2	Age	−0.074	0.029	[−0.130, −0.017]	−0.23	0.010
2	Total screen time	0.035	0.006	[0.024, 0.047]	0.21	<0.001
3	Intercept	0.277	0.227	[−0.168, 0.722]		0.222
3	Male	−0.117	0.019	[−0.153, −0.080]		<0.001
3	Middle school	−0.007	0.040	[−0.087, 0.072]		0.855
3	University	0.119	0.032	[0.056, 0.182]		<0.001
3	Public school	0.066	0.020	[0.026, 0.106]		0.001
3	Parent education: postgraduate	0.046	0.031	[−0.014, 0.107]		0.132
3	Parent education: university	−0.110	0.021	[−0.151, −0.068]		<0.001
3	Urban residence	0.033	0.021	[−0.008, 0.074]		0.118
3	Age	−0.047	0.014	[−0.074, −0.020]	−0.15	<0.001
3	Total screen time	−0.004	0.003	[−0.010, 0.001]	−0.03	0.135
3	Reward sensitivity	0.850	0.017	[0.816, 0.884]	1.00	<0.001

*N* = 640. Reference groups: female, high school, private/international school, basic/secondary parental education, and semi-urban/rural residence. HC3 = heteroskedasticity-consistent standard errors. *β* is reported for continuous predictors (all continuous variables standardized). Step 1: *R*^2^ = 0.695 [0.665, 0.731], adjusted *R*^2^ = 0.692. Step 2: *R*^2^ = 0.713, Δ*R*^2^ = 0.017 [0.007, 0.030], Δ*F*(1, 630) = 38.30, *p* < 0.001. Step 3: *R*^2^ = 0.935, adjusted *R*^2^ = 0.933, Δ*R*^2^ = 0.222 [0.191, 0.251], Δ*F*(1, 629) = 2129.60, *p* < 0.001. Reversed order: reward sensitivity after covariates, Δ*R*^2^ = 0.239 [0.207, 0.269]; screen time after reward sensitivity, Δ*R*^2^ = 0.0002 [0.000, 0.001], Δ*F*(1, 629) = 2.17, *p* = 0.141. Bracketed values for *R*^2^ and Δ*R*^2^ are percentile bootstrap 95% CIs (2,000 resamples). Conventional OLS standard errors yielded the same inferences.

Adding reward sensitivity at Step 3 increased *R*^2^ to 0.935 (adjusted *R*^2^ = 0.933), Δ*R*^2^ = 0.222 [0.191, 0.251], Δ*F*(1, 629) = 2129.60, *p* < 0.001. Reward sensitivity had a coefficient of *B* = 0.850 [0.816, 0.884], *β* = 1.00, *p* < 0.001, whereas the screen-time coefficient was close to zero, *B* = −0.004 [−0.010, 0.001], *β* = −0.03, *p* = 0.135. When the entry order was reversed, reward sensitivity added Δ*R*^2^ = 0.239 [0.207, 0.269] beyond the covariates, and screen time then added only Δ*R*^2^ = 0.0002 [0.000, 0.001], Δ*F*(1, 629) = 2.17, *p* = 0.141. A standardized coefficient of approximately 1.0 for a predictor of a bounded outcome is a further sign that reward sensitivity functions in this model as a near-duplicate of the outcome. The attenuation of the screen-time coefficient is therefore the expected arithmetic consequence of adding a variable that shares about 89% of the outcome’s variance; it is neither evidence of temporal mediation nor evidence that reward sensitivity is a distinct explanatory mechanism.

### Regression diagnostics

For the Step 3 model, residuals were approximately symmetric (skewness = −0.05, excess kurtosis = −0.15); the Shapiro–Wilk test was significant (*W* = 0.972, *p* < 0.001), as expected for minor departures with *N* = 640. Residual variance was heterogeneous, Breusch–Pagan *χ*^2^(10) = 146.57, *p* < 0.001, which motivated the HC3 standard errors; inferences did not differ from those based on conventional standard errors. The RESET test indicated some misspecification, *F*(2, 627) = 8.32, *p* < 0.001, but quadratic terms for screen time (*p* = 0.167) and reward sensitivity (*p* = 0.280) were nonsignificant. In the Step 2 model, the screen-time association was concave (quadratic *B* = −0.005, *p* < 0.001), that is, it flattened at higher reported hours. The Durbin–Watson statistic was 1.97. No case had Cook’s *D* > 1 (maximum = 0.045); 12 cases exceeded 4/*N*, the maximum leverage was 0.070, and 4 cases had absolute studentized residuals > 3. Excluding the 12 most influential cases did not change the focal estimates (Table 2).

### Collinearity sensitivity analysis

Age was fully nested within educational level, which produced VIF = 15.51 for age and 5.94–6.77 for the education indicators in the full model. A sensitivity model omitted age while retaining the educational-level indicators; all VIFs then fell at or below 4.53. The focal results were unchanged: *R*^2^ = 0.933; total screen time remained near zero, *B* = −0.004 [−0.010, 0.002], *p* = 0.153, and reward sensitivity remained strongly associated with mood dysregulation, *B* = 0.853 [0.819, 0.888], *p* < 0.001. The attenuation pattern therefore did not depend on simultaneously including age and educational level.

### Additional sensitivity analyses

Table 2 summarizes further robustness checks. The positive adjusted association of screen time with mood dysregulation before reward sensitivity was entered was robust to excluding influential cases and to excluding the 122 cases flagged by the careless-responding screens (*B* = 0.034, *p* < 0.001), and it was present in both age bands (13–16 years: *B* = 0.042; 17–19 years: *B* = 0.029; both *p* ≤ 0.001), although the adjusted slope was smaller in the older band (screen time × late-adolescence interaction *B* = −0.031, *p* = 0.002). It was weaker and not statistically significant, however, in the small subsample (*n* = 213) that showed no long-string responding on either scale (*B* = 0.015, *p* = 0.078). After reward sensitivity was added, the screen-time coefficient was near zero in most specifications but became small and negative with a minimal covariate set (*B* = −0.008, *p* < 0.001) and in late adolescence (*B* = −0.011, *p* = 0.019), illustrating that the residual screen-time coefficient after adjustment for a near-duplicate of the outcome is unstable and should not be interpreted. Finally, when the four screen-time domains replaced the total, they jointly explained additional variance beyond the covariates [Δ*R*^2^ = 0.120, *F*(4, 627) = 102.48, *p* < 0.001], but social media, streaming/video, and browsing/communication were so highly intercorrelated (*r* = 0.87–0.97) that their individual coefficients were unstable and showed sign reversals (social media *B* = 0.889; streaming/video *B* = −0.465; browsing/communication *B* = −0.437; gaming *B* = −0.015, *p* = 0.475). Domain-specific inferences are therefore limited to the bivariate correlations reported above.

### Summary of findings

Five findings are central. First, the reward-sensitivity and mood-dysregulation scales were not empirically distinguishable (latent *r* ≈ 1.0; HTMT = 0.98), a result that may reflect construct overlap, shared response styles, or common-method variance. Second, reported screen time showed large concurrent associations with both scales (*r* = 0.53 and 0.63), which were stronger in late than in early/middle adolescence. Third, screen time had a significant adjusted association with mood dysregulation before reward sensitivity was entered, but its coefficient became near zero afterwards, and it added virtually no variance beyond reward sensitivity when the entry order was reversed; given the first finding, this pattern does not indicate a distinct reward mechanism. Fourth, demographic covariates, especially parental education and school type, which were strongly confounded, explained an unusually large share of the outcome variance. Fifth, the focal pattern was robust to collinearity, influential cases, and careless-responding exclusions, although the pre-adjustment screen-time association was weaker in the subsample without long-string responding.

## Discussion

The present study examined how self-reported screen time, reward sensitivity, and mood dysregulation covary in Egyptian adolescents and whether the two psychological scales are empirically distinct. Screen time showed large concurrent associations with both scales by conventional benchmarks. The central finding, however, is psychometric: the reward-sensitivity and mood-dysregulation scales were empirically indistinguishable, and a one-factor model represented their 13 items as well as the hypothesized two-factor model. The hierarchical regression showed that the screen-time coefficient fell to near zero once reward sensitivity was entered, but given this overlap the pattern indicates little more than that screen time explains no variance in mood dysregulation beyond a near-duplicate measure of the same self-reported dimension. Cross-sectional data, moreover, cannot determine the temporal order of any of these associations.

The screen-time–reward-sensitivity correlation (*r* = 0.63) is broadly consistent with developmental and neurocognitive work linking screen use, reward orientation, salience processing, and cognitive control (Chen et al., 2023; Maza et al., 2023). However, the present measure captures estimated hours of use rather than the rewarding features encountered during that use. It therefore cannot establish that platform reinforcement caused higher reward sensitivity. A more defensible interpretation is that adolescents who report more screen time also tend, in this dataset, to report greater reward responsiveness, and equally greater irritability and emotional reactivity, because the two self-report scales captured a common dimension. The domain-level correlations, which were strongest for social media and weakest for gaming, further show that the total-hours index aggregates heterogeneous activities. We retained the total because it was the pre-specified exposure, but this result is consistent with the argument that screen time should not be treated as a unitary exposure, and future work should treat activity-specific indicators as primary.

The near-unity association between the two scales deserves particular emphasis because it changes what the study can claim. Reward responsivity and affect-regulation difficulty can be theoretically connected during adolescence (Duttweiler et al., 2024; Qiu et al., 2025; Paulus et al., 2021), but theoretical connection implies correlated, not identical, constructs. Several explanations are possible: genuine construct overlap in this population; overlapping content, since both scales emphasize emotional intensity; shared response styles such as acquiescence or a general tendency to endorse intense affect, which the high rate of long-string responding makes plausible; common-method variance arising from same-source, same-session self-report with similar Likert formats and uniformly positive keying (Podsakoff et al., 2012); or problems in data collection or processing. The item-level evidence, with semantically distinct items correlating as high as 0.99, favors response-process or method explanations over substantive overlap, but the present dataset cannot adjudicate among them. What is clear is that, in these data, reward sensitivity and mood dysregulation cannot be treated as separate constructs, and the regression coefficient for reward sensitivity cannot be read as evidence that a distinct reward mechanism explains mood dysregulation. Future work should use validated full-length instruments with established discriminant validity, mixed item keying to control acquiescence, temporally separated or multi-informant assessment, and behavioral reward tasks.

The three-step regression must be read in that light. Screen time contributed a modest but significant 1.7% increment in explained variance after demographic and contextual adjustment. Reward sensitivity then contributed a much larger increment, and with the order reversed screen time contributed essentially nothing beyond reward sensitivity. Because the two psychological scales correlate at 0.94, this large increment is mathematically unsurprising and does not indicate greater explanatory relevance; we therefore frame the hierarchy strictly as a conditional-association analysis rather than an explanatory decomposition. The attenuation is not mediation, which would require temporal ordering and construct distinctiveness that the current design cannot provide, and the most parsimonious reading is that screen time is associated with a single reward–affective reactivity self-report dimension. Longitudinal or intensive repeated-measures data with psychometrically distinct measures would be needed to determine whether reward sensitivity precedes, follows, or reciprocally co-develops with mood dysregulation and screen use.

Group differences add a contextual dimension. Males reported somewhat more screen time (a small effect), whereas females reported higher mood dysregulation (a medium effect) and modestly higher reward sensitivity (a small effect). These patterns should not be taken to imply inherent gender differences in vulnerability; platform preferences, social-evaluative experiences, activity types, and reporting tendencies were not measured, and because gender was recorded as a binary self-report, the comparisons cannot distinguish sex from gender identity. The literature on social comparison and maladaptive emotion regulation provides plausible contextual hypotheses, but the present data cannot distinguish among them (Castillo-Gualda et al., 2026).

School/institution type produced very large contrasts (|*d*| = 1.36–2.22). Effects of this size are unusual and call for caution. In this sample, school type was strongly confounded with parental education (Cramér’s *V* = 0.63) and residence, and within the extreme parental-education strata, public- and private-school participants did not differ in mood dysregulation. The large school-type and parental-education coefficients, and the unusually high variance explained by demographic covariates alone (*R*^2^ = 0.695), may therefore reflect genuine socioeconomic inequalities, the non-probability selection of institutions, clustering of participants within a small number of institutions, or recruitment-site differences in response style. Because institutional identifiers were not retained, these possibilities cannot be separated, and standard errors that ignore clustering may be too small. The sign reversal of the public-school coefficient after reward sensitivity enters the regression further underscores that adjusted demographic coefficients in a highly intercorrelated model should not be overinterpreted.

Educational level was associated with screen time, with high-school students reporting about three more hours than both other groups, but not with reward sensitivity or mood dysregulation. Because age was fully nested within educational level, the regression diagnostics appropriately revealed collinearity when both were entered together, and removing age resolved the diagnostic problem without changing the focal conclusions. Age-stratified analyses further showed that the bivariate associations of screen time with both psychological scales were stronger in late than in early/middle adolescence, although the covariate-adjusted slope was somewhat smaller in the older group. These developmental differences merit testing in longitudinal designs.

The findings are compatible with interactional accounts of problematic media use, which conceptualize digital behavior as a product of dispositional vulnerabilities, environmental affordances, and maintaining feedback processes rather than exposure alone (Domoff et al., 2020). They are also consistent with longitudinal evidence that emotional difficulties and problematic technology use can be reciprocal (Su et al., 2026). The present data, however, only locate the three variables, two of which could not be distinguished, within the same concurrent statistical system. Accordingly, the hypothesized processes shown with dashed arrows in Figure 1 remain untested by this study.

Applied implications should be similarly proportionate to the evidence. The results do not justify a clinical screening rule based on reward sensitivity, nor do they establish that reducing screen hours would prevent mood dysregulation. Summed self-reported hours of the magnitude observed here should not, by themselves, be treated as problematic, given that duration is a crude metric of use (Orben and Przybylski, 2019; Parry et al., 2021). The findings do suggest that research and prevention programs may benefit from considering why and how adolescents engage with rewarding digital cues alongside duration. Interventions that strengthen emotion regulation, reflective media habits, and cognitive control are theoretically relevant, but their effectiveness for the pattern observed here requires direct experimental or longitudinal evaluation (Tu et al., 2025).

### Limitations and future directions

Several limitations constrain inference. First, the cross-sectional design precludes causal and temporal conclusions. Second, and most important, the reward-sensitivity and mood-dysregulation scales were not empirically distinguishable. Because both were author-adapted short forms administered by the same method in one session, the overlap may reflect common-method variance, response styles (a large proportion of participants gave identical responses to all items of a scale), or construct overlap, and the data cannot distinguish among these. This limitation affects the interpretation of every model that includes both variables. Third, screen time was based on estimated hours on a typical day; it did not distinguish weekdays from weekends, active from passive use, or educational from recreational use, and it did not record simultaneous activities. Summed estimates can therefore exceed clock time, and the near-collinearity of the domain estimates suggests that respondents may have scaled their answers across categories. Fourth, sampling was non-probabilistic, and the findings should not be generalized to Egyptian adolescents as a whole. Fifth, participants were nested within institutions, but institutional identifiers were not available, so the independence assumption of ordinary least squares may be violated and standard errors may be underestimated. Sixth, demographic covariates, especially parental education and school type, which were strongly confounded, explained an unusually large share of the variance, and their coefficients should not be interpreted substantively. Seventh, age and educational level were fully nested, although a sensitivity model reproduced the focal conclusion. Finally, the instruments lacked prior validation evidence in Egyptian adolescents, and gender was recorded as a binary category.

Future studies should use longitudinal or ecological-momentary designs; device-logged measures of digital use; platform- and activity-specific indicators with explicit handling of simultaneous use; validated full-length reward-sensitivity and irritability or emotion-dysregulation instruments with established Arabic-language validity and mixed item keying; behavioral reward-processing tasks; multiple informants; probability or well-documented sampling with institutional identifiers that permit multilevel modelling; and preregistered analysis plans. Such designs would allow the hypothesized processes in Figure 1 to be evaluated directly and would clarify whether the overlap observed here reflects shared trait vulnerability, common-method variance, response styles, or construct redundancy.

## Conclusion

In this convenience sample of 640 Egyptian adolescents, self-reported total daily screen time showed large concurrent associations with reward sensitivity and mood dysregulation. The two psychological scales, however, were not empirically distinguishable: their latent correlation approached 1.0, and a single factor represented their items as well as two factors did. Screen time had a significant covariate-adjusted association with mood dysregulation that disappeared once reward sensitivity was added, a pattern broadly reproduced across sensitivity analyses, although the pre–reward-sensitivity screen-time coefficient was not statistically significant in the strict subsample without long-string responding (*n* = 213; *p* = 0.078).

The most defensible conclusion is therefore not that reward sensitivity mediates, causes, or explains the emotional correlates of screen time, but that screen time is associated with a single self-reported dimension of reward-related and affective reactivity that the two scales jointly measured. The near-unity overlap between the two psychological scales is itself an important finding and a measurement caution for the field. The study supports moving beyond screen duration as the sole explanatory variable while also showing why psychometrically discriminating, multimethod, and longitudinal designs are needed before developmental mechanisms or intervention targets can be established.

For practice, the findings encourage attention to the motivational and emotional context of digital use rather than blanket assumptions about hours alone. For research, they provide a clearer starting point: reported screen time and self-reported reward–affective reactivity are tightly connected in this dataset, but the direction, causal structure, and dimensionality of those connections remain open questions.

## Data Availability

The original contributions presented in the study are included in the article/Supplementary material, further inquiries can be directed to the corresponding author.
