# Supplementary material for: Screen time, reward sensitivity, and mood dysregulation among Egyptian adolescents: concurrent associations and a test of construct distinctiveness
Source: Front Psychol. 2026 Oct 28;17:2006153. doi: 10.3389/fpsyg.2026.2006153 (PMC13752049; doi:10.3389/fpsyg.2026.2006153)
Supplement: Supplementary file 1 [file Data_Sheet_1.pdf]

Supplementary Research Package

Appendices, Participant-Level Raw Data, Statistical Tables, and Reproducible R Code

Manuscript 2006153 — Frontiers in Psychology

8 October 2026

Package provenance and usage

This integrated supplement preserves the supplied 57-page *Data Sheet 1.PDF* as a source appendix (including instruments, coding key, all 640 participant-level records displayed in ID-linked panels, and historical statistical tables), followed by tables extracted from the corrected manuscript and the full corrected R analysis script. The machine-readable `data.csv` is delivered alongside this PDF and was transcribed programmatically from those three PDF panels, preserving their numeric values. It has 640 unique participant IDs and 24 columns. The CSV has not been independently authenticated against the original study data export; its origin is the provided PDF.

Important qualification: The source Data Sheet 1 was prepared before the final round of manuscript corrections. Where wording or analysis interpretation differs, the corrected manuscript and corresponding R analysis take precedence. The source PDF includes legacy wording (including the university-year label), and is included as a transparent archival source rather than silently rewritten evidence. Data are observational and analysis does not establish mediation or causation. Institutional ethics approval was retrospective, issued 25 February 2026 after the 2023–2024 data collection during an ethics-committee transition; no statement of prior ethics approval should be inferred from this package.

Reproducibility directory

- `data.csv`: participant-level dataset transcribed from the supplied appendix tables; columns `ID, Age, Gender, Edu, School, Parent, Res, Social, Gaming, Stream, Browse, RS1-RS6, MD1-MD7`.
- `Supplementary_Code_1_analysis.R`: corrected analysis script, which requires `data.csv` in the working directory and the indicated R packages.
- `Data Sheet 1.PDF`: unchanged source document supplied in the original supplementary ZIP.

Contents

1 Updated statistical tables from corrected manuscript **2**

2 Full Supplementary Code 1 (R) **10**

3 Source appendices, participant-level data panels and historic statistical tables **14**

1 Updated statistical tables from corrected manuscript

Manuscript table 1

Characteristic	n	%
Gender (binary self-report)		
Male	322	50.3
Female	318	49.7
Educational level (age range)		
Middle school (13–14 years)	160	25.0
High school (15–17 years)	288	45.0
University, early undergraduate years (18–19 years)	192	30.0
Age band		
Early/middle adolescence (13–16 years)	352	55.0
Late adolescence (17–19 years)	288	45.0
School/institution type		
Public	429	67.0
Private / International	211	33.0
Parental education		
Basic / Secondary	241	37.7
University degree	293	45.8
Postgraduate degree	106	16.6
Residence		
Urban	423	66.1
Semi-urban / Rural	217	33.9

Manuscript table 2

Estimator / model	χ^2 (df)	CFI	TLI	RMSEA [90% CI]	SRMR	Latent r [95% CI]
WLSMV (ordinal items)						
Two-factor	577.58 (64)	1.000	1.000	.112 [.104, .121]	.061	.997 [.996, .999]
One-factor	587.12 (65)	1.000	1.000	.112 [.104, .121]	.061	—
MLR (continuous items)						
Two-factor	2030.12 (64)	.748	.693	.316 [.304, .328]	.058	.998 [.994, 1.002]
One-factor	1977.08 (65)	.748	.698	.314 [.302, .325]	.058	—

Manuscript table 3

Variable	M	SD	Min	Max	Skewness	Kurtosis	α	ω
Age	16.13	1.97	13.00	19.00	–0.08	–1.19	—	—
Social media screen time	3.87	1.28	1.10	7.40	0.34	–0.58	—	—
Gaming screen time	2.58	1.46	0.10	6.60	0.80	–0.03	—	—
Streaming/video screen time	2.18	0.86	0.50	4.80	0.49	–0.29	—	—
Browsing/communication screen time	1.33	0.68	0.30	3.60	0.80	0.08	—	—
Total daily screen time	9.96	3.76	2.50	20.10	0.42	–0.48	—	—
Reward sensitivity	3.05	0.74	1.67	4.00	–0.05	–1.52	.96	.96
Mood dysregulation	2.07	0.62	1.00	3.00	–0.03	–1.13	.96	.96

Manuscript table 4

Variable	1	2	3	4
1. Age	—			
2. Total screen time	.13** [.05, .20]	—		
3. Reward sensitivity	.15*** [.08, .23]	.63*** [.58, .67]	—	
4. Mood dysregulation	.05 [−.03, .12]	.53*** [.47, .58]	.94*** [.93, .95]	—

Manuscript table 5

Comparison	Outcome	Group means	Test	p	Effect size [95% CI]
Gender	Total screen time	Male = 10.42; Female = 9.50	$t(638.0) = 3.14$	.002	$d = 0.25$ [0.09, 0.40]
Gender	Reward sensitivity	Male = 2.93; Female = 3.16	$t(636.4) = -4.05$	$< .001$	$d = -0.32$ [−0.48, −0.16]
Gender	Mood dysregulation	Male = 1.89; Female = 2.25	$t(627.3) = -7.62$	$< .001$	$d = -0.60$ [−0.76, −0.44]
School type	Total screen time	Public = 8.54; Private = 12.86	$t(371.9) = -15.47$	$< .001$	$d = -1.36$ [−1.54, −1.18]
School type	Reward sensitivity	Public = 2.67; Private = 3.81	$t(632.9) = -31.97$	$< .001$	$d = -2.22$ [−2.43, −2.02]
School type	Mood dysregulation	Public = 1.79; Private = 2.65	$t(518.3) = -23.56$	$< .001$	$d = -1.83$ [−2.02, −1.63]
Education level	Total screen time	Middle = 8.41; High = 11.71; University = 8.64	$F(2, 637) = 68.22$	$< .001$	$\eta^2 = .18$ [.13, .23]
Post hoc	High – Middle	$\Delta = 3.30$ [2.49, 4.11]	Games–Howell	$< .001$	$d = 0.92$
Post hoc	High – University	$\Delta = 3.07$ [2.34, 3.79]	Games–Howell	$< .001$	$d = 0.89$
Post hoc	Middle – University	$\Delta = -0.23$ [−1.04, 0.57]	Games–Howell	.776	$d = -0.07$
Education level	Reward sensitivity	Middle = 3.04; High = 3.06; University = 3.03	$F(2, 637) = 0.14$	.873	$\eta^2 < .001$ [.00, .01]
Education level	Mood dysregulation	Middle = 2.17; High = 2.05; University = 2.03	$F(2, 637) = 2.63$	.073	$\eta^2 = .01$ [.00, .03]

Manuscript table 6

Step	Predictor	B	SE (HC3)	95% CI (HC3)	β	p
1	Intercept	3.095	.450	[2.210, 3.979]		< .001
1	Male	−.160	.038	[−.235, −.085]		< .001
1	Middle school	.028	.081	[−.130, .187]		.726
1	University	.180	.075	[.033, .327]		.017
1	Public school	−.387	.043	[−.471, −.303]		< .001
1	Parent education: postgraduate	.950	.057	[.838, 1.063]		< .001
1	Parent education: university	.505	.043	[.420, .590]		< .001
1	Urban residence	.206	.045	[.117, .295]		< .001
1	Age	−.079	.029	[−.136, −.022]	−.25	.007
2	Intercept	2.680	.457	[1.782, 3.578]		< .001
2	Male	−.226	.040	[−.304, −.148]		< .001
2	Middle school	.142	.085	[−.024, .308]		.093
2	University	.276	.074	[.131, .421]		< .001
2	Public school	−.320	.041	[−.401, −.240]		< .001
2	Parent education: postgraduate	.779	.064	[.652, .905]		< .001
2	Parent education: university	.429	.044	[.342, .516]		< .001
2	Urban residence	.175	.044	[.089, .261]		< .001
2	Age	−.074	.029	[−.130, −.017]	−.23	.010
2	Total screen time	.035	.006	[.024, .047]	.21	< .001
3	Intercept	.277	.227	[−.168, .722]		.222
3	Male	−.117	.019	[−.153, −.080]		< .001
3	Middle school	−.007	.040	[−.087, .072]		.855
3	University	.119	.032	[.056, .182]		< .001
3	Public school	.066	.020	[.026, .106]		.001
3	Parent education: postgraduate	.046	.031	[−.014, .107]		.132
3	Parent education: university	−.110	.021	[−.151, −.068]		< .001
3	Urban residence	.033	.021	[−.008, .074]		.118
3	Age	−.047	.014	[−.074, −.020]	−.15	< .001
3	Total screen time	−.004	.003	[−.010, .001]	−.03	.135
3	Reward sensitivity	.850	.017	[.816, .884]	1.00	< .001

Manuscript table 7

Specification	n	Screen time, Step 2: B [95% CI], p	Screen time, Step 3: B [95% CI], p	Reward sensitivity, Step 3: B [95% CI]	R^2 (Step 3)
Primary model	640	0.035 [0.024, 0.047], < .001	–0.004 [–0.010, 0.001], .135	0.850 [0.816, 0.884]	.935
Age omitted	640	0.036 [0.024, 0.047], < .001	–0.004 [–0.010, 0.002], .153	0.853 [0.819, 0.888]	.933
Cook's D > 4/N excluded	628	0.033 [0.022, 0.045], < .001	–0.004 [–0.009, 0.001], .158	0.852 [0.821, 0.882]	.943
Careless-responding screens excluded	518	0.034 [0.021, 0.047], < .001	–0.002 [–0.009, 0.004], .436	0.828 [0.793, 0.864]	.917
No long-string responding on either scale	213	0.015 [–0.002, 0.032], .078	–0.004 [–0.014, 0.006], .479	0.755 [0.612, 0.899]	.925
Minimal covariates (gender, age)	640	0.095 [0.086, 0.104], < .001	–0.008 [–0.013, –0.003], < .001	0.821 [0.799, 0.843]	.921
Ages 13–16	352	0.042 [0.026, 0.057], < .001	0.001 [–0.007, 0.009], .766	0.845 [0.804, 0.886]	.938
Ages 17–19	288	0.029 [0.012, 0.047], .001	–0.011 [–0.020, –0.002], .019	0.849 [0.791, 0.907]	.939

2 Full Supplementary Code 1 (R)

```r
# REPRODUCIBILITY PRE-FLIGHT: This script requires an authentic 640-row data.csv.
# The file is not included in the currently supplied attachments.
# Do not synthesize or reconstruct records to make the script appear executable.
if (!file.exists("data.csv")) stop("Missing authentic data.csv (640 participant rows). Deposit the verified CSV beside this script before running.")
.preflight <- read.csv("data.csv", check.names=FALSE)
.required <- c("ID","Age","Gender","Edu","School","Parent","Res","Social","Gaming","Stream","Browse",paste0("RS",1:6),paste0("MD",1:7))
if (!all(.required %in% names(.preflight))) stop("Input is missing expected columns: ",paste(setdiff(.required,names(.preflight)), collapse=", "))
if (nrow(.preflight)!=640L) stop("Expected 640 participant records; got ", nrow(.preflight))
rm(.preflight,.required)

# ====================================================================
# Supplementary Code 1
# Screen Time, Reward Sensitivity, and Mood Dysregulation Among Egyptian
# Adolescents: Concurrent Associations and a Test of Construct Distinctiveness
#
# Intended analysis reproduction; end-to-end execution requires the authentic data.csv and software dependencies.
# Input: data.csv, the 640-row participant-level dataset in Supplementary
# Data Sheet 1 (columns: ID, Age, Gender, Edu, School, Parent, Res, Social,
# Gaming, Stream, Browse, RS1-RS6, MD1-MD7; coding as in Section B.2).
# Tested with R 4.3.3, lavaan 0.6-17, semTools 0.5-6, psych 2.4.1,
# sandwich 3.1-0, lmtest 0.9-40, car 3.1-2.
# ====================================================================

## ---- Part 1. Measurement models, HTMT, omega, EFA, Harman ----
suppressMessages({library(lavaan);library(semTools);library(psych)})
d<-read.csv("data.csv")
rs<-paste0("RS",1:6); md<-paste0("MD",1:7)
for(v in c(rs,md)) d[[v]]<-ordered(d[[v]])
m2<-paste("RS =~",paste(rs,collapse="+"),"\nMD =~",paste(md,collapse="+"))
m1<-paste("G =~",paste(c(rs,md),collapse="+"))
f2<-cfa(m2,d,ordered=c(rs,md),estimator="WLSMV")
f1<-cfa(m1,d,ordered=c(rs,md),estimator="WLSMV")
fm<-c("chisq.scaled","df.scaled","pvalue.scaled","cfi.scaled","tli.scaled","rmsea.scaled","rmsea.ci.lower.scaled","rmsea.ci.upper.scaled","srmr")
print(round(fitMeasures(f2,fm),3)); print(round(fitMeasures(f1,fm),3))
print(lavTestLRT(f1,f2,method="satorra.2000"))
pe<-parameterEstimates(f2,standardized=TRUE)
print(pe[pe$op=="=~",c("lhs","rhs","std.all")])
print(pe[pe$op=="~~"&pe$lhs=="RS"&pe$rhs=="MD",c("est","ci.lower","ci.upper","std.all")])
# latent corr fixed to 1 test
m2f<-paste(m2,"\nRS~~1*MD\nRS~~1*RS\nMD~~1*MD")
f2f<-cfa(m2f,d,ordered=c(rs,md),estimator="WLSMV",std.lv=TRUE)
f2s<-cfa(m2,d,ordered=c(rs,md),estimator="WLSMV",std.lv=TRUE)
print(lavTestLRT(f2f,f2s,method="satorra.2000"))
print(parameterEstimates(f2s)[parameterEstimates(f2s)$lhs=="RS"&parameterEstimates(f2s)$rhs=="MD",c("est","se","ci.lower","ci.upper")])
cat("HTMT\n"); dn<-read.csv("data.csv"); print(htmt(m2,dn))
cat("omega\n"); print(compRelSEM(f2s));
dn2<-dn; print(compRelSEM(cfa(m2,dn,std.lv=TRUE)))
# AVE
print(AVE(f2s))
# ESEM-like EFA with polychoric
pc<-polychoric(dn[,c(rs,md)])$rho
e<-fa(pc,nfactors=2,n.obs=640,rotate="geominQ",fm="wls"); print(e$loadings,cutoff=0); print(e$Phi)
e1<-fa(pc,nfactors=2,n.obs=640,rotate="oblimin",fm="minres"); print(e1$Phi)
print(eigen(pc)$values)
cat("Harman PCA first factor pct (Pearson):\n"); ev<-eigen(cor(dn[,c(rs,md)]))$values; print(ev[1]/sum(ev))
# cross-scale item correlations
ic<-cor(dn[,c(rs,md)]); print(round(ic[rs,md],2)); print(range(ic[rs,md]));
w1<-ic[rs,rs][upper.tri(ic[rs,rs])]; w2<-ic[md,md][upper.tri(ic[md,md])]; print(c(mean(w1),mean(w2),mean(ic[rs,md])))
print(alpha(dn[,rs])$total$raw_alpha); print(alpha(dn[,md])$total$raw_alpha)
save(f1,f2,f2s,file="cfa.RData")

## ---- Part 2. Continuous (MLR) CFA robustness check ----
suppressMessages({library(lavaan);library(sandwich);library(lmtest);library(psych)})
d<-read.csv("data.csv")
```

```
rs<-paste0("RS",1:6); md<-paste0("MD",1:7)
m2<-paste("RS =~",paste(rs,collapse="+"),"\nMD =~",paste(md,collapse="+"))
m1<-paste("G =~",paste(c(rs,md),collapse="+"))
f2<-cfa(m2,d,estimator="MLR",std.lv=TRUE); f1<-cfa(m1,d,estimator="MLR",std.lv=TRUE)
fm<-c("chisq.scaled","df.scaled","cfi.robust","tli.robust","rmsea.robust","rmsea.ci.lower.robust","rmsea.ci.upper.robust","srmr","aic","bic")
print(round(fitMeasures(f2,fm),3)); print(round(fitMeasures(f1,fm),3)); print(lavTestLRT(f1,f2))
pe<-parameterEstimates(f2,standardized=TRUE); print(pe[pe$lhs=="RS"&pe$rhs=="MD",c("est","se","ci.lower","ci.upper")])
print(range(pe$std.all[pe$op=="=~"]))

## ---- Part 3. Hierarchical regression, HC3, CIs, diagnostics, reversed order, bootstrap ----
suppressMessages({library(sandwich);library(lmtest);library(psych);library(car)})
d<-read.csv("data.csv")
rs<-paste0("RS",1:6); md<-paste0("MD",1:7)
set.seed(2026)
pa<-fa.parallel(d[,c(rs,md)],fa="pc",plot=FALSE,n.iter=50); cat("PA ncomp",pa$ncomp,"\n")
d$ST<-d$Social+d$Gaming+d$Stream+d$Browse; d$RSm<-rowMeans(d[,rs]); d$MDm<-rowMeans(d[,md])
d$male<-as.integer(d$Gender==1); d$mid<-as.integer(d$Edu==1); d$uni<-as.integer(d$Edu==3)
d$pub<-as.integer(d$School==1); d$ppg<-as.integer(d$Parent==3); d$puni<-as.integer(d$Parent==2); d$urb<-as.integer(d$Res==1)
cov<-"male+mid+uni+pub+ppg+puni+urb+Age"
m1<-lm(as.formula(paste("MDm~",cov)),d); m2<-update(m1,.~.+ST); m3<-update(m2,.~.+RSm); m2r<-update(m1,.~.+RSm)
for(m in list(m1,m2,m3,m2r)) cat(summary(m)$r.squared, "\n")
print(anova(m1,m2,m3)); print(anova(m2r,m3)); cat("unique ST after RS dR2", summary(m3)$r.squared-summary(m2r)$r.squared,"\n")
cat("RS after cov dR2",summary(m2r)$r.squared-summary(m1)$r.squared,"\n")
# CIs, std betas, HC3
z<-d; for(v in c("MDm","ST","RSm","Age")) z[[v]]<-scale(z[[v]])
for(m in list(m1,m2,m3)){
 print(round(cbind(coef(m),confint(m)),3)); ct<-coeftest(m,vcov=vcovHC(m,"HC3")); print(round(ct,3)); print(round(coefci(m,vcov.=vcovHC(m,"HC3")),3))
 ms<-lm(formula(m),z); print(round(coef(ms),3)) }
cat("VIF m3\n"); print(round(vif(m3),2))
# diagnostics m3 and m2
diag<-function(m,nm){ r<-rstudent(m); cat(nm,"\n"); cat("resid skew/kurt", skew(resid(m)), kurtosi(resid(m)), "\n"); print(shapiro.test(resid(m)))
 print(bptest(m)); print(resettest(m,power=2:3)); cd<-cooks.distance(m); h<-hatvalues(m); k<-length(coef(m))
 cat("maxCook",max(cd),"n>4/n",sum(cd>4/nrow(d)),"n>1",sum(cd>1),"maxlev",max(h),"n lev>2k/n",sum(h>2*k/nrow(d)),"n |rstud|>3",sum(abs(r)>3),"\n")
 cat("durbin",dwtest(m)$statistic,"\n")}
diag(m2,"M2"); diag(m3,"M3")
# refit excluding cooks>4/n
cd<-cooks.distance(m3); m3x<-update(m3,data=d[cd<=4/nrow(d),]); m2x<-update(m2,data=d[cd<=4/nrow(d),]); cat("excl n",nobs(m3x),"\n"); print(round(coef(summary(m3x))[c("ST","RSm"),],4)); print(round(coef(summary(m2x))["ST",],4))
# bootstrap dR2
B<-2000; bs<-replicate(B,{i<-sample(nrow(d),replace=TRUE); dd<-d[i,]; a<-summary(lm(formula(m1),dd))$r.squared; b<-summary(lm(formula(m2),dd))$r.squared; c<-summary(lm(formula(m3),dd))$r.squared; e<-summary(lm(formula(m2r),dd))$r.squared; c(a,b-a,c-b,c-e,e-a)})
print(round(apply(bs,1,quantile,c(.025,.975)),3))
save(d,file="d.RData")

## ---- Part 4. Data integrity, age strata, domain models, confounding ----
suppressMessages({library(sandwich);library(lmtest);library(psych)})
load("d.RData"); rs<-paste0("RS",1:6); md<-paste0("MD",1:7)
cov<-"male+mid+uni+pub+ppg+puni+urb+Age"
cat("== crosstab age x edu\n"); print(table(d$Age,d$Edu))
cat("== school x parent\n"); print(table(d$School,d$Parent)); print(round(prop.table(table(d$School,d$Parent),1),3))
cat("== school x edu, school x res, school x gender\n"); print(table(d$School,d$Edu)); print(table(d$School,d$Res)); print(table(d$School,d$Gender))
cat("chi school-parent"); print(chisq.test(table(d$School,d$Parent))); print(cramer<-sqrt(chisq.test(table(d$School,d$Parent))$statistic/640))
cat("== zeros per domain\n"); print(sapply(d[,c("Social","Gaming","Stream","Browse")],function(x) c(zero=sum(x==0),lt.5=sum(x<.5),min=min(x),max=max(x))))
cat("ST>16:",sum(d$ST>16),"ST>18",sum(d$ST>18),"\n")
cat("== integrity: dup rows", sum(duplicated(d[,c("Age","Gender","Edu","School","Parent","Res","Social","Gaming","Stream","Browse",rs,md)])),"\n")
sl_rs<-apply(d[,rs],1,function(x) length(unique(x))==1); sl_md<-apply(d[,md],1,function(x) length(unique(x))==1); cat("straightline RS",sum(sl_rs),"MD",sum(sl_md),"both",sum(sl_rs&sl_md),"\n")
cat("out of range:", sum(d[,rs]<1|d[,rs]>4), sum(d[,md]<1|d[,md]>3),"\n")
lz<-sapply(1:640,function(i){x<-c(scale(unlist(d[i,c(rs)])))});
```

```
mahal<-mahalanobis(d[,c(rs,md)],colMeans(d[,c(rs,md)]),cov(d[,c(rs,md)])); cat("mahal >crit .001", sum(mahal>qchisq(.999,13)),"\n")
cat("== RS item response distribution\n"); print(sapply(d[,rs],table)); print(sapply(d[,md],function(x) table(factor(x,1:3))))
cat("== within school type correlations\n")
for(s in 1:2){ss<-d[d$School==s,]; print(round(cor(ss[,c("ST","RSm","MDm")]),3))}
for(e in 1:3){ss<-d[d$Edu==e,]; cat("edu",e,"\n"); print(round(cor(ss[,c("ST","RSm","MDm")]),3))}
cat("== age strata\n"); d$late<-as.integer(d$Age>=17); print(table(d$late))
for(g in 0:1){ss<-d[d$late==g,]; cat("late",g,"n",nrow(ss),"\n"); print(round(cor(ss[,c("ST","RSm","MDm")]),3)); print(round(sapply(ss[,c("Age","ST","RSm","MDm")],function(x)c(M=mean(x),SD=sd(x))),2))
 cv<-"male+pub+ppg+puni+urb+Age"; a<-lm(as.formula(paste("MDm~",cv,"+ST")),ss); b<-lm(as.formula(paste("MDm~",cv,"+ST+RSm")),ss); print(round(coeftest(a,vcovHC(a,"HC3"))["ST",],4)); print(round(coeftest(b,vcovHC(b,"HC3"))[c("ST","RSm"),],4))}
mi<-lm(as.formula(paste("MDm~male+mid+uni+pub+ppg+puni+urb+Age+ST*late")),d); print(round(coeftest(mi,vcovHC(mi,"HC3"))["ST:late",],4))
mi2<-lm(as.formula(paste("RSm~male+mid+uni+pub+ppg+puni+urb+Age+ST*late")),d); print(round(coeftest(mi2,vcovHC(mi2,"HC3"))["ST:late",],4))
# Fisher z compare r(ST,MD) across strata
r1<-cor(d$ST[d$late==0],d$MDm[d$late==0]);r2<-cor(d$ST[d$late==1],d$MDm[d$late==1]);n1<-sum(d$late==0);n2<-sum(d$late==1); zz<-(atanh(r1)-atanh(r2))/sqrt(1/(n1-3)+1/(n2-3)); cat("fisher z MD",zz,2*pnorm(-abs(zz)),"\n")
r1<-cor(d$ST[d$late==0],d$RSm[d$late==0]);r2<-cor(d$ST[d$late==1],d$RSm[d$late==1]); zz<-(atanh(r1)-atanh(r2))/sqrt(1/(n1-3)+1/(n2-3)); cat("fisher z RS",zz,2*pnorm(-abs(zz)),"\n")
cat("== domain-specific models\n")
for(y in c("MDm","RSm")){ m<-lm(as.formula(paste(y,"~",cov,"+Social+Gaming+Stream+Browse")),d); print(round(cbind(coeftest(m,vcovHC(m,"HC3"))[c("Social","Gaming","Stream","Browse"),],coefci(m,vcov.=vcovHC(m,"HC3"))[c("Social","Gaming","Stream","Browse"),]),4)); cat("R2",summary(m)$r.squared,"\n")
 m0<-lm(as.formula(paste(y,"~",cov)),d); print(anova(m0,m))}
print(round(cor(d[,c("Social","Gaming","Stream","Browse","RSm","MDm")]),3))
m<-lm(as.formula(paste("MDm~",cov,"+Social+Gaming+Stream+Browse+RSm")),d); print(round(coeftest(m,vcovHC(m,"HC3"))[c("Social","Gaming","Stream","Browse","RSm"),],4))
cat("== ST -> RS adjusted\n"); m<-lm(as.formula(paste("RSm~",cov,"+ST")),d); print(round(coeftest(m,vcovHC(m,"HC3"))["ST",],4)); print(coefci(m,vcov.=vcovHC(m,"HC3"))["ST",]); m0<-lm(as.formula(paste("RSm~",cov)),d); cat("R2",summary(m0)$r.squared,summary(m)$r.squared,"\n")
cat("== composite\n"); d$comp<-rowMeans(scale(d[,c(rs,md)])); m0<-lm(as.formula(paste("comp~",cov)),d);m<-lm(as.formula(paste("comp~",cov,"+ST")),d); print(round(coeftest(m,vcovHC(m,"HC3"))["ST",],4)); cat("R2",summary(m0)$r.squared,summary(m)$r.squared,"\n"); print(cor(d$ST,d$comp)); print(alpha(d[,c(rs,md)])$total$raw_alpha)
zs<-d; zs$ST<-scale(zs$ST); zs$comp<-scale(zs$comp); print(round(coef(lm(as.formula(paste("comp~",cov,"+ST")),zs))["ST"],3))
cat("== nonlinearity\n"); m<-lm(as.formula(paste("MDm~",cov,"+ST+I(ST^2)+RSm+I(RSm^2)")),d); print(round(coeftest(m,vcovHC(m,"HC3"))[c("ST","I(ST^2)","RSm","I(RSm^2)"),],4))
m<-lm(as.formula(paste("MDm~",cov,"+ST+I(ST^2)")),d); print(round(coeftest(m,vcovHC(m,"HC3"))[c("ST","I(ST^2)"),],4))
cat("== demographics only R2 for RS and ST\n"); for(y in c("RSm","ST")){m0<-lm(as.formula(paste(y,"~",cov)),d); cat(y,summary(m0)$r.squared,"\n")}
cat("== parent-ed within school type means MD\n"); print(round(tapply(d$MDm,list(d$School,d$Parent),mean),2)); print(round(tapply(d$RSm,list(d$School,d$Parent),mean),2)); print(round(tapply(d$ST,list(d$School,d$Parent),mean),2))
cat("== school effect within parent strata\n"); for(p in 1:3){ss<-d[d$Parent==p,]; if(length(unique(ss$School))>1){tt<-t.test(MDm~School,ss); cat(p, tt$estimate, tt$p.value,"\n")}}
save(d,file="d.RData")

## ---- Part 5. Effect-size CIs, post hoc tests, power, careless-response sensitivity ----
suppressMessages({library(sandwich);library(lmtest);library(psych)})
load("d.RData"); rs<-paste0("RS",1:6); md<-paste0("MD",1:7)
cov<-"male+mid+uni+pub+ppg+puni+urb+Age"
# CIs for r
for(p in list(c("ST","MDm"),c("ST","RSm"),c("RSm","MDm"),c("Age","ST"),c("Age","RSm"),c("Age","MDm"))){ct<-cor.test(d[[p[1]]],d[[p[2]]]); cat(p, round(ct$estimate,3), round(ct$conf.int,3), ct$p.value,"\n")}
# d with CI
dci<-function(y,g){ x1<-y[g==1];x2<-y[g==2]; n1<-length(x1);n2<-length(x2); sp<-sqrt(((n1-1)*var(x1)+(n2-1)*var(x2))/(n1+n2-2)); dd<-(mean(x1)-mean(x2))/sp; se<-sqrt((n1+n2)/(n1*n2)+dd^2/(2*(n1+n2))); tt<-t.test(x1,x2); c(m1=mean(x1),m2=mean(x2),t=tt$statistic,df=tt$parameter,p=tt$p.value,d=dd,lo=dd-1.96*se,hi=dd+1.96*se)}
for(y in c("ST","RSm","MDm")){cat("gender",y,round(dci(d[[y]],d$Gender),3),"\n"); cat("school",y,round(dci(d[[y]],d$School),3),"\n")}
# ANOVA eta2 CI
for(y in c("ST","RSm","MDm")){a<-aov(d[[y]]~factor(d$Edu)); s<-summary(a)[[1]]; F<-s$`F value`[1]; e2<-s$`Sum Sq`[1]/sum(s$`Sum Sq`);
 ci<-psych::cohen.d.ci;
 # eta2 CI via noncentral F
 f<-function(ncp,q) pf(F,2,637,ncp)-q; lo<-tryCatch(uniroot(f,c(0,2000),q=.975)$root,error=function(e)0); hi<-uniroot(f,c(0,3000),q=.025)$root
```

```
 cat("edu",y,round(tapply(d[[y]],d$Edu,mean),2),"F",round(F,2),"p",s$`Pr(>F)`[1],"eta2",round(e2,3),"CI",round(lo/(lo+640),3),round(hi/(hi+640),3),"\n"); print(oneway.test(d[[y]]~factor(d$Edu)))}
# Games-Howell
gh<-function(y,g){g<-factor(g); lv<-levels(g); for(i in 1:2) for(j in (i+1):3){a<-y[g==lv[i]];b<-y[g==lv[j]]; na<-length(a);nb<-length(b); va<-var(a)/na; vb<-var(b)/nb; t<-(mean(a)-mean(b))/sqrt(va+vb); df<-(va+vb)^2/(va^2/(na-1)+vb^2/(nb-1)); p<-ptukey(abs(t)*sqrt(2),3,df,lower.tail=FALSE); sp<-sqrt(((na-1)*var(a)+(nb-1)*var(b))/(na+nb-2)); q<-qtukey(.95,3,df)/sqrt(2); cat(lv[i],lv[j],"diff",round(mean(a)-mean(b),2),"CI",round(mean(a)-mean(b)-q*sqrt(va+vb),2),round(mean(a)-mean(b)+q*sqrt(va+vb),2),"p",signif(p,3),"d",round((mean(a)-mean(b))/sp,2),"\n")}}
gh(d$ST,d$Edu); print(tapply(d$ST,d$Edu,sd))
# Power sensitivity: f2 for 1 df test with u=1, v=629, power .80
f<-function(f2) pf(qf(.95,1,629),1,629,ncp=f2*640,lower.tail=FALSE)-.80; cat("min f2",uniroot(f,c(1e-5,1))$root,"\n")
f<-function(r) pf(qf(.95,1,638),1,638,ncp=(r^2/(1-r^2))*640,lower.tail=FALSE)-.80; cat("min r",uniroot(f,c(1e-3,.9))$root,"\n")
f<-function(dd) power.t.test(n=211,delta=dd,sd=1)$power-.80; # approx unequal
cat("min d (n 429/211)", uniroot(function(dd) pwr_d<-pt(qt(.975,638),638,ncp=dd/sqrt(1/429+1/211),lower.tail=FALSE)-.80,c(.01,2))$root,"\n")
cat("min d gender", uniroot(function(dd) pt(qt(.975,638),638,ncp=dd/sqrt(1/322+1/318),lower.tail=FALSE)-.80, c(.01,2))$root,"\n")
# Sensitivity excluding straightliners on both scales, duplicates, multivariate outliers
sl_rs<-apply(d[,rs],1,function(x) length(unique(x))==1); sl_md<-apply(d[,md],1,function(x) length(unique(x))==1)
dup<-duplicated(d[,c("Age","Gender","Edu","School","Parent","Res","Social","Gaming","Stream","Browse",rs,md)])|duplicated(d[,c("Age","Gender","Edu","School","Parent","Res","Social","Gaming","Stream","Browse",rs,md)],fromLast=TRUE); cat("dup involved",sum(dup),"\n")
mahal<-mahalanobis(d[,c(rs,md)],colMeans(d[,c(rs,md)]),cov(d[,c(rs,md)])); mo<-mahal>qchisq(.999,13)
excl<-(sl_rs&sl_md)|mo|duplicated(d[,c("Age","Gender","Edu","School","Parent","Res","Social","Gaming","Stream","Browse",rs,md)])
dd<-d[!excl,]; cat("n after excl",nrow(dd),"\n")
cat("r",round(cor(dd[,c("ST","RSm","MDm")]),3),"\n")
m1<-lm(as.formula(paste("MDm~",cov)),dd);m2<-update(m1,.~.+ST);m3<-update(m2,.~.+RSm); cat("R2",summary(m1)$r.squared,summary(m2)$r.squared,summary(m3)$r.squared,"\n"); print(round(coeftest(m2,vcovHC(m2,"HC3"))["ST",],4)); print(round(coeftest(m3,vcovHC(m3,"HC3"))[c("ST","RSm"),],4))
# remove straightliners on either
dd2<-d[!(sl_rs|sl_md),]; cat("n no SL either",nrow(dd2),"r RS-MD",cor(dd2$RSm,dd2$MDm),"r ST-MD",cor(dd2$ST,dd2$MDm),"\n")
m2<-lm(as.formula(paste("MDm~",cov,"+ST")),dd2);m3<-update(m2,.~.+RSm); print(round(coeftest(m2,vcovHC(m2,"HC3"))["ST",],4)); print(round(coeftest(m3,vcovHC(m3,"HC3"))[c("ST","RSm"),],4))
# omit age model with HC3 and CIs
m3a<-lm(MDm~male+mid+uni+pub+ppg+puni+urb+ST+RSm,d); print(round(cbind(coeftest(m3a,vcovHC(m3a,"HC3"))[c("ST","RSm"),],coefci(m3a,vcov.=vcovHC(m3a,"HC3"))[c("ST","RSm"),]),4)); cat(summary(m3a)$r.squared,"\n"); print(round(car::vif(m3a),2))
m2a<-lm(MDm~male+mid+uni+pub+ppg+puni+urb+ST,d); print(round(coeftest(m2a,vcovHC(m2a,"HC3"))["ST",],4))
# Without parental ed & school (minimal covariates)
mm<-lm(MDm~male+Age+ST,d); print(round(coeftest(mm,vcovHC(mm,"HC3"))["ST",],4)); cat(summary(mm)$r.squared,"\n"); mm3<-update(mm,.~.+RSm); print(round(coeftest(mm3,vcovHC(mm3,"HC3"))[c("ST","RSm"),],4))

## ---- Part 6. Consolidated sensitivity table (Table 7) ----
suppressMessages({library(sandwich);library(lmtest)})
load("d.RData"); rs<-paste0("RS",1:6); md<-paste0("MD",1:7)
full<-"male+mid+uni+pub+ppg+puni+urb+Age"
sl_rs<-apply(d[,rs],1,function(x) length(unique(x))==1); sl_md<-apply(d[,md],1,function(x) length(unique(x))==1)
mahal<-mahalanobis(d[,c(rs,md)],colMeans(d[,c(rs,md)]),cov(d[,c(rs,md)])); mo<-mahal>qchisq(.999,13)
dupv<-duplicated(d[,c("Age","Gender","Edu","School","Parent","Res","Social","Gaming","Stream","Browse",rs,md)])
m3full<-lm(as.formula(paste("MDm~",full,"+ST+RSm")),d); cd<-cooks.distance(m3full)
f<-function(nm,dd,cv){m2<-lm(as.formula(paste("MDm~",cv,"+ST")),dd); m3<-lm(as.formula(paste("MDm~",cv,"+ST+RSm")),dd)
 g<-function(m,v){ct<-coeftest(m,vcovHC(m,"HC3"))[v,]; ci<-coefci(m,vcov.=vcovHC(m,"HC3"))[v,]; sprintf("%.3f [%.3f, %.3f], p %s",ct[1],ci[1],ci[2],ifelse(ct[4]<.001,"< .001",sprintf("= %.3f",ct[4])))}
 cat(sprintf("%s | n=%d | S2 ST: %s | S3 ST: %s | S3 RS: %s | R2 %.3f\n",nm,nrow(dd),g(m2,"ST"),g(m3,"ST"),g(m3,"RSm"),summary(m3)$r.squared))}
f("Primary",d,full)
f("No age",d,"male+mid+uni+pub+ppg+puni+urb")
f("Cook",d[cd<=4/640,],full)
f("Careless excl",d[!((sl_rs&sl_md)|mo|dupv),],full)
f("No longstring",d[!(sl_rs|sl_md),],full)
f("Minimal cov",d,"male+Age")
f("13-16",d[d$Age<=16,],"male+pub+ppg+puni+urb+Age")
f("17-19",d[d$Age>=17,],"male+pub+ppg+puni+urb+Age")
```

3 Source appendices, participant-level data panels and historic statistical tables

The following pages are reproduced without modification from the supplied Data Sheet 1. Readers using the machine-readable CSV should check the transcription and coding against these panels. The tables in Section 1 reflect the later corrected manuscript.

Supplementary Appendices, Raw Data, and Statistical Analysis

Screen Time, Reward Sensitivity, and Mood Dysregulation Among Adolescents

Data-verified research package based on the final analytic dataset ($N = 640$)

This supplementary file contains the complete research instrument package, the participant-level raw dataset, the coding key, derived-score definitions, and the recalculated statistical analyses used to support the revised manuscript.

Generated from the supplied research materials.

Contents

A Research Instrument Package **1**
A.1 Instrument Package Overview 1
A.2 Research Instrument Package 1
A.2.1 Part I. Demographic Information 1
A.2.2 Part II. Daily Screen Time Assessment 1
A.2.3 Part III. Reward Sensitivity Scale 2
A.2.4 Part IV. Mood Dysregulation Scale 2
A.3 Scoring Guide 2
A.4 Reliability Indices Recalculated From the Final Dataset 2

B Raw Data and Coding Key **4**
B.1 Dataset Structure and Derived Scores 4
B.2 Coding Key 4
B.3 Participant-Level Raw Data 4

C Recalculated Statistical Analysis **50**
C.1 Analysis Specification 50
C.2 Sample Profile 50
C.3 Reliability and Descriptive Statistics 50
C.4 Bivariate Associations 50
C.5 Group Differences 51
C.6 Three-Step Hierarchical Multiple Regression 53
C.7 Collinearity Diagnostic and Sensitivity Analysis 55
C.8 Statistical Interpretation Summary 55

A Research Instrument Package

A.1 Instrument Package Overview

Table 1: Overview of study instruments

Part	Instrument	Primary purpose	Scoring summary
I	Demographic Information Form	Describes participant background and contextual profile	Descriptive only
II	Daily Screen Time Assessment	Estimates average daily hours across major screen-based activities	Continuous total hours
III	Reward Sensitivity Scale	Assesses motivational responsiveness to rewarding stimuli	Mean of 6 items; higher scores indicate higher reward sensitivity
IV	Mood Dysregulation Scale	Assesses irritability, emotional instability, and difficulty regulating mood	Mean of 7 items; higher scores indicate greater mood dysregulation

A.2 Research Instrument Package

Instructions to participants. Please answer all sections honestly. There are no right or wrong answers. Your responses will be kept confidential and used for research purposes only.

A.2.1 Part I. Demographic Information

Instructions: Please select the option that best describes you.

Item	Response
Age	__________ years
Gender	☐ Male ☐ Female
Current Educational Level	☐ Middle School (Preparatory) ☐ High School (Secondary) ☐ University (Freshman or Junior)
School/Institution Type	☐ Public ☐ Private / International
Highest Level of Parental Education	☐ Basic / Secondary Education ☐ University Degree ☐ Postgraduate Degree (Masters/PhD)
Place of Residence	☐ Urban (City) ☐ Semi-urban / Rural

A.2.2 Part II. Daily Screen Time Assessment

Instructions: For each category below, please estimate the average number of hours you spend using a screen (smartphone, tablet, laptop, or TV) on a typical day.

Activity	Estimated hours per day
Social Media (Instagram, TikTok, Facebook, etc.)	__________ hours
Gaming (Online, Console, or Mobile games)	__________ hours
Streaming / Video (YouTube, Netflix, TV shows)	__________ hours
Browsing / Communication (Web surfing, WhatsApp)	__________ hours
**Total Daily Screen Time (Sum)**	__________ hours

A.2.3 Part III. Reward Sensitivity Scale

Instructions: Please rate how much you agree with the following statements based on your usual feelings.

Response scale: 1 = Strongly Disagree, 2 = Disagree, 3 = Agree, 4 = Strongly Agree.

No.	Statement	1	2	3	4
1	When I get something I want, I feel excited and energized.	☐	☐	☐	☐
2	When I see an opportunity for something I like, I get moving right away.	☐	☐	☐	☐
3	I often act on the spur of the moment when I see something fun to do.	☐	☐	☐	☐
4	I love the feeling of winning or being praised for my success.	☐	☐	☐	☐
5	I am always looking for new and exciting things to experience.	☐	☐	☐	☐
6	When good things happen to me, it affects me very strongly.	☐	☐	☐	☐

A.2.4 Part IV. Mood Dysregulation Scale

Instructions: Please read each statement and indicate how well it describes your behavior and feelings over the past six months.

Response scale: 1 = Not True, 2 = Somewhat True, 3 = Certainly True.

No.	Statement	1	2	3
1	I am easily annoyed by others.	☐	☐	☐
2	I often lose my temper or have "meltdowns."	☐	☐	☐
3	My mood changes very quickly without a clear reason.	☐	☐	☐
4	When I am angry or upset, it takes me a long time to calm down.	☐	☐	☐
5	I feel "on edge" or irritable most of the day.	☐	☐	☐
6	I find it difficult to control my reactions when I am frustrated.	☐	☐	☐
7	I often feel overwhelmed by my emotions.	☐	☐	☐

A.3 Scoring Guide

Scale	Scoring procedure	Interpretation
Screen Time	Calculate the continuous sum of total daily hours across all screen-based activities.	Higher values indicate greater daily screen exposure.
Reward Sensitivity	Calculate the mean score of the 6 items.	Higher scores indicate greater reward sensitivity.
Mood Dysregulation	Calculate the mean score of the 7 items.	Higher scores indicate greater mood dysregulation / irritability.

A.4 Reliability Indices Recalculated From the Final Dataset

Instrument	Cronbach's α
Screen-Time Activity Indicators	.87*
Reward Sensitivity Scale	.96
Mood Dysregulation Scale	.96

Note. Coefficients were recalculated from the final $N = 640$ dataset. The reward sensitivity and mood dysregulation scales each yielded $\alpha = .96$. *The $\alpha = .87$ coefficient across the four screen-time activity indicators is reported descriptively; total screen time is an arithmetic exposure index formed by summing heterogeneous activity categories and is not treated as a reflective psychometric scale.

B Raw Data and Coding Key

B.1 Dataset Structure and Derived Scores

The raw spreadsheet contains 640 participant-level records and 24 supplied variables. No item-level values are missing. The statistical analyses use the following derived scores:

$$\text{Total Screen Time} = ST_{\text{Social}} + ST_{\text{Gaming}} + ST_{\text{Streaming}} + ST_{\text{Browsing}},$$

$$\text{Reward Sensitivity} = \frac{RS_1 + RS_2 + RS_3 + RS_4 + RS_5 + RS_6}{6},$$

$$\text{Mood Dysregulation} = \frac{MD_1 + MD_2 + MD_3 + MD_4 + MD_5 + MD_6 + MD_7}{7}.$$

The spreadsheet coding key lists residence as a raw coded category. For the analyses below, the code is interpreted consistently with the revised manuscript: 1 = Urban and 2 = Semi-urban/Rural.

B.2 Coding Key

Table 4: Variable coding used in the raw dataset

**Variable**	**Description**	**Codes / scale**
ID	Participant ID	1–640
Age	Participant age in years	13–19
Gender	Gender	1 = Male; 2 = Female
Education	Education level	1 = Middle School; 2 = High School; 3 = University
School_Type	School type	1 = Public; 2 = Private / International
Parent_Edu	Parent education	1 = Basic/Secondary; 2 = University; 3 = Postgraduate
Residence	Residence	1 = Urban; 2 = Semi-urban/Rural
ST_Social	Screen time – social media	Raw hours per day
ST_Gaming	Screen time – gaming	Raw hours per day
ST_Streaming	Screen time – streaming/video	Raw hours per day
ST_Browsing	Screen time – browsing/communication	Raw hours per day
RS_1–RS_6	Reward Sensitivity items 1–6	Likert 1–4: Strongly Disagree to Strongly Agree
MD_1–MD_7	Mood Dysregulation items 1–7	Likert 1–3: Not True to Certainly True

B.3 Participant-Level Raw Data

For readability, the 24 raw columns are reproduced in three ID-linked panels. Together the panels contain every supplied raw value for all 640 participants.

Table 5: Raw data, Panel A: demographics and screen-time variables

ID	Age	Gend.	Edu.	School	Parent	Res.	Social	Gaming	Stream.	Browse
1	13	1	1	1	1	2	1.5	2.5	0.5	0.4
2	13	2	1	1	1	2	2.4	0.5	1.5	0.7
3	14	2	1	2	2	1	4.2	0.8	2.1	0.9
4	14	1	1	1	2	1	3.1	3.2	1.2	0.6
5	13	1	1	2	3	1	3.5	4	1.5	0.8
6	13	2	1	1	1	2	1.8	0.2	0.9	0.5
7	14	1	1	1	2	2	2.9	2.8	1.3	0.7
8	14	2	1	1	1	1	2	0.5	1.2	0.5
9	13	1	1	2	2	1	4.5	5.2	2	1.1
10	14	2	1	1	2	1	3.1	0.7	1.4	0.7
11	13	1	1	1	1	2	2.2	2.1	0.8	0.5
12	13	2	1	2	2	1	3.8	1.2	1.9	0.8
13	14	1	1	1	3	1	3.3	3.5	1.5	0.7
14	14	2	1	1	1	2	1.5	0.4	1	0.4
15	13	1	1	1	1	1	2.5	2.2	0.9	0.5
16	14	2	1	2	2	2	4	0.9	2.2	1
17	13	1	1	1	1	2	1.9	1.8	0.7	0.4
18	13	2	1	1	1	1	2.1	0.6	1.2	0.5
19	14	1	1	1	2	2	3.2	3.1	1.4	0.7
20	14	2	1	1	1	1	1.7	0.3	0.8	0.4
21	13	1	1	2	3	1	4.4	5	2.1	1
22	13	2	1	1	2	1	3	0.8	1.4	0.7
23	14	1	1	1	1	2	2.3	2.5	0.9	0.5
24	14	2	1	1	1	1	1.9	0.4	1.1	0.5
25	13	1	1	2	2	1	3.8	4.1	1.8	0.8
26	14	1	1	1	1	2	2	2.2	0.7	0.4
27	13	2	1	1	1	1	2.4	0.7	1.3	0.6
28	14	1	1	2	3	1	4.8	5.5	2.4	1.2
29	13	2	1	1	2	1	2.9	0.9	1.5	0.7
30	13	1	1	1	1	2	2.1	1.9	0.8	0.4
31	14	2	1	2	2	1	3.6	1.1	1.7	0.8
32	14	1	1	1	1	1	2.4	2.3	1	0.5
33	13	1	1	1	2	2	3.1	3	1.4	0.6
34	13	2	1	1	1	1	2.2	0.5	1.2	0.5
35	14	1	1	2	3	1	4.6	5.1	2.2	1.1
36	14	2	1	1	2	1	3.2	0.8	1.6	0.7
37	13	1	1	1	1	2	1.8	1.9	0.7	0.4
38	13	2	1	1	1	1	2	0.4	1.1	0.5
39	14	1	1	2	2	1	3.9	4.2	1.9	0.9
40	14	2	1	1	2	1	2.8	0.7	1.4	0.6
41	13	1	1	1	1	2	2.1	2	0.8	0.4
42	13	2	1	2	2	1	3.7	1	1.8	0.8
43	14	1	1	1	3	1	4.2	4.5	2	1

ID	Age	Gend.	Edu.	School	Parent	Res.	Social	Gaming	Stream.	Browse
44	14	2	1	1	1	2	1.6	0.3	1	0.4
45	13	1	1	1	1	1	2.4	2.3	1	0.5
46	14	2	1	2	2	2	4.1	1.1	2.3	1.1
47	13	1	1	1	1	2	1.9	1.8	0.7	0.4
48	13	2	1	1	1	1	2	0.4	1.1	0.5
49	14	1	1	2	3	1	4.5	5.2	2.3	1.1
50	14	2	1	1	2	1	3	0.8	1.4	0.7
51	13	1	1	1	1	2	2.2	2	0.8	0.5
52	13	2	1	2	2	1	3.8	1.1	1.9	0.9
53	14	1	1	1	3	1	4.3	4.6	2.1	1
54	14	2	1	1	1	2	1.7	0.4	1	0.4
55	13	1	1	1	1	1	2.5	2.4	1.1	0.5
56	14	2	1	2	2	2	4	1	2.2	1.1
57	13	1	1	1	1	2	2	1.9	0.8	0.4
58	13	2	1	1	1	1	1.9	0.4	1.1	0.5
59	14	1	1	2	3	1	4.6	5.3	2.4	1.2
60	14	2	1	1	2	1	3.1	0.9	1.5	0.7
61	13	1	1	1	1	2	2.3	2.2	1	0.5
62	13	2	1	2	2	1	3.7	1.2	1.8	0.9
63	14	1	1	1	3	1	4.4	4.7	2.2	1.1
64	14	2	1	1	1	2	1.8	0.5	1.1	0.5
65	13	1	1	1	1	1	2.6	2.5	1.2	0.6
66	14	2	1	2	2	2	4.1	1.1	2.3	1.1
67	13	1	1	1	1	2	2.1	2	0.9	0.4
68	13	2	1	1	1	1	2	0.5	1.2	0.5
69	14	1	1	2	3	1	4.7	5.4	2.5	1.2
70	14	2	1	1	2	1	3.2	1	1.6	0.7
71	13	1	1	1	1	2	2.4	2.3	1.1	0.5
72	13	2	1	2	2	1	3.8	1.3	1.9	1
73	14	1	1	1	3	1	4.5	4.8	2.3	1.1
74	14	2	1	1	1	2	1.9	0.6	1.2	0.5
75	13	1	1	1	1	1	2.7	2.6	1.3	0.6
76	14	2	1	2	2	2	4.2	1.2	2.4	1.2
77	13	1	1	1	1	2	2.2	2.1	1	0.5
78	13	2	1	1	1	1	2.1	0.6	1.3	0.5
79	14	1	1	2	3	1	4.8	5.5	2.6	1.3
80	14	2	1	1	2	1	3.3	1.1	1.7	0.8
81	13	1	1	1	1	2	2.5	2.4	1.2	0.6
82	13	2	1	2	2	1	3.9	1.4	2	1.1
83	14	1	1	1	3	1	4.6	4.9	2.4	1.2
84	14	2	1	1	1	2	2	0.7	1.3	0.6
85	13	1	1	1	1	1	2.8	2.7	1.4	0.7
86	14	2	1	2	2	2	4.3	1.3	2.5	1.3
87	13	1	1	1	1	2	2.3	2.2	1.1	0.5
88	13	2	1	1	1	1	2.2	0.7	1.4	0.6

ID	Age	Gend.	Edu.	School	Parent	Res.	Social	Gaming	Stream.	Browse
89	14	1	1	2	3	1	4.9	5.6	2.7	1.4
90	14	2	1	1	2	1	3.4	1.2	1.8	0.9
91	13	1	1	1	1	2	2.6	2.5	1.3	0.7
92	13	2	1	2	2	1	4	1.5	2.1	1.2
93	14	1	1	1	3	1	4.7	5	2.5	1.3
94	14	2	1	1	1	2	2.1	0.8	1.4	0.7
95	13	1	1	1	1	1	2.9	2.8	1.5	0.8
96	14	2	1	2	2	2	4.4	1.4	2.6	1.4
97	13	1	1	1	1	2	2.4	2.3	1.2	0.6
98	13	2	1	1	1	1	2.3	0.8	1.5	0.7
99	14	1	1	2	3	1	5	5.7	2.8	1.5
100	14	2	1	1	2	1	3.5	1.3	1.9	1
101	13	1	1	1	1	2	2.7	2.6	1.4	0.8
102	13	2	1	2	2	1	4.1	1.6	2.2	1.3
103	14	1	1	1	3	1	4.8	5.1	2.6	1.4
104	14	2	1	1	1	2	2.2	0.9	1.5	0.8
105	13	1	1	1	1	1	3	2.9	1.6	0.9
106	14	2	1	2	2	2	4.5	1.5	2.7	1.5
107	13	1	1	1	1	2	2.5	2.4	1.3	0.7
108	13	2	1	1	1	1	2.4	0.9	1.6	0.8
109	14	1	1	2	3	1	5.1	5.8	2.9	1.6
110	14	2	1	1	2	1	3.6	1.4	2	1.1
111	13	1	1	1	1	2	2.8	2.7	1.5	0.9
112	13	2	1	2	2	1	4.2	1.7	2.3	1.4
113	14	1	1	1	3	1	4.9	5.2	2.7	1.5
114	14	2	1	1	1	2	2.3	1	1.6	0.9
115	13	1	1	1	1	1	3.1	3	1.7	1
116	14	2	1	2	2	2	4.6	1.6	2.8	1.6
117	13	1	1	1	1	2	2.6	2.5	1.4	0.8
118	13	2	1	1	1	1	2.5	1	1.7	0.9
119	14	1	1	2	3	1	5.2	5.9	3	1.7
120	14	2	1	1	2	1	3.7	1.5	2.1	1.2
121	13	1	1	1	1	2	2.9	2.8	1.6	1
122	13	2	1	2	2	1	4.3	1.8	2.4	1.5
123	14	1	1	1	3	1	5	5.3	2.8	1.6
124	14	2	1	1	1	2	2.4	1.1	1.7	1
125	13	1	1	1	1	1	3.2	3.1	1.8	1.1
126	14	2	1	2	2	2	4.7	1.7	2.9	1.7
127	13	1	1	1	1	2	2.7	2.6	1.5	0.9
128	13	2	1	1	1	1	2.6	1.1	1.8	1
129	14	1	1	2	3	1	5.3	6	3.1	1.8
130	14	2	1	1	2	1	3.8	1.6	2.2	1.3
131	13	1	1	1	1	2	3	2.9	1.7	1.1
132	13	2	1	2	2	1	4.4	1.9	2.5	1.6
133	14	1	1	1	3	1	5.1	5.4	2.9	1.7

ID	Age	Gend.	Edu.	School	Parent	Res.	Social	Gaming	Stream.	Browse
134	14	2	1	1	1	2	2.5	1.2	1.8	1.1
135	13	1	1	1	1	1	3.3	3.2	1.9	1.2
136	14	2	1	2	2	2	4.8	1.8	3	1.8
137	13	1	1	1	1	2	2.8	2.7	1.6	1
138	13	2	1	1	1	1	2.7	1.2	1.9	1.1
139	14	1	1	2	3	1	5.4	6.1	3.2	1.9
140	14	2	1	1	2	1	3.9	1.7	2.3	1.4
141	13	1	1	1	1	2	3.1	3	1.8	1.2
142	13	2	1	2	2	1	4.5	2	2.6	1.7
143	14	1	1	1	3	1	5.2	5.5	3	1.8
144	14	2	1	1	1	2	2.6	1.3	1.9	1.2
145	13	1	1	1	1	1	3.4	3.3	2	1.3
146	14	2	1	2	2	2	4.9	1.9	3.1	1.9
147	13	1	1	1	1	2	2.9	2.8	1.7	1.1
148	13	2	1	1	1	1	2.8	1.3	2	1.2
149	14	1	1	2	3	1	5.5	6.2	3.3	2
150	14	2	1	1	2	1	4	1.8	2.4	1.5
151	13	1	1	1	1	2	3.2	3.1	1.9	1.3
152	13	2	1	2	2	1	4.6	2.1	2.7	1.8
153	14	1	1	1	3	1	5.3	5.6	3.1	1.9
154	14	2	1	1	1	2	2.7	1.4	2	1.3
155	13	1	1	1	1	1	3.5	3.4	2.1	1.4
156	14	2	1	2	2	2	5	2	3.2	2
157	13	1	1	1	1	2	3	2.9	1.8	1.2
158	13	2	1	1	1	1	2.9	1.4	2.1	1.3
159	14	1	1	2	3	1	5.6	6.3	3.4	2.1
160	14	2	1	1	2	1	4.1	1.9	2.5	1.6
161	15	1	2	1	2	1	3.3	3.5	1.5	0.7
162	15	2	2	1	1	2	4.2	0.8	2.1	0.9
163	16	2	2	2	2	1	5.1	1.1	2.5	1.2
164	16	1	2	1	2	1	3.8	4.2	1.8	0.9
165	17	2	2	1	1	2	4.5	1	2	1.1
166	17	1	2	2	3	1	3.5	3.8	1.4	0.7
167	15	1	2	1	1	1	2.8	3.1	1.2	0.6
168	16	2	2	1	2	1	4	0.9	2.2	1
169	17	1	2	2	2	1	3.2	3.5	1.4	0.7
170	15	2	2	1	1	2	3.1	0.6	1.4	0.7
171	16	1	2	1	2	2	2.9	3.2	1.3	0.7
172	17	2	2	2	3	1	5.5	1.5	2.7	1.5
173	15	1	2	1	1	2	2.5	2.8	1	0.5
174	16	2	2	1	2	1	3.8	1	1.8	0.9
175	17	1	2	2	2	1	4.3	4.6	2.1	1
176	15	2	2	1	1	1	2.4	0.5	1.2	0.6
177	16	1	2	1	2	2	1.9	2.2	0.8	0.4
178	17	2	2	2	3	1	4.9	1.4	2.5	1.3

ID	Age	Gend.	Edu.	School	Parent	Res.	Social	Gaming	Stream.	Browse
179	15	1	2	1	1	2	2.3	2.5	1	0.5
180	16	2	2	1	2	1	3.4	0.8	1.6	0.8
181	17	1	2	2	2	1	4.1	4.4	2	1
182	15	2	2	1	1	1	2	0.4	1.1	0.5
183	16	1	2	1	2	2	3.1	3.4	1.4	0.7
184	17	2	2	2	3	1	5.2	1.5	2.6	1.4
185	15	1	2	1	1	2	2.2	2.5	1	0.5
186	16	2	2	1	2	1	3.5	0.9	1.7	0.8
187	17	1	2	2	2	1	4.4	4.7	2.1	1.1
188	15	2	2	1	1	1	2.5	0.6	1.3	0.6
189	16	1	2	1	2	2	2	2.3	0.9	0.4
190	17	2	2	2	3	1	5	1.4	2.5	1.3
191	15	1	2	1	1	2	2.4	2.6	1.1	0.5
192	16	2	2	1	2	1	3.6	1	1.8	0.9
193	17	1	2	2	2	1	4.2	4.5	2	1
194	15	2	2	1	1	1	2.1	0.4	1.1	0.5
195	16	1	2	1	2	2	3.2	3.5	1.4	0.7
196	17	2	2	2	3	1	5.3	1.6	2.7	1.5
197	15	1	2	1	1	2	2.3	2.6	1.1	0.5
198	16	2	2	1	2	1	3.7	0.9	1.8	0.9
199	17	1	2	2	2	1	4.5	4.8	2.2	1.1
200	15	2	2	1	1	1	2.6	0.6	1.4	0.6
201	16	1	2	1	2	2	2.1	2.4	0.9	0.5
202	17	2	2	2	3	1	5.1	1.5	2.6	1.4
203	15	1	2	1	1	2	2.5	2.8	1.2	0.6
204	16	2	2	1	2	1	3.7	1	1.9	0.9
205	17	1	2	2	2	1	4.3	4.6	2.1	1.1
206	15	2	2	1	1	1	2.2	0.5	1.2	0.5
207	16	1	2	1	2	2	3.3	3.6	1.5	0.7
208	17	2	2	2	3	1	5.4	1.7	2.8	1.6
209	15	1	2	1	1	2	2.4	2.7	1.1	0.5
210	16	2	2	1	2	1	3.8	1	1.9	1
211	17	1	2	2	2	1	4.4	4.7	2.2	1.1
212	15	2	2	1	1	1	2.7	0.7	1.4	0.7
213	16	1	2	1	2	2	2.2	2.5	1	0.5
214	17	2	2	2	3	1	5.2	1.6	2.7	1.5
215	15	1	2	1	1	2	2.6	2.9	1.3	0.7
216	16	2	2	1	2	1	3.8	1.1	2	1
217	17	1	2	2	2	1	4.4	4.7	2.3	1.1
218	15	2	2	1	1	1	2.3	0.6	1.3	0.6
219	16	1	2	1	2	2	3.4	3.7	1.6	0.8
220	17	2	2	2	3	1	5.5	1.8	2.9	1.7
221	15	1	2	1	1	2	2.5	2.8	1.2	0.6
222	16	2	2	1	2	1	3.9	1.1	2	1.1
223	17	1	2	2	2	1	4.5	4.8	2.3	1.2

ID	Age	Gend.	Edu.	School	Parent	Res.	Social	Gaming	Stream.	Browse
224	15	2	2	1	1	1	2.8	0.8	1.5	0.7
225	16	1	2	1	2	2	2.3	2.6	1.1	0.5
226	17	2	2	2	3	1	5.3	1.7	2.8	1.6
227	15	1	2	1	1	2	2.7	3	1.4	0.8
228	16	2	2	1	2	1	3.9	1.2	2.1	1.1
229	17	1	2	2	2	1	4.5	4.8	2.4	1.2
230	15	2	2	1	1	1	2.4	0.7	1.4	0.7
231	16	1	2	1	2	2	3.5	3.8	1.7	0.9
232	17	2	2	2	3	1	5.6	1.9	3	1.8
233	15	1	2	1	1	2	2.6	2.9	1.3	0.7
234	16	2	2	1	2	1	4	1.2	2.1	1.2
235	17	1	2	2	2	1	4.6	4.9	2.4	1.3
236	15	2	2	1	1	1	2.9	0.9	1.6	0.8
237	16	1	2	1	2	2	2.4	2.7	1.2	0.6
238	17	2	2	2	3	1	5.4	1.8	2.9	1.7
239	15	1	2	1	1	2	2.8	3.1	1.5	0.9
240	16	2	2	1	2	1	4	1.3	2.2	1.2
241	17	1	2	2	2	1	4.6	4.9	2.5	1.3
242	15	2	2	1	1	1	2.5	0.8	1.5	0.8
243	16	1	2	1	2	2	3.6	3.9	1.8	1
244	17	2	2	2	3	1	5.7	2	3.1	1.9
245	15	1	2	1	1	2	2.7	3	1.4	0.8
246	16	2	2	1	2	1	4.1	1.3	2.2	1.3
247	17	1	2	2	2	1	4.7	5	2.5	1.4
248	15	2	2	1	1	1	3	1	1.7	0.9
249	16	1	2	1	2	2	2.5	2.8	1.3	0.7
250	17	2	2	2	3	1	5.5	1.9	3	1.8
251	15	1	2	1	1	2	2.9	3.2	1.6	1
252	16	2	2	1	2	1	4.1	1.4	2.3	1.3
253	17	1	2	2	2	1	4.7	5	2.6	1.4
254	15	2	2	1	1	1	2.6	0.9	1.6	0.9
255	16	1	2	1	2	2	3.7	4	1.9	1.1
256	17	2	2	2	3	1	5.8	2.1	3.2	2
257	15	1	2	1	1	2	2.8	3.1	1.5	0.9
258	16	2	2	1	2	1	4.2	1.4	2.3	1.4
259	17	1	2	2	2	1	4.8	5.1	2.6	1.5
260	15	2	2	1	1	1	3.1	1.1	1.8	1
261	16	1	2	1	2	2	2.6	2.9	1.4	0.8
262	17	2	2	2	3	1	5.6	2	3.1	1.9
263	15	1	2	1	1	2	3	3.3	1.7	1.1
264	16	2	2	1	2	1	4.2	1.5	2.4	1.4
265	17	1	2	2	2	1	4.8	5.1	2.7	1.5
266	15	2	2	1	1	1	2.7	1	1.7	1
267	16	1	2	1	2	2	3.8	4.1	2	1.2
268	17	2	2	2	3	1	5.9	2.2	3.3	2.1

ID	Age	Gend.	Edu.	School	Parent	Res.	Social	Gaming	Stream.	Browse
269	15	1	2	1	1	2	2.9	3.2	1.6	1
270	16	2	2	1	2	1	4.3	1.5	2.4	1.5
271	17	1	2	2	2	1	4.9	5.2	2.7	1.6
272	15	2	2	1	1	1	3.2	1.2	1.9	1.1
273	16	1	2	1	2	2	2.7	3	1.5	0.9
274	17	2	2	2	3	1	5.7	2.1	3.2	2
275	15	1	2	1	1	2	3.1	3.4	1.8	1.2
276	16	2	2	1	2	1	4.3	1.6	2.5	1.5
277	17	1	2	2	2	1	4.9	5.2	2.8	1.6
278	15	2	2	1	1	1	2.8	1.1	1.8	1.1
279	16	1	2	1	2	2	3.9	4.2	2.1	1.3
280	17	2	2	2	3	1	6	2.3	3.4	2.2
281	15	1	2	1	1	2	3	3.3	1.7	1.1
282	16	2	2	1	2	1	4.4	1.6	2.5	1.6
283	17	1	2	2	2	1	5	5.3	2.8	1.7
284	15	2	2	1	1	1	3.3	1.3	2	1.2
285	16	1	2	1	2	2	2.8	3.1	1.6	1
286	17	2	2	2	3	1	5.8	2.2	3.3	2.1
287	15	1	2	1	1	2	3.2	3.5	1.9	1.3
288	16	2	2	1	2	1	4.4	1.7	2.6	1.6
289	17	1	2	2	2	1	5	5.3	2.9	1.7
290	15	2	2	1	1	1	2.9	1.2	1.9	1.2
291	16	1	2	1	2	2	4	4.3	2.2	1.4
292	17	2	2	2	3	1	6.1	2.4	3.5	2.3
293	15	1	2	1	1	2	3.1	3.4	1.8	1.2
294	16	2	2	1	2	1	4.5	1.7	2.6	1.7
295	17	1	2	2	2	1	5.1	5.4	2.9	1.8
296	15	2	2	1	1	1	3.4	1.4	2.1	1.3
297	16	1	2	1	2	2	2.9	3.2	1.7	1.1
298	17	2	2	2	3	1	5.9	2.3	3.4	2.2
299	15	1	2	1	1	2	3.3	3.6	2	1.4
300	16	2	2	1	2	1	4.5	1.8	2.7	1.7
301	17	1	2	2	2	1	5.1	5.4	3	1.8
302	15	2	2	1	1	1	3	1.3	2	1.3
303	16	1	2	1	2	2	4.1	4.4	2.3	1.5
304	17	2	2	2	3	1	6.2	2.5	3.6	2.4
305	15	1	2	1	1	2	3.2	3.5	1.9	1.3
306	16	2	2	1	2	1	4.6	1.8	2.7	1.8
307	17	1	2	2	2	1	5.2	5.5	3	1.9
308	15	2	2	1	1	1	3.5	1.5	2.2	1.4
309	16	1	2	1	2	2	3	3.3	1.8	1.2
310	17	2	2	2	3	1	6	2.4	3.5	2.3
311	15	1	2	1	1	2	3.4	3.7	2.1	1.5
312	16	2	2	1	2	1	4.6	1.9	2.8	1.8
313	17	1	2	2	2	1	5.2	5.5	3.1	1.9

ID	Age	Gend.	Edu.	School	Parent	Res.	Social	Gaming	Stream.	Browse
314	15	2	2	1	1	1	3.1	1.3	2	1.3
315	16	1	2	1	2	2	4.2	4.5	2.4	1.6
316	17	2	2	2	3	1	6.3	2.6	3.7	2.5
317	15	1	2	1	1	2	3.3	3.6	2	1.4
318	16	2	2	1	2	1	4.7	1.9	2.8	1.9
319	17	1	2	2	2	1	5.3	5.6	3.1	2
320	15	2	2	1	1	1	3.6	1.6	2.3	1.5
321	16	1	2	1	2	2	3.1	3.4	1.9	1.3
322	17	2	2	2	3	1	6.1	2.5	3.6	2.4
323	15	1	2	1	1	2	3.5	3.8	2.2	1.6
324	16	2	2	1	2	1	4.7	2	2.9	1.9
325	17	1	2	2	2	1	5.3	5.6	3.2	2
326	15	2	2	1	1	1	3.2	1.4	2.1	1.4
327	16	1	2	1	2	2	4.3	4.6	2.5	1.7
328	17	2	2	2	3	1	6.4	2.7	3.8	2.6
329	15	1	2	1	1	2	3.4	3.7	2.1	1.5
330	16	2	2	1	2	1	4.8	2	2.9	2
331	17	1	2	2	2	1	5.4	5.7	3.2	2.1
332	15	2	2	1	1	1	3.7	1.7	2.4	1.6
333	16	1	2	1	2	2	3.2	3.5	2	1.4
334	17	2	2	2	3	1	6.2	2.6	3.7	2.5
335	15	1	2	1	1	2	3.6	3.9	2.3	1.7
336	16	2	2	1	2	1	4.8	2.1	3	2
337	17	1	2	2	2	1	5.4	5.7	3.3	2.1
338	15	2	2	1	1	1	3.3	1.5	2.2	1.5
339	16	1	2	1	2	2	4.4	4.7	2.6	1.8
340	17	2	2	2	3	1	6.5	2.8	3.9	2.7
341	15	1	2	1	1	2	3.5	3.8	2.2	1.6
342	16	2	2	1	2	1	4.9	2.1	3	2.1
343	17	1	2	2	2	1	5.5	5.8	3.3	2.2
344	15	2	2	1	1	1	3.8	1.8	2.5	1.7
345	16	1	2	1	2	2	3.3	3.6	2.1	1.5
346	17	2	2	2	3	1	6.3	2.7	3.8	2.6
347	15	1	2	1	1	2	3.7	4	2.4	1.8
348	16	2	2	1	2	1	4.9	2.2	3.1	2.1
349	17	1	2	2	2	1	5.5	5.8	3.4	2.2
350	15	2	2	1	1	1	3.4	1.6	2.3	1.6
351	16	1	2	1	2	2	4.5	4.8	2.7	1.9
352	17	2	2	2	3	1	6.6	2.9	4	2.8
353	15	1	2	1	1	2	3.6	3.9	2.3	1.7
354	16	2	2	1	2	1	5	2.2	3.1	2.2
355	17	1	2	2	2	1	5.6	5.9	3.4	2.3
356	15	2	2	1	1	1	3.9	1.9	2.6	1.8
357	16	1	2	1	2	2	3.4	3.7	2.2	1.6
358	17	2	2	2	3	1	6.4	2.8	3.9	2.7

ID	Age	Gend.	Edu.	School	Parent	Res.	Social	Gaming	Stream.	Browse
359	15	1	2	1	1	2	3.8	4.1	2.5	1.9
360	16	2	2	1	2	1	5	2.3	3.2	2.2
361	17	1	2	2	2	1	5.6	5.9	3.5	2.3
362	15	2	2	1	1	1	3.5	1.7	2.4	1.7
363	16	1	2	1	2	2	4.6	4.9	2.8	2
364	17	2	2	2	3	1	6.7	3	4.1	2.9
365	15	1	2	1	1	2	3.7	4	2.4	1.8
366	16	2	2	1	2	1	5.1	2.3	3.2	2.3
367	17	1	2	2	2	1	5.7	6	3.5	2.4
368	15	2	2	1	1	1	4	2	2.7	1.9
369	16	1	2	1	2	2	3.5	3.8	2.3	1.7
370	17	2	2	2	3	1	6.5	2.9	4	2.8
371	15	1	2	1	1	2	3.9	4.2	2.6	2
372	16	2	2	1	2	1	5.1	2.4	3.3	2.3
373	17	1	2	2	2	1	5.7	6	3.6	2.4
374	15	2	2	1	1	1	3.6	1.8	2.5	1.8
375	16	1	2	1	2	2	4.7	5	2.9	2.1
376	17	2	2	2	3	1	6.8	3.1	4.2	3
377	15	1	2	1	1	2	3.8	4.1	2.5	1.9
378	16	2	2	1	2	1	5.2	2.4	3.3	2.4
379	17	1	2	2	2	1	5.8	6.1	3.6	2.5
380	15	2	2	1	1	1	4.1	2.1	2.8	2
381	16	1	2	1	2	2	3.6	3.9	2.4	1.8
382	17	2	2	2	3	1	6.6	3	4.1	2.9
383	15	1	2	1	1	2	4	4.3	2.7	2.1
384	16	2	2	1	2	1	5.2	2.5	3.4	2.4
385	17	1	2	2	2	1	5.8	6.1	3.7	2.5
386	15	2	2	1	1	1	3.7	1.9	2.6	1.9
387	16	1	2	1	2	2	4.8	5.1	3	2.2
388	17	2	2	2	3	1	6.9	3.2	4.3	3.1
389	15	1	2	1	1	2	3.9	4.2	2.6	2
390	16	2	2	1	2	1	5.3	2.5	3.4	2.5
391	17	1	2	2	2	1	5.9	6.2	3.7	2.6
392	15	2	2	1	1	1	4.2	2.2	2.9	2.1
393	16	1	2	1	2	2	3.7	4	2.5	1.9
394	17	2	2	2	3	1	6.7	3.1	4.2	3
395	15	1	2	1	1	2	4.1	4.4	2.8	2.2
396	16	2	2	1	2	1	5.3	2.6	3.5	2.5
397	17	1	2	2	2	1	5.9	6.2	3.8	2.6
398	15	2	2	1	1	1	3.8	2	2.7	2
399	16	1	2	1	2	2	4.9	5.2	3.1	2.3
400	17	2	2	2	3	1	7	3.3	4.4	3.2
401	15	1	2	1	1	2	4	4.3	2.7	2.1
402	16	2	2	1	2	1	5.4	2.6	3.5	2.6
403	17	1	2	2	2	1	6	6.3	3.8	2.7

ID	Age	Gend.	Edu.	School	Parent	Res.	Social	Gaming	Stream.	Browse
404	15	2	2	1	1	1	4.3	2.3	3	2.2
405	16	1	2	1	2	2	3.8	4.1	2.6	2
406	17	2	2	2	3	1	6.8	3.2	4.3	3.1
407	15	1	2	1	1	2	4.2	4.5	2.9	2.3
408	16	2	2	1	2	1	5.4	2.7	3.6	2.6
409	17	1	2	2	2	1	6	6.3	3.9	2.7
410	15	2	2	1	1	1	3.9	2.1	2.8	2.1
411	16	1	2	1	2	2	5	5.3	3.2	2.4
412	17	2	2	2	3	1	7.1	3.4	4.5	3.3
413	15	1	2	1	1	2	4.1	4.4	2.8	2.2
414	16	2	2	1	2	1	5.5	2.7	3.6	2.7
415	17	1	2	2	2	1	6.1	6.4	3.9	2.8
416	15	2	2	1	1	1	4.4	2.4	3.1	2.3
417	16	1	2	1	2	2	3.9	4.2	2.7	2.1
418	17	2	2	2	3	1	6.9	3.3	4.4	3.2
419	15	1	2	1	1	2	4.3	4.6	3	2.4
420	16	2	2	1	2	1	5.5	2.8	3.7	2.7
421	17	1	2	2	2	1	6.1	6.4	4	2.8
422	15	2	2	1	1	1	4	2.2	2.9	2.2
423	16	1	2	1	2	2	5.1	5.4	3.3	2.5
424	17	2	2	2	3	1	7.2	3.5	4.6	3.4
425	15	1	2	1	1	2	4.2	4.5	2.9	2.3
426	16	2	2	1	2	1	5.6	2.8	3.7	2.8
427	17	1	2	2	2	1	6.2	6.5	4	2.9
428	15	2	2	1	1	1	4.5	2.5	3.2	2.4
429	16	1	2	1	2	2	4	4.3	2.8	2.2
430	17	2	2	2	3	1	7	3.4	4.5	3.3
431	15	1	2	1	1	2	4.4	4.7	3.1	2.5
432	16	2	2	1	2	1	5.6	2.9	3.8	2.8
433	17	1	2	2	2	1	6.2	6.5	4.1	2.9
434	15	2	2	1	1	1	4.1	2.3	3	2.3
435	16	1	2	1	2	2	5.2	5.5	3.4	2.6
436	17	2	2	2	3	1	7.3	3.6	4.7	3.5
437	15	1	2	1	1	2	4.3	4.6	3	2.4
438	16	2	2	1	2	1	5.7	2.9	3.8	2.9
439	17	1	2	2	2	1	6.3	6.6	4.1	3
440	15	2	2	1	1	1	4.6	2.6	3.3	2.5
441	16	1	2	1	2	2	4.1	4.4	2.9	2.3
442	17	2	2	2	3	1	7.1	3.5	4.6	3.4
443	15	1	2	1	1	2	4.5	4.8	3.2	2.6
444	16	2	2	1	2	1	5.7	3	3.9	2.9
445	17	1	2	2	2	1	6.3	6.6	4.2	3
446	15	2	2	1	1	1	4.2	2.4	3.1	2.4
447	16	1	2	1	2	2	5.3	5.6	3.5	2.7
448	17	2	2	2	3	1	7.4	3.7	4.8	3.6

ID	Age	Gend.	Edu.	School	Parent	Res.	Social	Gaming	Stream.	Browse
449	18	1	3	1	2	1	2.9	2.8	1.4	0.7
450	19	2	3	2	1	1	2.4	0.5	1.1	0.6
451	18	1	3	1	2	1	3	2.1	1.4	0.8
452	19	2	3	2	2	1	3.7	2.2	1.8	0.9
453	18	1	3	1	1	1	2.3	1.3	1.1	0.6
454	19	1	3	2	1	1	2.3	0.5	1.1	0.6
455	18	1	3	1	2	1	3.4	2.5	1.5	0.9
456	19	2	3	2	2	1	3.1	1.9	1.4	0.7
457	18	2	3	2	2	1	2	0.4	1	0.5
458	19	1	3	2	2	1	3	1.3	1.3	0.7
459	18	2	3	1	2	1	2.1	1.5	1	0.5
460	19	1	3	2	2	1	3.6	1.4	1.8	1.1
461	18	2	3	1	1	2	2	0.5	0.9	0.5
462	19	2	3	2	1	1	1.4	0.1	0.7	0.3
463	18	2	3	1	1	1	1.9	0.4	0.9	0.4
464	19	1	3	1	2	2	2.3	0.9	1.1	0.6
465	18	1	3	1	1	1	2	0.4	1	0.5
466	19	2	3	2	2	1	3.5	1.6	1.7	0.8
467	18	1	3	1	2	1	1.7	1	0.9	0.4
468	19	1	3	1	1	1	1.1	0.6	0.6	0.3
469	18	2	3	2	2	1	3.2	1.3	1.6	0.8
470	19	2	3	1	1	2	1.8	0.7	0.9	0.4
471	18	1	3	2	2	1	4	2.1	2	1
472	19	2	3	2	1	1	2.3	1.1	1.1	0.6
473	18	1	3	1	2	1	3.5	2.6	1.7	0.9
474	19	1	3	2	3	1	4.1	2.2	2	1
475	18	2	3	1	1	1	2.2	1	1.1	0.6
476	19	1	3	1	2	2	1.7	0.8	0.8	0.4
477	18	2	3	2	3	1	5	2.3	2.5	1.2
478	19	1	3	1	1	2	2.1	1.1	1	0.5
479	18	2	3	1	2	1	3.2	1.5	1.6	0.8
480	19	1	3	2	2	1	3.9	1.8	1.9	1
481	18	2	3	1	1	1	1.8	0.8	0.9	0.4
482	19	1	3	1	2	2	3	1.6	1.4	0.7
483	18	2	3	2	3	1	5.2	2.4	2.6	1.4
484	19	1	3	1	1	2	2.1	1.1	1	0.5
485	18	2	3	1	2	1	3.3	1.6	1.6	0.8
486	19	1	3	2	2	1	4.2	1.9	2.1	1.1
487	18	2	3	1	1	1	2.3	1.1	1.1	0.6
488	19	1	3	1	2	2	1.8	0.9	0.9	0.4
489	18	2	3	2	3	1	4.8	2.2	2.4	1.3
490	19	1	3	1	1	2	2.3	1.2	1.1	0.5
491	18	2	3	1	2	1	3.4	1.7	1.7	0.9
492	19	1	3	2	2	1	4.1	1.9	2	1
493	18	2	3	1	1	1	2.1	1	1	0.5

ID	Age	Gend.	Edu.	School	Parent	Res.	Social	Gaming	Stream.	Browse
494	19	1	3	1	2	2	3.1	1.7	1.5	0.7
495	18	2	3	2	3	1	5.3	2.5	2.6	1.4
496	19	1	3	1	1	2	2.3	1.2	1.1	0.5
497	18	2	3	1	2	1	3.5	1.7	1.7	0.9
498	19	1	3	2	2	1	4.3	2	2.1	1.1
499	18	2	3	1	1	1	2.5	1.2	1.2	0.6
500	19	1	3	1	2	2	2	1	1	0.5
501	18	2	3	2	3	1	4.9	2.3	2.4	1.3
502	19	1	3	1	1	2	2.4	1.2	1.2	0.6
503	18	2	3	1	2	1	3.5	1.7	1.8	0.9
504	19	1	3	2	2	1	4.2	2	2.1	1
505	18	2	3	1	1	1	2	1	1	0.5
506	19	1	3	1	2	2	3.1	1.7	1.5	0.7
507	18	2	3	2	3	1	5.3	2.5	2.6	1.4
508	19	1	3	1	1	2	2.4	1.2	1.2	0.6
509	18	2	3	1	2	1	3.6	1.8	1.8	0.9
510	19	1	3	2	2	1	4.3	2.1	2.1	1.1
511	18	2	3	1	1	1	2.2	1.1	1.1	0.5
512	19	1	3	1	2	2	2.8	1.5	1.4	0.7
513	18	2	3	2	3	1	5	2.4	2.5	1.3
514	19	1	3	1	1	2	2.4	1.3	1.2	0.6
515	18	2	3	1	2	1	3.5	1.8	1.7	0.9
516	19	1	3	2	2	1	4.4	2.2	2.2	1.1
517	18	2	3	1	1	1	2.1	1.1	1	0.5
518	19	1	3	1	2	2	3.3	1.8	1.6	0.8
519	18	2	3	2	3	1	5.4	2.6	2.7	1.4
520	19	1	3	1	1	2	2.5	1.3	1.2	0.6
521	18	2	3	1	2	1	3.7	1.9	1.9	0.9
522	19	1	3	2	2	1	4.4	2.2	2.2	1.1
523	18	2	3	1	1	1	2.3	1.2	1.1	0.6
524	19	1	3	1	2	2	2.9	1.6	1.4	0.7
525	18	2	3	2	3	1	5.1	2.5	2.6	1.4
526	19	1	3	1	1	2	2.5	1.4	1.2	0.6
527	18	2	3	1	2	1	3.6	1.9	1.8	0.9
528	19	1	3	2	2	1	4.5	2.3	2.3	1.2
529	18	2	3	1	1	1	2.2	1.2	1.1	0.6
530	19	1	3	1	2	2	3.4	1.9	1.7	0.8
531	18	2	3	2	3	1	5.5	2.7	2.8	1.5
532	19	1	3	1	1	2	2.6	1.4	1.3	0.7
533	18	2	3	1	2	1	3.8	2	1.9	1
534	19	1	3	2	2	1	4.5	2.3	2.3	1.2
535	18	2	3	1	1	1	2.4	1.3	1.2	0.6
536	19	1	3	1	2	2	3	1.7	1.5	0.8
537	18	2	3	2	3	1	5.2	2.6	2.6	1.4
538	19	1	3	1	1	2	2.6	1.5	1.3	0.7

ID	Age	Gend.	Edu.	School	Parent	Res.	Social	Gaming	Stream.	Browse
539	18	2	3	1	2	1	3.7	1.9	1.9	1
540	19	1	3	2	2	1	4.6	2.4	2.4	1.2
541	18	2	3	1	1	1	2.3	1.2	1.2	0.6
542	19	1	3	1	2	2	3.5	2	1.7	0.9
543	18	2	3	2	3	1	5.6	2.8	2.9	1.5
544	19	1	3	1	1	2	2.7	1.5	1.4	0.7
545	18	2	3	1	2	1	3.9	2.1	2	1
546	19	1	3	2	2	1	4.6	2.4	2.4	1.3
547	18	2	3	1	1	1	2.5	1.4	1.2	0.7
548	19	1	3	1	2	2	3.1	1.8	1.6	0.8
549	18	2	3	2	3	1	5.3	2.7	2.7	1.5
550	19	1	3	1	1	2	2.7	1.6	1.4	0.8
551	18	2	3	1	2	1	3.8	2	1.9	1
552	19	1	3	2	2	1	4.7	2.5	2.5	1.3
553	18	2	3	1	1	1	2.4	1.3	1.2	0.7
554	19	1	3	1	2	2	3.6	2.1	1.8	1
555	18	2	3	2	3	1	5.7	2.9	3	1.6
556	19	1	3	1	1	2	2.8	1.6	1.4	0.8
557	18	2	3	1	2	1	4	2.2	2.1	1.1
558	19	1	3	2	2	1	4.7	2.5	2.5	1.3
559	18	2	3	1	1	1	2.6	1.5	1.3	0.7
560	19	1	3	1	2	2	3.2	1.9	1.7	0.9
561	18	2	3	2	3	1	5.4	2.8	2.8	1.6
562	19	1	3	1	1	2	2.8	1.7	1.5	0.8
563	18	2	3	1	2	1	3.9	2.1	2	1.1
564	19	1	3	2	2	1	4.8	2.6	2.6	1.4
565	18	2	3	1	1	1	2.5	1.4	1.3	0.7
566	19	1	3	1	2	2	3.7	2.2	1.9	1.1
567	18	2	3	2	3	1	5.8	3	3.1	1.7
568	19	1	3	1	1	2	2.9	1.7	1.5	0.9
569	18	2	3	1	2	1	4.1	2.3	2.2	1.2
570	19	1	3	2	2	1	4.8	2.6	2.6	1.4
571	18	2	3	1	1	1	2.7	1.6	1.4	0.8
572	19	1	3	1	2	2	3.3	2	1.8	1
573	18	2	3	2	3	1	5.5	2.9	2.9	1.7
574	19	1	3	1	1	2	2.9	1.8	1.6	0.9
575	18	2	3	1	2	1	4	2.2	2.1	1.1
576	19	1	3	2	2	1	4.9	2.7	2.7	1.5
577	18	2	3	1	1	1	2.6	1.5	1.4	0.8
578	19	1	3	1	2	2	3.8	2.3	2	1.2
579	18	2	3	2	3	1	5.9	3.1	3.2	1.8
580	19	1	3	1	1	2	3	1.8	1.6	1
581	18	2	3	1	2	1	4.2	2.4	2.3	1.3
582	19	1	3	2	2	1	4.9	2.7	2.7	1.5
583	18	2	3	1	1	1	2.8	1.7	1.5	0.9

ID	Age	Gend.	Edu.	School	Parent	Res.	Social	Gaming	Stream.	Browse
584	19	1	3	1	2	2	3.4	2.1	1.9	1.1
585	18	2	3	2	3	1	5.6	3	3	1.8
586	19	1	3	1	1	2	3	1.9	1.7	1
587	18	2	3	1	2	1	4.1	2.3	2.2	1.2
588	19	1	3	2	2	1	5	2.8	2.8	1.6
589	18	2	3	1	1	1	2.7	1.6	1.5	0.9
590	19	1	3	1	2	2	3.9	2.4	2.1	1.3
591	18	2	3	2	3	1	6	3.2	3.3	1.9
592	19	1	3	1	1	2	3.1	1.9	1.7	1.1
593	18	2	3	1	2	1	4.3	2.5	2.4	1.4
594	19	1	3	2	2	1	5	2.8	2.8	1.6
595	18	2	3	1	1	1	2.9	1.8	1.6	1
596	19	1	3	1	2	2	3.5	2.2	2	1.2
597	18	2	3	2	3	1	5.7	3.1	3.1	1.9
598	19	1	3	1	1	2	3.1	2	1.8	1.1
599	18	2	3	1	2	1	4.2	2.4	2.3	1.3
600	19	1	3	2	2	1	5.1	2.9	2.9	1.7
601	18	2	3	1	1	1	2.8	1.7	1.6	1
602	19	1	3	1	2	2	4	2.5	2.2	1.4
603	18	2	3	2	3	1	6.1	3.3	3.4	2
604	19	1	3	1	1	2	3.2	2	1.8	1.2
605	18	2	3	1	2	1	4.4	2.6	2.5	1.5
606	19	1	3	2	2	1	5.1	2.9	2.9	1.7
607	18	2	3	1	1	1	3	1.9	1.7	1.1
608	19	1	3	1	2	2	3.6	2.3	2.1	1.3
609	18	2	3	2	3	1	5.8	3.2	3.2	2
610	19	1	3	1	1	2	3.2	2.1	1.9	1.2
611	18	2	3	1	2	1	4.3	2.5	2.4	1.4
612	19	1	3	2	2	1	5.2	3	3	1.8
613	18	2	3	1	1	1	2.9	1.8	1.7	1.1
614	19	1	3	1	2	2	4.1	2.6	2.3	1.5
615	18	2	3	2	3	1	6.2	3.4	3.5	2.1
616	19	1	3	1	1	2	3.3	2.1	1.9	1.3
617	18	2	3	1	2	1	4.5	2.7	2.6	1.6
618	19	1	3	2	2	1	5.2	3	3	1.8
619	18	2	3	1	1	1	3.1	2	1.8	1.2
620	19	1	3	1	2	2	3.7	2.4	2.2	1.4
621	18	2	3	2	3	1	5.9	3.3	3.3	2.1
622	19	1	3	1	1	2	3.3	2.2	2	1.3
623	18	2	3	1	2	1	4.4	2.6	2.5	1.5
624	19	1	3	2	2	1	5.3	3.1	3.1	1.9
625	18	2	3	1	1	1	3	1.9	1.8	1.2
626	19	1	3	1	2	2	4.2	2.7	2.4	1.6
627	18	2	3	2	3	1	6.3	3.5	3.6	2.2
628	19	1	3	1	1	2	3.4	2.2	2	1.4

ID	Age	Gend.	Edu.	School	Parent	Res.	Social	Gaming	Stream.	Browse
629	18	2	3	1	2	1	4.6	2.8	2.7	1.7
630	19	1	3	2	2	1	5.3	3.1	3.1	1.9
631	18	2	3	1	1	1	3.2	2.1	1.9	1.3
632	19	1	3	1	2	2	3.8	2.5	2.3	1.5
633	18	2	3	2	3	1	6	3.4	3.4	2.2
634	19	1	3	1	1	2	3.4	2.3	2.1	1.4
635	18	2	3	1	2	1	4.5	2.7	2.6	1.6
636	19	1	3	2	2	1	5.4	3.2	3.2	2
637	18	2	3	1	1	1	3.1	2	1.9	1.3
638	19	1	3	1	2	2	4.3	2.8	2.5	1.7
639	18	2	3	2	3	1	6.4	3.6	3.7	2.3
640	19	1	3	1	1	2	3.5	2.3	2.1	1.5

Table 6: Raw data, Panel B: Reward Sensitivity items

ID	RS1	RS2	RS3	RS4	RS5	RS6
1	2	2	3	2	2	2
2	3	2	3	2	3	2
3	4	3	4	4	4	3
4	3	3	3	3	3	3
5	4	4	4	4	4	4
6	2	2	2	2	3	2
7	3	3	3	3	4	3
8	2	2	3	2	2	2
9	4	4	3	4	4	4
10	3	3	3	3	3	3
11	2	3	2	2	3	2
12	4	3	4	4	4	3
13	3	4	3	4	4	3
14	2	2	2	2	2	2
15	3	2	2	3	3	2
16	4	4	4	4	4	4
17	2	2	3	2	3	2
18	3	2	3	3	3	2
19	3	3	4	3	4	3
20	2	2	2	2	2	2
21	4	4	4	4	4	4
22	3	3	3	3	3	3
23	3	2	2	3	3	2
24	2	3	2	2	2	2
25	4	3	4	4	4	3
26	2	2	2	2	3	2
27	3	3	2	3	3	2
28	4	4	4	4	4	4
29	3	3	3	3	4	3
30	2	3	2	2	3	2
31	4	4	4	4	4	4
32	3	2	3	3	3	2
33	3	3	3	3	4	3
34	3	3	2	3	3	2
35	4	4	4	4	4	4
36	3	3	3	3	3	3
37	2	2	2	2	3	2
38	2	2	2	2	2	2
39	4	4	3	4	4	4
40	3	3	3	3	4	3
41	2	3	2	2	3	2
42	4	3	4	4	4	4
43	4	4	4	4	4	4

ID	RS1	RS2	RS3	RS4	RS5	RS6
44	2	2	2	2	2	2
45	3	2	2	3	3	2
46	4	4	4	4	4	4
47	2	2	3	2	3	2
48	3	2	3	3	3	2
49	4	4	4	4	4	4
50	3	3	3	3	3	3
51	2	3	2	2	3	2
52	4	3	4	4	4	4
53	4	4	4	4	4	4
54	2	2	2	2	2	2
55	3	2	2	3	3	2
56	4	4	4	4	4	4
57	2	2	3	2	3	2
58	3	2	3	3	3	2
59	4	4	4	4	4	4
60	3	3	3	3	3	3
61	2	3	2	2	3	2
62	4	3	4	4	4	4
63	4	4	4	4	4	4
64	2	2	2	2	2	2
65	3	2	2	3	3	2
66	4	4	4	4	4	4
67	2	2	3	2	3	2
68	3	2	3	3	3	2
69	4	4	4	4	4	4
70	3	3	3	3	3	3
71	2	3	2	2	3	2
72	4	3	4	4	4	4
73	4	4	4	4	4	4
74	2	2	2	2	2	2
75	3	2	2	3	3	2
76	4	4	4	4	4	4
77	2	2	3	2	3	2
78	3	2	3	3	3	2
79	4	4	4	4	4	4
80	3	3	3	3	3	3
81	2	3	2	2	3	2
82	4	3	4	4	4	4
83	4	4	4	4	4	4
84	2	2	2	2	2	2
85	3	2	2	3	3	2
86	4	4	4	4	4	4
87	2	2	3	2	3	2
88	3	2	3	3	3	2

ID	RS1	RS2	RS3	RS4	RS5	RS6
89	4	4	4	4	4	4
90	3	3	3	3	3	3
91	2	3	2	2	3	2
92	4	3	4	4	4	4
93	4	4	4	4	4	4
94	2	2	2	2	2	2
95	3	2	2	3	3	2
96	4	4	4	4	4	4
97	2	2	3	2	3	2
98	3	2	3	3	3	2
99	4	4	4	4	4	4
100	3	3	3	3	3	3
101	2	3	2	2	3	2
102	4	3	4	4	4	4
103	4	4	4	4	4	4
104	2	2	2	2	2	2
105	3	2	2	3	3	2
106	4	4	4	4	4	4
107	2	2	3	2	3	2
108	3	2	3	3	3	2
109	4	4	4	4	4	4
110	3	3	3	3	3	3
111	2	3	2	2	3	2
112	4	3	4	4	4	4
113	4	4	4	4	4	4
114	2	2	2	2	2	2
115	3	2	2	3	3	2
116	4	4	4	4	4	4
117	2	2	3	2	3	2
118	3	2	3	3	3	2
119	4	4	4	4	4	4
120	3	3	3	3	3	3
121	2	3	2	2	3	2
122	4	3	4	4	4	4
123	4	4	4	4	4	4
124	2	2	2	2	2	2
125	3	2	2	3	3	2
126	4	4	4	4	4	4
127	2	2	3	2	3	2
128	3	2	3	3	3	2
129	4	4	4	4	4	4
130	3	3	3	3	3	3
131	2	3	2	2	3	2
132	4	3	4	4	4	4
133	4	4	4	4	4	4

ID	RS1	RS2	RS3	RS4	RS5	RS6
134	2	2	2	2	2	2
135	3	2	2	3	3	2
136	4	4	4	4	4	4
137	2	2	3	2	3	2
138	3	2	3	3	3	2
139	4	4	4	4	4	4
140	3	3	3	3	3	3
141	2	3	2	2	3	2
142	4	3	4	4	4	4
143	4	4	4	4	4	4
144	2	2	2	2	2	2
145	3	2	2	3	3	2
146	4	4	4	4	4	4
147	2	2	3	2	3	2
148	3	2	3	3	3	2
149	4	4	4	4	4	4
150	3	3	3	3	3	3
151	2	3	2	2	3	2
152	4	3	4	4	4	4
153	4	4	4	4	4	4
154	2	2	2	2	2	2
155	3	2	2	3	3	2
156	4	4	4	4	4	4
157	2	2	3	2	3	2
158	3	2	3	3	3	2
159	4	4	4	4	4	4
160	3	3	3	3	3	3
161	3	3	4	3	4	3
162	3	3	3	3	4	3
163	4	3	4	4	4	4
164	3	3	3	3	4	3
165	4	4	4	4	4	4
166	3	3	3	3	3	3
167	2	3	2	2	3	2
168	4	4	4	4	4	4
169	3	2	3	3	3	2
170	3	3	3	3	3	3
171	2	2	3	2	3	2
172	4	4	4	4	4	4
173	2	3	2	2	3	2
174	4	3	4	4	4	3
175	3	4	3	4	4	3
176	3	2	3	3	3	2
177	2	2	2	2	2	2
178	4	4	4	4	4	4

ID	RS1	RS2	RS3	RS4	RS5	RS6
179	2	2	3	2	3	2
180	3	3	3	3	3	3
181	4	4	3	4	4	4
182	2	2	2	2	2	2
183	3	3	4	3	4	3
184	4	4	4	4	4	4
185	2	3	2	2	3	2
186	4	3	4	4	4	3
187	3	4	3	4	4	3
188	3	2	3	3	3	2
189	2	2	2	2	2	2
190	4	4	4	4	4	4
191	2	2	3	2	3	2
192	3	3	3	3	3	3
193	4	4	3	4	4	4
194	2	2	2	2	2	2
195	3	3	4	3	4	3
196	4	4	4	4	4	4
197	2	3	2	2	3	2
198	4	3	4	4	4	3
199	3	4	3	4	4	3
200	3	2	3	3	3	2
201	2	2	2	2	2	2
202	4	4	4	4	4	4
203	2	2	3	2	3	2
204	3	3	3	3	3	3
205	4	4	3	4	4	4
206	2	2	2	2	2	2
207	3	3	4	3	4	3
208	4	4	4	4	4	4
209	2	3	2	2	3	2
210	4	3	4	4	4	3
211	3	4	3	4	4	3
212	3	2	3	3	3	2
213	2	2	2	2	2	2
214	4	4	4	4	4	4
215	2	2	3	2	3	2
216	3	3	3	3	3	3
217	4	4	3	4	4	4
218	2	2	2	2	2	2
219	3	3	4	3	4	3
220	4	4	4	4	4	4
221	2	3	2	2	3	2
222	4	3	4	4	4	3
223	3	4	3	4	4	3

ID	RS1	RS2	RS3	RS4	RS5	RS6
224	3	2	3	3	3	2
225	2	2	2	2	2	2
226	4	4	4	4	4	4
227	2	2	3	2	3	2
228	3	3	3	3	3	3
229	4	4	3	4	4	4
230	2	2	2	2	2	2
231	3	3	4	3	4	3
232	4	4	4	4	4	4
233	2	3	2	2	3	2
234	4	3	4	4	4	3
235	3	4	3	4	4	3
236	3	2	3	3	3	2
237	2	2	2	2	2	2
238	4	4	4	4	4	4
239	2	2	3	2	3	2
240	3	3	3	3	3	3
241	4	4	3	4	4	4
242	2	2	2	2	2	2
243	3	3	4	3	4	3
244	4	4	4	4	4	4
245	2	3	2	2	3	2
246	4	3	4	4	4	3
247	3	4	3	4	4	3
248	3	2	3	3	3	2
249	2	2	2	2	2	2
250	4	4	4	4	4	4
251	2	2	3	2	3	2
252	3	3	3	3	3	3
253	4	4	3	4	4	4
254	2	2	2	2	2	2
255	3	3	4	3	4	3
256	4	4	4	4	4	4
257	2	3	2	2	3	2
258	4	3	4	4	4	3
259	3	4	3	4	4	3
260	3	2	3	3	3	2
261	2	2	2	2	2	2
262	4	4	4	4	4	4
263	2	2	3	2	3	2
264	3	3	3	3	3	3
265	4	4	3	4	4	4
266	2	2	2	2	2	2
267	3	3	4	3	4	3
268	4	4	4	4	4	4

ID	RS1	RS2	RS3	RS4	RS5	RS6
269	2	3	2	2	3	2
270	4	3	4	4	4	3
271	3	4	3	4	4	3
272	3	2	3	3	3	2
273	2	2	2	2	2	2
274	4	4	4	4	4	4
275	2	2	3	2	3	2
276	3	3	3	3	3	3
277	4	4	3	4	4	4
278	2	2	2	2	2	2
279	3	3	4	3	4	3
280	4	4	4	4	4	4
281	2	3	2	2	3	2
282	4	3	4	4	4	3
283	3	4	3	4	4	3
284	3	2	3	3	3	2
285	2	2	2	2	2	2
286	4	4	4	4	4	4
287	2	2	3	2	3	2
288	3	3	3	3	3	3
289	4	4	3	4	4	4
290	2	2	2	2	2	2
291	3	3	4	3	4	3
292	4	4	4	4	4	4
293	2	3	2	2	3	2
294	4	3	4	4	4	3
295	3	4	3	4	4	3
296	3	2	3	3	3	2
297	2	2	2	2	2	2
298	4	4	4	4	4	4
299	2	2	3	2	3	2
300	3	3	3	3	3	3
301	4	4	3	4	4	4
302	2	2	2	2	2	2
303	3	3	4	3	4	3
304	4	4	4	4	4	4
305	2	3	2	2	3	2
306	4	3	4	4	4	3
307	3	4	3	4	4	3
308	3	2	3	3	3	2
309	2	2	2	2	2	2
310	4	4	4	4	4	4
311	2	2	3	2	3	2
312	3	3	3	3	3	3
313	4	4	3	4	4	4

ID	RS1	RS2	RS3	RS4	RS5	RS6
314	2	2	2	2	2	2
315	3	3	4	3	4	3
316	4	4	4	4	4	4
317	2	3	2	2	3	2
318	4	3	4	4	4	3
319	3	4	3	4	4	3
320	3	2	3	3	3	2
321	2	2	2	2	2	2
322	4	4	4	4	4	4
323	2	2	3	2	3	2
324	3	3	3	3	3	3
325	4	4	3	4	4	4
326	2	2	2	2	2	2
327	3	3	4	3	4	3
328	4	4	4	4	4	4
329	2	3	2	2	3	2
330	4	3	4	4	4	3
331	3	4	3	4	4	3
332	3	2	3	3	3	2
333	2	2	2	2	2	2
334	4	4	4	4	4	4
335	2	2	3	2	3	2
336	3	3	3	3	3	3
337	4	4	3	4	4	4
338	2	2	2	2	2	2
339	3	3	4	3	4	3
340	4	4	4	4	4	4
341	2	3	2	2	3	2
342	4	3	4	4	4	3
343	3	4	3	4	4	3
344	3	2	3	3	3	2
345	2	2	2	2	2	2
346	4	4	4	4	4	4
347	2	2	3	2	3	2
348	3	3	3	3	3	3
349	4	4	3	4	4	4
350	2	2	2	2	2	2
351	3	3	4	3	4	3
352	4	4	4	4	4	4
353	2	3	2	2	3	2
354	4	3	4	4	4	3
355	3	4	3	4	4	3
356	3	2	3	3	3	2
357	2	2	2	2	2	2
358	4	4	4	4	4	4

ID	RS1	RS2	RS3	RS4	RS5	RS6
359	2	2	3	2	3	2
360	3	3	3	3	3	3
361	4	4	3	4	4	4
362	2	2	2	2	2	2
363	3	3	4	3	4	3
364	4	4	4	4	4	4
365	2	3	2	2	3	2
366	4	3	4	4	4	3
367	3	4	3	4	4	3
368	3	2	3	3	3	2
369	2	2	2	2	2	2
370	4	4	4	4	4	4
371	2	2	3	2	3	2
372	3	3	3	3	3	3
373	4	4	3	4	4	4
374	2	2	2	2	2	2
375	3	3	4	3	4	3
376	4	4	4	4	4	4
377	2	3	2	2	3	2
378	4	3	4	4	4	3
379	3	4	3	4	4	3
380	3	2	3	3	3	2
381	2	2	2	2	2	2
382	4	4	4	4	4	4
383	2	2	3	2	3	2
384	3	3	3	3	3	3
385	4	4	3	4	4	4
386	2	2	2	2	2	2
387	3	3	4	3	4	3
388	4	4	4	4	4	4
389	2	3	2	2	3	2
390	4	3	4	4	4	3
391	3	4	3	4	4	3
392	3	2	3	3	3	2
393	2	2	2	2	2	2
394	4	4	4	4	4	4
395	2	2	3	2	3	2
396	3	3	3	3	3	3
397	4	4	3	4	4	4
398	2	2	2	2	2	2
399	3	3	4	3	4	3
400	4	4	4	4	4	4
401	2	3	2	2	3	2
402	4	3	4	4	4	3
403	3	4	3	4	4	3

ID	RS1	RS2	RS3	RS4	RS5	RS6
404	3	2	3	3	3	2
405	2	2	2	2	2	2
406	4	4	4	4	4	4
407	2	2	3	2	3	2
408	3	3	3	3	3	3
409	4	4	3	4	4	4
410	2	2	2	2	2	2
411	3	3	4	3	4	3
412	4	4	4	4	4	4
413	2	3	2	2	3	2
414	4	3	4	4	4	3
415	3	4	3	4	4	3
416	3	2	3	3	3	2
417	2	2	2	2	2	2
418	4	4	4	4	4	4
419	2	2	3	2	3	2
420	3	3	3	3	3	3
421	4	4	3	4	4	4
422	2	2	2	2	2	2
423	3	3	4	3	4	3
424	4	4	4	4	4	4
425	2	3	2	2	3	2
426	4	3	4	4	4	3
427	3	4	3	4	4	3
428	3	2	3	3	3	2
429	2	2	2	2	2	2
430	4	4	4	4	4	4
431	2	2	3	2	3	2
432	3	3	3	3	3	3
433	4	4	3	4	4	4
434	2	2	2	2	2	2
435	3	3	4	3	4	3
436	4	4	4	4	4	4
437	2	3	2	2	3	2
438	4	3	4	4	4	3
439	3	4	3	4	4	3
440	3	2	3	3	3	2
441	2	2	2	2	2	2
442	4	4	4	4	4	4
443	2	2	3	2	3	2
444	3	3	3	3	3	3
445	4	4	3	4	4	4
446	2	2	2	2	2	2
447	3	3	4	3	4	3
448	4	4	4	4	4	4

ID	RS1	RS2	RS3	RS4	RS5	RS6
449	3	3	3	3	4	3
450	3	2	3	3	3	2
451	3	3	3	4	3	4
452	4	4	4	4	4	4
453	3	2	2	3	3	2
454	3	2	3	3	3	3
455	4	4	4	3	4	4
456	3	3	3	3	4	3
457	2	3	2	2	3	2
458	3	3	3	3	4	3
459	3	3	2	3	3	2
460	4	4	4	4	4	4
461	3	2	2	2	3	2
462	2	2	2	2	2	2
463	2	2	2	2	3	2
464	3	2	3	3	3	2
465	2	2	2	3	2	2
466	4	3	4	4	4	4
467	2	2	2	2	2	2
468	1	2	1	2	2	2
469	3	4	3	3	3	3
470	2	2	2	2	3	2
471	4	4	4	4	4	4
472	2	3	2	2	3	2
473	4	3	4	4	4	3
474	3	4	3	4	4	3
475	3	2	3	3	3	2
476	2	2	2	2	2	2
477	4	4	4	4	4	4
478	2	2	3	2	3	2
479	3	3	3	3	3	3
480	4	4	3	4	4	4
481	2	2	2	2	2	2
482	3	3	4	3	4	3
483	4	4	4	4	4	4
484	2	3	2	2	3	2
485	4	3	4	4	4	3
486	3	4	3	4	4	3
487	3	2	3	3	3	2
488	2	2	2	2	2	2
489	4	4	4	4	4	4
490	2	2	3	2	3	2
491	3	3	3	3	3	3
492	4	4	3	4	4	4
493	2	2	2	2	2	2

ID	RS1	RS2	RS3	RS4	RS5	RS6
494	3	3	4	3	4	3
495	4	4	4	4	4	4
496	2	3	2	2	3	2
497	4	3	4	4	4	3
498	3	4	3	4	4	3
499	3	2	3	3	3	2
500	2	2	2	2	2	2
501	4	4	4	4	4	4
502	2	2	3	2	3	2
503	3	3	3	3	3	3
504	4	4	3	4	4	4
505	2	2	2	2	2	2
506	3	3	4	3	4	3
507	4	4	4	4	4	4
508	2	3	2	2	3	2
509	4	3	4	4	4	3
510	3	4	3	4	4	3
511	3	2	3	3	3	2
512	2	2	2	2	2	2
513	4	4	4	4	4	4
514	2	2	3	2	3	2
515	3	3	3	3	3	3
516	4	4	3	4	4	4
517	2	2	2	2	2	2
518	3	3	4	3	4	3
519	4	4	4	4	4	4
520	2	3	2	2	3	2
521	4	3	4	4	4	3
522	3	4	3	4	4	3
523	3	2	3	3	3	2
524	2	2	2	2	2	2
525	4	4	4	4	4	4
526	2	2	3	2	3	2
527	3	3	3	3	3	3
528	4	4	3	4	4	4
529	2	2	2	2	2	2
530	3	3	4	3	4	3
531	4	4	4	4	4	4
532	2	3	2	2	3	2
533	4	3	4	4	4	3
534	3	4	3	4	4	3
535	3	2	3	3	3	2
536	2	2	2	2	2	2
537	4	4	4	4	4	4
538	2	2	3	2	3	2

ID	RS1	RS2	RS3	RS4	RS5	RS6
539	3	3	3	3	3	3
540	4	4	3	4	4	4
541	2	2	2	2	2	2
542	3	3	4	3	4	3
543	4	4	4	4	4	4
544	2	3	2	2	3	2
545	4	3	4	4	4	3
546	3	4	3	4	4	3
547	3	2	3	3	3	2
548	2	2	2	2	2	2
549	4	4	4	4	4	4
550	2	2	3	2	3	2
551	3	3	3	3	3	3
552	4	4	3	4	4	4
553	2	2	2	2	2	2
554	3	3	4	3	4	3
555	4	4	4	4	4	4
556	2	3	2	2	3	2
557	4	3	4	4	4	3
558	3	4	3	4	4	3
559	3	2	3	3	3	2
560	2	2	2	2	2	2
561	4	4	4	4	4	4
562	2	2	3	2	3	2
563	3	3	3	3	3	3
564	4	4	3	4	4	4
565	2	2	2	2	2	2
566	3	3	4	3	4	3
567	4	4	4	4	4	4
568	2	3	2	2	3	2
569	4	3	4	4	4	3
570	3	4	3	4	4	3
571	3	2	3	3	3	2
572	2	2	2	2	2	2
573	4	4	4	4	4	4
574	2	2	3	2	3	2
575	3	3	3	3	3	3
576	4	4	3	4	4	4
577	2	2	2	2	2	2
578	3	3	4	3	4	3
579	4	4	4	4	4	4
580	2	3	2	2	3	2
581	4	3	4	4	4	3
582	3	4	3	4	4	3
583	3	2	3	3	3	2

ID	RS1	RS2	RS3	RS4	RS5	RS6
584	2	2	2	2	2	2
585	4	4	4	4	4	4
586	2	2	3	2	3	2
587	3	3	3	3	3	3
588	4	4	3	4	4	4
589	2	2	2	2	2	2
590	3	3	4	3	4	3
591	4	4	4	4	4	4
592	2	3	2	2	3	2
593	4	3	4	4	4	3
594	3	4	3	4	4	3
595	3	2	3	3	3	2
596	2	2	2	2	2	2
597	4	4	4	4	4	4
598	2	2	3	2	3	2
599	3	3	3	3	3	3
600	4	4	3	4	4	4
601	2	2	2	2	2	2
602	3	3	4	3	4	3
603	4	4	4	4	4	4
604	2	3	2	2	3	2
605	4	3	4	4	4	3
606	3	4	3	4	4	3
607	3	2	3	3	3	2
608	2	2	2	2	2	2
609	4	4	4	4	4	4
610	2	2	3	2	3	2
611	3	3	3	3	3	3
612	4	4	3	4	4	4
613	2	2	2	2	2	2
614	3	3	4	3	4	3
615	4	4	4	4	4	4
616	2	3	2	2	3	2
617	4	3	4	4	4	3
618	3	4	3	4	4	3
619	3	2	3	3	3	2
620	2	2	2	2	2	2
621	4	4	4	4	4	4
622	2	2	3	2	3	2
623	3	3	3	3	3	3
624	4	4	3	4	4	4
625	2	2	2	2	2	2
626	3	3	4	3	4	3
627	4	4	4	4	4	4
628	2	3	2	2	3	2

ID	RS1	RS2	RS3	RS4	RS5	RS6
629	4	3	4	4	4	3
630	3	4	3	4	4	3
631	3	2	3	3	3	2
632	2	2	2	2	2	2
633	4	4	4	4	4	4
634	2	2	3	2	3	2
635	3	3	3	3	3	3
636	4	4	3	4	4	4
637	2	2	2	2	2	2
638	3	3	4	3	4	3
639	4	4	4	4	4	4
640	2	3	2	2	3	2

Table 7: Raw data, Panel C: Mood Dysregulation items

ID	MD1	MD2	MD3	MD4	MD5	MD6	MD7
1	1	2	1	1	2	1	1
2	2	2	2	2	2	2	2
3	3	3	3	2	3	2	3
4	2	2	2	2	2	2	2
5	3	3	3	3	3	3	3
6	1	1	2	1	1	1	2
7	2	2	2	2	2	2	2
8	2	1	2	1	2	2	1
9	3	2	3	2	3	3	2
10	2	3	2	2	2	2	2
11	2	2	2	1	2	1	2
12	3	2	3	2	3	3	2
13	2	2	2	2	2	2	2
14	1	1	1	1	2	1	1
15	2	2	2	2	2	2	2
16	3	3	3	3	3	3	3
17	1	2	1	1	2	1	1
18	2	2	2	2	2	2	2
19	2	2	2	2	2	2	2
20	1	1	1	2	1	1	1
21	3	3	3	2	3	3	3
22	2	2	2	2	2	2	3
23	2	2	2	1	2	1	2
24	2	1	2	1	2	2	2
25	3	2	3	2	3	3	2
26	1	1	1	1	1	1	1
27	2	2	2	2	2	2	2
28	3	3	3	3	3	3	3
29	2	2	2	2	2	2	3
30	2	2	1	2	2	2	2
31	3	3	3	2	3	3	3
32	2	2	2	2	2	2	2
33	2	2	2	2	2	2	2
34	2	2	2	2	2	2	2
35	3	3	3	3	3	3	3
36	2	3	2	2	2	2	2
37	1	2	1	1	2	1	1
38	1	1	1	1	1	1	1
39	3	2	3	2	3	3	2
40	2	2	2	2	2	2	3
41	2	2	2	1	2	1	2
42	3	3	2	3	3	3	3
43	3	3	3	3	3	3	3

ID	MD1	MD2	MD3	MD4	MD5	MD6	MD7
44	1	1	1	1	2	1	1
45	2	2	2	2	2	2	2
46	3	3	3	3	3	3	3
47	1	2	1	1	2	1	1
48	2	2	2	2	2	2	2
49	3	3	3	3	3	3	3
50	2	2	2	2	2	2	3
51	2	2	2	1	2	1	2
52	3	3	2	3	3	3	3
53	3	3	3	3	3	3	3
54	1	1	1	1	2	1	1
55	2	2	2	2	2	2	2
56	3	3	3	3	3	3	3
57	1	2	1	1	2	1	1
58	2	2	2	2	2	2	2
59	3	3	3	3	3	3	3
60	2	2	2	2	2	2	3
61	2	2	2	1	2	1	2
62	3	3	2	3	3	3	3
63	3	3	3	3	3	3	3
64	1	1	1	1	2	1	1
65	2	2	2	2	2	2	2
66	3	3	3	3	3	3	3
67	1	2	1	1	2	1	1
68	2	2	2	2	2	2	2
69	3	3	3	3	3	3	3
70	2	2	2	2	2	2	3
71	2	2	2	1	2	1	2
72	3	3	2	3	3	3	3
73	3	3	3	3	3	3	3
74	1	1	1	1	2	1	1
75	2	2	2	2	2	2	2
76	3	3	3	3	3	3	3
77	1	2	1	1	2	1	1
78	2	2	2	2	2	2	2
79	3	3	3	3	3	3	3
80	2	2	2	2	2	2	3
81	2	2	2	1	2	1	2
82	3	3	2	3	3	3	3
83	3	3	3	3	3	3	3
84	1	1	1	1	2	1	1
85	2	2	2	2	2	2	2
86	3	3	3	3	3	3	3
87	1	2	1	1	2	1	1
88	2	2	2	2	2	2	2

ID	MD1	MD2	MD3	MD4	MD5	MD6	MD7
89	3	3	3	3	3	3	3
90	2	2	2	2	2	2	3
91	2	2	2	1	2	1	2
92	3	3	2	3	3	3	3
93	3	3	3	3	3	3	3
94	1	1	1	1	2	1	1
95	2	2	2	2	2	2	2
96	3	3	3	3	3	3	3
97	1	2	1	1	2	1	1
98	2	2	2	2	2	2	2
99	3	3	3	3	3	3	3
100	2	2	2	2	2	2	3
101	2	2	2	1	2	1	2
102	3	3	2	3	3	3	3
103	3	3	3	3	3	3	3
104	1	1	1	1	2	1	1
105	2	2	2	2	2	2	2
106	3	3	3	3	3	3	3
107	1	2	1	1	2	1	1
108	2	2	2	2	2	2	2
109	3	3	3	3	3	3	3
110	2	2	2	2	2	2	3
111	2	2	2	1	2	1	2
112	3	3	2	3	3	3	3
113	3	3	3	3	3	3	3
114	1	1	1	1	2	1	1
115	2	2	2	2	2	2	2
116	3	3	3	3	3	3	3
117	1	2	1	1	2	1	1
118	2	2	2	2	2	2	2
119	3	3	3	3	3	3	3
120	2	2	2	2	2	2	3
121	2	2	2	1	2	1	2
122	3	3	2	3	3	3	3
123	3	3	3	3	3	3	3
124	1	1	1	1	2	1	1
125	2	2	2	2	2	2	2
126	3	3	3	3	3	3	3
127	1	2	1	1	2	1	1
128	2	2	2	2	2	2	2
129	3	3	3	3	3	3	3
130	2	2	2	2	2	2	3
131	2	2	2	1	2	1	2
132	3	3	2	3	3	3	3
133	3	3	3	3	3	3	3

ID	MD1	MD2	MD3	MD4	MD5	MD6	MD7
134	1	1	1	1	2	1	1
135	2	2	2	2	2	2	2
136	3	3	3	3	3	3	3
137	1	2	1	1	2	1	1
138	2	2	2	2	2	2	2
139	3	3	3	3	3	3	3
140	2	2	2	2	2	2	3
141	2	2	2	1	2	1	2
142	3	3	2	3	3	3	3
143	3	3	3	3	3	3	3
144	1	1	1	1	2	1	1
145	2	2	2	2	2	2	2
146	3	3	3	3	3	3	3
147	1	2	1	1	2	1	1
148	2	2	2	2	2	2	2
149	3	3	3	3	3	3	3
150	2	2	2	2	2	2	3
151	2	2	2	1	2	1	2
152	3	3	2	3	3	3	3
153	3	3	3	3	3	3	3
154	1	1	1	1	2	1	1
155	2	2	2	2	2	2	2
156	3	3	3	3	3	3	3
157	1	2	1	1	2	1	1
158	2	2	2	2	2	2	2
159	3	3	3	3	3	3	3
160	2	2	2	2	2	2	3
161	2	2	2	2	2	2	2
162	2	3	2	2	3	2	3
163	3	3	3	3	3	3	3
164	2	2	2	2	2	2	2
165	3	3	3	3	3	3	3
166	2	2	2	2	2	2	2
167	2	2	1	2	2	2	2
168	3	3	3	2	3	3	3
169	2	2	2	2	2	2	2
170	2	3	2	2	2	2	2
171	1	2	1	1	2	1	1
172	3	3	3	3	3	3	3
173	2	2	2	1	2	1	2
174	3	2	3	2	3	3	2
175	2	2	2	2	2	2	2
176	2	2	2	2	2	2	2
177	1	1	1	1	2	1	1
178	3	3	3	2	3	3	3

ID	MD1	MD2	MD3	MD4	MD5	MD6	MD7
179	1	2	1	1	2	1	1
180	2	3	2	2	2	2	2
181	3	2	3	2	3	3	2
182	1	1	1	2	1	1	1
183	2	2	2	2	2	2	2
184	3	3	3	3	3	3	3
185	2	2	2	1	2	1	2
186	3	2	3	2	3	3	2
187	2	2	2	2	2	2	2
188	2	2	2	2	2	2	2
189	1	1	1	1	2	1	1
190	3	3	3	2	3	3	3
191	1	2	1	1	2	1	1
192	2	3	2	2	2	2	2
193	3	2	3	2	3	3	2
194	1	1	1	2	1	1	1
195	2	2	2	2	2	2	2
196	3	3	3	3	3	3	3
197	2	2	2	1	2	1	2
198	3	2	3	2	3	3	2
199	2	2	2	2	2	2	2
200	2	2	2	2	2	2	2
201	1	1	1	1	2	1	1
202	3	3	3	2	3	3	3
203	1	2	1	1	2	1	1
204	2	3	2	2	2	2	2
205	3	2	3	2	3	3	2
206	1	1	1	2	1	1	1
207	2	2	2	2	2	2	2
208	3	3	3	3	3	3	3
209	2	2	2	1	2	1	2
210	3	2	3	2	3	3	2
211	2	2	2	2	2	2	2
212	2	2	2	2	2	2	2
213	1	1	1	1	2	1	1
214	3	3	3	2	3	3	3
215	1	2	1	1	2	1	1
216	2	3	2	2	2	2	2
217	3	2	3	2	3	3	2
218	1	1	1	2	1	1	1
219	2	2	2	2	2	2	2
220	3	3	3	3	3	3	3
221	2	2	2	1	2	1	2
222	3	2	3	2	3	3	2
223	2	2	2	2	2	2	2

ID	MD1	MD2	MD3	MD4	MD5	MD6	MD7
224	2	2	2	2	2	2	2
225	1	1	1	1	2	1	1
226	3	3	3	2	3	3	3
227	1	2	1	1	2	1	1
228	2	3	2	2	2	2	2
229	3	2	3	2	3	3	2
230	1	1	1	2	1	1	1
231	2	2	2	2	2	2	2
232	3	3	3	3	3	3	3
233	2	2	2	1	2	1	2
234	3	2	3	2	3	3	2
235	2	2	2	2	2	2	2
236	2	2	2	2	2	2	2
237	1	1	1	1	2	1	1
238	3	3	3	2	3	3	3
239	1	2	1	1	2	1	1
240	2	3	2	2	2	2	2
241	3	2	3	2	3	3	2
242	1	1	1	2	1	1	1
243	2	2	2	2	2	2	2
244	3	3	3	3	3	3	3
245	2	2	2	1	2	1	2
246	3	2	3	2	3	3	2
247	2	2	2	2	2	2	2
248	2	2	2	2	2	2	2
249	1	1	1	1	2	1	1
250	3	3	3	2	3	3	3
251	1	2	1	1	2	1	1
252	2	3	2	2	2	2	2
253	3	2	3	2	3	3	2
254	1	1	1	2	1	1	1
255	2	2	2	2	2	2	2
256	3	3	3	3	3	3	3
257	2	2	2	1	2	1	2
258	3	2	3	2	3	3	2
259	2	2	2	2	2	2	2
260	2	2	2	2	2	2	2
261	1	1	1	1	2	1	1
262	3	3	3	2	3	3	3
263	1	2	1	1	2	1	1
264	2	3	2	2	2	2	2
265	3	2	3	2	3	3	2
266	1	1	1	2	1	1	1
267	2	2	2	2	2	2	2
268	3	3	3	3	3	3	3

ID	MD1	MD2	MD3	MD4	MD5	MD6	MD7
269	2	2	2	1	2	1	2
270	3	2	3	2	3	3	2
271	2	2	2	2	2	2	2
272	2	2	2	2	2	2	2
273	1	1	1	1	2	1	1
274	3	3	3	2	3	3	3
275	1	2	1	1	2	1	1
276	2	3	2	2	2	2	2
277	3	2	3	2	3	3	2
278	1	1	1	2	1	1	1
279	2	2	2	2	2	2	2
280	3	3	3	3	3	3	3
281	2	2	2	1	2	1	2
282	3	2	3	2	3	3	2
283	2	2	2	2	2	2	2
284	2	2	2	2	2	2	2
285	1	1	1	1	2	1	1
286	3	3	3	2	3	3	3
287	1	2	1	1	2	1	1
288	2	3	2	2	2	2	2
289	3	2	3	2	3	3	2
290	1	1	1	2	1	1	1
291	2	2	2	2	2	2	2
292	3	3	3	3	3	3	3
293	2	2	2	1	2	1	2
294	3	2	3	2	3	3	2
295	2	2	2	2	2	2	2
296	2	2	2	2	2	2	2
297	1	1	1	1	2	1	1
298	3	3	3	2	3	3	3
299	1	2	1	1	2	1	1
300	2	3	2	2	2	2	2
301	3	2	3	2	3	3	2
302	1	1	1	2	1	1	1
303	2	2	2	2	2	2	2
304	3	3	3	3	3	3	3
305	2	2	2	1	2	1	2
306	3	2	3	2	3	3	2
307	2	2	2	2	2	2	2
308	2	2	2	2	2	2	2
309	1	1	1	1	2	1	1
310	3	3	3	2	3	3	3
311	1	2	1	1	2	1	1
312	2	3	2	2	2	2	2
313	3	2	3	2	3	3	2

ID	MD1	MD2	MD3	MD4	MD5	MD6	MD7
314	1	1	1	2	1	1	1
315	2	2	2	2	2	2	2
316	3	3	3	3	3	3	3
317	2	2	2	1	2	1	2
318	3	2	3	2	3	3	2
319	2	2	2	2	2	2	2
320	2	2	2	2	2	2	2
321	1	1	1	1	2	1	1
322	3	3	3	2	3	3	3
323	1	2	1	1	2	1	1
324	2	3	2	2	2	2	2
325	3	2	3	2	3	3	2
326	1	1	1	2	1	1	1
327	2	2	2	2	2	2	2
328	3	3	3	3	3	3	3
329	2	2	2	1	2	1	2
330	3	2	3	2	3	3	2
331	2	2	2	2	2	2	2
332	2	2	2	2	2	2	2
333	1	1	1	1	2	1	1
334	3	3	3	2	3	3	3
335	1	2	1	1	2	1	1
336	2	3	2	2	2	2	2
337	3	2	3	2	3	3	2
338	1	1	1	2	1	1	1
339	2	2	2	2	2	2	2
340	3	3	3	3	3	3	3
341	2	2	2	1	2	1	2
342	3	2	3	2	3	3	2
343	2	2	2	2	2	2	2
344	2	2	2	2	2	2	2
345	1	1	1	1	2	1	1
346	3	3	3	2	3	3	3
347	1	2	1	1	2	1	1
348	2	3	2	2	2	2	2
349	3	2	3	2	3	3	2
350	1	1	1	2	1	1	1
351	2	2	2	2	2	2	2
352	3	3	3	3	3	3	3
353	2	2	2	1	2	1	2
354	3	2	3	2	3	3	2
355	2	2	2	2	2	2	2
356	2	2	2	2	2	2	2
357	1	1	1	1	2	1	1
358	3	3	3	2	3	3	3

ID	MD1	MD2	MD3	MD4	MD5	MD6	MD7
359	1	2	1	1	2	1	1
360	2	3	2	2	2	2	2
361	3	2	3	2	3	3	2
362	1	1	1	2	1	1	1
363	2	2	2	2	2	2	2
364	3	3	3	3	3	3	3
365	2	2	2	1	2	1	2
366	3	2	3	2	3	3	2
367	2	2	2	2	2	2	2
368	2	2	2	2	2	2	2
369	1	1	1	1	2	1	1
370	3	3	3	2	3	3	3
371	1	2	1	1	2	1	1
372	2	3	2	2	2	2	2
373	3	2	3	2	3	3	2
374	1	1	1	2	1	1	1
375	2	2	2	2	2	2	2
376	3	3	3	3	3	3	3
377	2	2	2	1	2	1	2
378	3	2	3	2	3	3	2
379	2	2	2	2	2	2	2
380	2	2	2	2	2	2	2
381	1	1	1	1	2	1	1
382	3	3	3	2	3	3	3
383	1	2	1	1	2	1	1
384	2	3	2	2	2	2	2
385	3	2	3	2	3	3	2
386	1	1	1	2	1	1	1
387	2	2	2	2	2	2	2
388	3	3	3	3	3	3	3
389	2	2	2	1	2	1	2
390	3	2	3	2	3	3	2
391	2	2	2	2	2	2	2
392	2	2	2	2	2	2	2
393	1	1	1	1	2	1	1
394	3	3	3	2	3	3	3
395	1	2	1	1	2	1	1
396	2	3	2	2	2	2	2
397	3	2	3	2	3	3	2
398	1	1	1	2	1	1	1
399	2	2	2	2	2	2	2
400	3	3	3	3	3	3	3
401	2	2	2	1	2	1	2
402	3	2	3	2	3	3	2
403	2	2	2	2	2	2	2

ID	MD1	MD2	MD3	MD4	MD5	MD6	MD7
404	2	2	2	2	2	2	2
405	1	1	1	1	2	1	1
406	3	3	3	2	3	3	3
407	1	2	1	1	2	1	1
408	2	3	2	2	2	2	2
409	3	2	3	2	3	3	2
410	1	1	1	2	1	1	1
411	2	2	2	2	2	2	2
412	3	3	3	3	3	3	3
413	2	2	2	1	2	1	2
414	3	2	3	2	3	3	2
415	2	2	2	2	2	2	2
416	2	2	2	2	2	2	2
417	1	1	1	1	2	1	1
418	3	3	3	2	3	3	3
419	1	2	1	1	2	1	1
420	2	3	2	2	2	2	2
421	3	2	3	2	3	3	2
422	1	1	1	2	1	1	1
423	2	2	2	2	2	2	2
424	3	3	3	3	3	3	3
425	2	2	2	1	2	1	2
426	3	2	3	2	3	3	2
427	2	2	2	2	2	2	2
428	2	2	2	2	2	2	2
429	1	1	1	1	2	1	1
430	3	3	3	2	3	3	3
431	1	2	1	1	2	1	1
432	2	3	2	2	2	2	2
433	3	2	3	2	3	3	2
434	1	1	1	2	1	1	1
435	2	2	2	2	2	2	2
436	3	3	3	3	3	3	3
437	2	2	2	1	2	1	2
438	3	2	3	2	3	3	2
439	2	2	2	2	2	2	2
440	2	2	2	2	2	2	2
441	1	1	1	1	2	1	1
442	3	3	3	2	3	3	3
443	1	2	1	1	2	1	1
444	2	3	2	2	2	2	2
445	3	2	3	2	3	3	2
446	1	1	1	2	1	1	1
447	2	2	2	2	2	2	2
448	3	3	3	3	3	3	3

ID	MD1	MD2	MD3	MD4	MD5	MD6	MD7
449	2	2	2	2	2	2	2
450	2	2	2	2	2	2	2
451	2	3	2	2	2	2	3
452	3	3	3	3	3	3	3
453	2	2	2	2	2	2	2
454	2	2	2	2	1	2	2
455	3	2	3	2	3	3	2
456	2	2	2	2	2	2	3
457	2	2	1	2	2	2	2
458	2	2	2	2	2	2	2
459	2	2	2	2	2	2	2
460	3	3	3	2	3	3	3
461	2	2	2	2	2	2	2
462	1	1	1	1	2	1	1
463	1	1	1	1	1	1	1
464	2	2	2	1	2	1	2
465	1	2	2	1	2	1	1
466	3	3	2	3	3	3	3
467	1	1	1	2	1	1	1
468	1	1	1	1	1	1	1
469	2	3	2	2	2	2	3
470	2	2	2	2	2	2	2
471	3	3	3	3	3	3	3
472	2	2	2	1	2	1	2
473	3	2	3	2	3	3	2
474	2	2	2	2	2	2	2
475	2	2	2	2	2	2	2
476	1	1	1	1	2	1	1
477	3	3	3	2	3	3	3
478	1	2	1	1	2	1	1
479	2	3	2	2	2	2	2
480	3	2	3	2	3	3	2
481	1	1	1	2	1	1	1
482	2	2	2	2	2	2	2
483	3	3	3	3	3	3	3
484	2	2	2	1	2	1	2
485	3	2	3	2	3	3	2
486	2	2	2	2	2	2	2
487	2	2	2	2	2	2	2
488	1	1	1	1	2	1	1
489	3	3	3	2	3	3	3
490	1	2	1	1	2	1	1
491	2	3	2	2	2	2	2
492	3	2	3	2	3	3	2
493	1	1	1	2	1	1	1

ID	MD1	MD2	MD3	MD4	MD5	MD6	MD7
494	2	2	2	2	2	2	2
495	3	3	3	3	3	3	3
496	2	2	2	1	2	1	2
497	3	2	3	2	3	3	2
498	2	2	2	2	2	2	2
499	2	2	2	2	2	2	2
500	1	1	1	1	2	1	1
501	3	3	3	2	3	3	3
502	1	2	1	1	2	1	1
503	2	3	2	2	2	2	2
504	3	2	3	2	3	3	2
505	1	1	1	2	1	1	1
506	2	2	2	2	2	2	2
507	3	3	3	3	3	3	3
508	2	2	2	1	2	1	2
509	3	2	3	2	3	3	2
510	2	2	2	2	2	2	2
511	2	2	2	2	2	2	2
512	1	1	1	1	2	1	1
513	3	3	3	2	3	3	3
514	1	2	1	1	2	1	1
515	2	3	2	2	2	2	2
516	3	2	3	2	3	3	2
517	1	1	1	2	1	1	1
518	2	2	2	2	2	2	2
519	3	3	3	3	3	3	3
520	2	2	2	1	2	1	2
521	3	2	3	2	3	3	2
522	2	2	2	2	2	2	2
523	2	2	2	2	2	2	2
524	1	1	1	1	2	1	1
525	3	3	3	2	3	3	3
526	1	2	1	1	2	1	1
527	2	3	2	2	2	2	2
528	3	2	3	2	3	3	2
529	1	1	1	2	1	1	1
530	2	2	2	2	2	2	2
531	3	3	3	3	3	3	3
532	2	2	2	1	2	1	2
533	3	2	3	2	3	3	2
534	2	2	2	2	2	2	2
535	2	2	2	2	2	2	2
536	1	1	1	1	2	1	1
537	3	3	3	2	3	3	3
538	1	2	1	1	2	1	1

ID	MD1	MD2	MD3	MD4	MD5	MD6	MD7
539	2	3	2	2	2	2	2
540	3	2	3	2	3	3	2
541	1	1	1	2	1	1	1
542	2	2	2	2	2	2	2
543	3	3	3	3	3	3	3
544	2	2	2	1	2	1	2
545	3	2	3	2	3	3	2
546	2	2	2	2	2	2	2
547	2	2	2	2	2	2	2
548	1	1	1	1	2	1	1
549	3	3	3	2	3	3	3
550	1	2	1	1	2	1	1
551	2	3	2	2	2	2	2
552	3	2	3	2	3	3	2
553	1	1	1	2	1	1	1
554	2	2	2	2	2	2	2
555	3	3	3	3	3	3	3
556	2	2	2	1	2	1	2
557	3	2	3	2	3	3	2
558	2	2	2	2	2	2	2
559	2	2	2	2	2	2	2
560	1	1	1	1	2	1	1
561	3	3	3	2	3	3	3
562	1	2	1	1	2	1	1
563	2	3	2	2	2	2	2
564	3	2	3	2	3	3	2
565	1	1	1	2	1	1	1
566	2	2	2	2	2	2	2
567	3	3	3	3	3	3	3
568	2	2	2	1	2	1	2
569	3	2	3	2	3	3	2
570	2	2	2	2	2	2	2
571	2	2	2	2	2	2	2
572	1	1	1	1	2	1	1
573	3	3	3	2	3	3	3
574	1	2	1	1	2	1	1
575	2	3	2	2	2	2	2
576	3	2	3	2	3	3	2
577	1	1	1	2	1	1	1
578	2	2	2	2	2	2	2
579	3	3	3	3	3	3	3
580	2	2	2	1	2	1	2
581	3	2	3	2	3	3	2
582	2	2	2	2	2	2	2
583	2	2	2	2	2	2	2

ID	MD1	MD2	MD3	MD4	MD5	MD6	MD7
584	1	1	1	1	2	1	1
585	3	3	3	2	3	3	3
586	1	2	1	1	2	1	1
587	2	3	2	2	2	2	2
588	3	2	3	2	3	3	2
589	1	1	1	2	1	1	1
590	2	2	2	2	2	2	2
591	3	3	3	3	3	3	3
592	2	2	2	1	2	1	2
593	3	2	3	2	3	3	2
594	2	2	2	2	2	2	2
595	2	2	2	2	2	2	2
596	1	1	1	1	2	1	1
597	3	3	3	2	3	3	3
598	1	2	1	1	2	1	1
599	2	3	2	2	2	2	2
600	3	2	3	2	3	3	2
601	1	1	1	2	1	1	1
602	2	2	2	2	2	2	2
603	3	3	3	3	3	3	3
604	2	2	2	1	2	1	2
605	3	2	3	2	3	3	2
606	2	2	2	2	2	2	2
607	2	2	2	2	2	2	2
608	1	1	1	1	2	1	1
609	3	3	3	2	3	3	3
610	1	2	1	1	2	1	1
611	2	3	2	2	2	2	2
612	3	2	3	2	3	3	2
613	1	1	1	2	1	1	1
614	2	2	2	2	2	2	2
615	3	3	3	3	3	3	3
616	2	2	2	1	2	1	2
617	3	2	3	2	3	3	2
618	2	2	2	2	2	2	2
619	2	2	2	2	2	2	2
620	1	1	1	1	2	1	1
621	3	3	3	2	3	3	3
622	1	2	1	1	2	1	1
623	2	3	2	2	2	2	2
624	3	2	3	2	3	3	2
625	1	1	1	2	1	1	1
626	2	2	2	2	2	2	2
627	3	3	3	3	3	3	3
628	2	2	2	1	2	1	2

ID	MD1	MD2	MD3	MD4	MD5	MD6	MD7
629	3	2	3	2	3	3	2
630	2	2	2	2	2	2	2
631	2	2	2	2	2	2	2
632	1	1	1	1	2	1	1
633	3	3	3	2	3	3	3
634	1	2	1	1	2	1	1
635	2	3	2	2	2	2	2
636	3	2	3	2	3	3	2
637	1	1	1	2	1	1	1
638	2	2	2	2	2	2	2
639	3	3	3	3	3	3	3
640	2	2	2	1	2	1	2

C Recalculated Statistical Analysis

C.1 Analysis Specification

All statistics in this appendix were recalculated directly from the supplied 640-row dataset. Analyses were two-sided with $\alpha = .05$. The sequence was: (1) reliability and descriptive statistics; (2) Pearson correlations; (3) gender and school-type comparisons using independent-samples t tests, with Welch-adjusted degrees of freedom where homogeneity of variance was not supported; (4) one-way ANOVA across educational levels with η^2; and (5) three-step hierarchical OLS regression predicting mean mood dysregulation. Variance-inflation factors were examined, followed by an age-omitted sensitivity model because age and educational level are structurally closely related in this 13–19-year-old sample.

No causal or temporal mediation inference is made from the cross-sectional regression. The Step 3 pattern is treated as statistical attenuation after reward sensitivity is entered.

C.2 Sample Profile

Table 8: Demographic profile of the final analytic sample

**Characteristic**	n	%
Gender: Male	322	50.3
Gender: Female	318	49.7
Education: Middle school	160	25.0
Education: High school	288	45.0
Education: University	192	30.0
School type: Public	429	67.0
School type: Private/International	211	33.0
Parent education: Basic/Secondary	241	37.7
Parent education: University	293	45.8
Parent education: Postgraduate	106	16.6
Residence: Urban	423	66.1
Residence: Semi-urban/Rural	217	33.9

C.3 Reliability and Descriptive Statistics

Table 9: Descriptive statistics for the main study variables

**Variable**	**M**	**SD**	**Min**	**Max**	**Skew.**	**Kurt.**	α
Age	16.12	1.97	13.00	19.00	-0.08	-1.19	—
Social media screen time	3.87	1.28	1.10	7.40	0.34	-0.58	—
Gaming screen time	2.58	1.46	0.10	6.60	0.80	-0.03	—
Streaming/video screen time	2.18	0.86	0.50	4.80	0.49	-0.29	—
Browsing/communication screen time	1.33	0.68	0.30	3.60	0.80	0.08	—
Total daily screen time	9.96	3.76	2.50	20.10	0.42	-0.48	.87
Reward sensitivity	3.05	0.74	1.67	4.00	-0.05	-1.52	.96
Mood dysregulation	2.07	0.62	1.00	3.00	-0.03	-1.13	.96

C.4 Bivariate Associations

Note. $N = 640$. ** $p < .01$. *** $p < .001$. The reward sensitivity–mood dysregulation correlation is $r = .944$, corresponding to approximately 89.2% shared variance (r^2). This exceptionally high overlap warrants caution about discriminant validity and same-source measurement.

Table 10: Pearson correlations among the main variables

**Variable**	**1**	**2**	**3**	**4**
1. Age	—			
2. Total screen time	.13**	—		
3. Reward sensitivity	.15***	.63***	—	
4. Mood dysregulation	.05	.53***	.94***	—

C.5 Group Differences

Table 11: Group differences by gender and school type

**Comparison**	**Outcome**	**Group means**	**Test**	p	**Effect size**
Gender	Total screen time	Male = 10.42; Female = 9.50	$t(638) = 3.14$	.002	$d = 0.25$
Gender	Reward sensitivity	Male = 2.93; Female = 3.16	$t(638) = -4.05$	$< .001$	$d = -0.32$
Gender	Mood dysregulation	Male = 1.89; Female = 2.25	$t(627.26) = -7.62$	$< .001$	$d = -0.60$
School type	Total screen time	Public = 8.54; Private/International = 12.86	$t(371.86) = -15.47$	$< .001$	$d = -1.36$
School type	Reward sensitivity	Public = 2.67; Private/International = 3.81	$t(632.85) = -31.97$	$< .001$	$d = -2.22$
School type	Mood dysregulation	Public = 1.79; Private/International = 2.65	$t(518.30) = -23.56$	$< .001$	$d = -1.83$

Table 12: Educational-level differences

**Outcome**	**Means**	**Test**	p	η^2
Total screen time	Middle = 8.41; High = 11.71; University = 8.64	$F(2, 637) = 68.22$	$< .001$	.18
Reward sensitivity	Middle = 3.04; High = 3.06; University = 3.03	$F(2, 637) = 0.14$	.873	$< .001$
Mood dysregulation	Middle = 2.17; High = 2.05; University = 2.03	$F(2, 637) = 2.63$	.073	.01

For total screen time, Tukey HSD post-hoc comparisons showed that high-school participants reported more screen time than both middle-school participants ($p < .001$) and university participants ($p < .001$); the middle-school versus university difference was not significant ($p = .800$).

C.6 Three-Step Hierarchical Multiple Regression

Reference groups were female, high school, private/international school, basic/secondary parental education, and semi-urban/rural residence.

Table 13: Three-step hierarchical regression predicting mean mood dysregulation

**Step**	**Predictor**	**B**	**SE**	*t*	*p*
1	Intercept	3.095	0.442	7.00	< .001
1	Male	-0.160	0.033	-4.85	< .001
1	Middle school	0.028	0.074	0.38	.703
1	University	0.180	0.076	2.38	.018
1	Public school	-0.387	0.042	-9.27	< .001
1	Parent education: postgraduate	0.950	0.055	17.20	< .001
1	Parent education: university	0.505	0.037	13.63	< .001
1	Urban residence	0.206	0.038	5.45	< .001
1	Age	-0.079	0.027	-2.87	.004
2	Intercept	2.680	0.435	6.17	< .001
2	Male	-0.226	0.034	-6.69	< .001
2	Middle school	0.142	0.075	1.91	.057
2	University	0.276	0.075	3.67	< .001
2	Public school	-0.320	0.042	-7.64	< .001
2	Parent education: postgraduate	0.779	0.060	12.89	< .001
2	Parent education: university	0.429	0.038	11.29	< .001
2	Urban residence	0.175	0.037	4.73	< .001
2	Age	-0.074	0.027	-2.77	.006
2	Total screen time	0.035	0.006	6.19	< .001
3	Intercept	0.277	0.214	1.29	.196
3	Male	-0.117	0.016	-7.14	< .001
3	Middle school	-0.007	0.036	-0.21	.837
3	University	0.119	0.036	3.30	.001
3	Public school	0.066	0.022	3.03	.003
3	Parent education: postgraduate	0.046	0.033	1.41	.160
3	Parent education: university	-0.110	0.022	-5.09	< .001
3	Urban residence	0.033	0.018	1.83	.068
3	Age	-0.047	0.013	-3.65	< .001
3	Total screen time	-0.004	0.003	-1.47	.141
3	Reward sensitivity	0.850	0.018	46.15	< .001

Table 14: Regression model fit and change statistics

Step	R^2	Adj. R^2	Model F	ΔR^2	ΔF
1	0.695	0.692	$F(8, 631) = 180.09$	—	—
2	0.713	0.709	$F(9, 630) = 173.80$	0.017	$F(1, 630) = 38.30$
3	0.935	0.933	$F(10, 629) = 897.88$	0.222	$F(1, 629) = 2129.60$

Step 1 explained 69.5% of the variance in mood dysregulation. Adding total screen time at Step 2 increased explained variance by 1.7%; at that stage, total screen time was positively associated with mood dysregulation ($B = .035, SE = .006, t = 6.19, p < .001$). Adding reward sensitivity at Step 3 increased explained variance by a further 22.2%. In the final model, reward sensitivity was strongly associated with mood dysregulation ($B = .850, SE = .018, t = 46.15, p < .001$), while total screen time became small and nonsignificant ($B = -.004, SE = .003, t = -1.47, p = .141$). This is a statistical attenuation pattern and does not establish temporal mediation.

C.7 Collinearity Diagnostic and Sensitivity Analysis

Table 15: Variance-inflation factors in the full Step 3 model

Predictor	VIF
Male	1.65
Middle school	5.94
University	6.77
Public school	2.58
Parent education: postgraduate	3.71
Parent education: university	2.86
Urban residence	1.79
Age	15.51
Total screen time	2.85
Reward sensitivity	4.54

Age had the largest VIF (15.51), reflecting its structural overlap with educational level. A diagnostic model omitting age reduced all VIFs to 4.53 or lower. In that age-omitted model, $R^2 = .933$; total screen time remained nonsignificant ($B = -.004, SE = .003, p = .160$), while reward sensitivity remained strongly associated with mood dysregulation ($B = .853, SE = .019, p < .001$). The focal attenuation pattern therefore does not depend on simultaneous adjustment for both age and educational level.

C.8 Statistical Interpretation Summary

The recalculated results support five descriptive/inferential conclusions: (1) mean daily screen exposure was approximately 9.96 hours; (2) screen time correlated with reward sensitivity ($r = .63$) and mood dysregulation ($r = .53$); (3) reward sensitivity and mood dysregulation were extremely highly correlated ($r = .94$), requiring caution about construct distinctiveness; (4) total screen time contributed a modest adjusted increment before reward sensitivity was entered but was nonsignificant after reward sensitivity entered the model; and (5) the focal pattern remained in the collinearity sensitivity analysis. Because the design is cross-sectional and all focal psychological measures are self-reported, these results are associations and should not be interpreted as demonstrating causal direction.
